# Supplementary material for: Quantum Chemical and Trajectory Surface Hopping Molecular Dynamics Study of Iodine‐Based BODIPY Photosensitizer
Source: J Comput Chem. 2025 Mar 11;46(7):e70026. doi: 10.1002/jcc.70026 (PMC11896635; doi:10.1002/jcc.70026)
Supplement: Supplementary file 1 — Data S1. Supporting Information contains (i) tables of characters (types), vertical excitation energies, oscillator strengths, and orbital overlaps of 10 lowest excited singlets and 10 triplets of two molecules, mono‐iodinated BODIPY in the position 2 (in its optimized S0 geometry) and I‐BODIPY (in its optimized S0, S1 and T2 geometries), calculated by CAS(18,15)PT2 (only for the 1st molecule), ADC(2), and TD‐DFT/TDA using several functionals, (ii) graphs of vertical excitation energies along the S1 to T2 PEC for the 2nd molecule calculated by ADC(2), TD‐DFT/B3LYP, and TD‐DFT(TDA)/BHLYP, (iii) tables of non‐parallelities between ADC(2) and TD‐DFT(TDA)/BHLYP along the PEC for the 2nd molecule, (iv) figures with shapes of frontier MOs of the 2nd molecule, (v) tables of SOCs between 11 lowest singlets and 10 lowest triplets of both molecules calculated by CAS(18,15)SCF (only for the 1st molecule, using 4 different spin‐orbit Hamiltonians), ADC(2), and TD‐DFT/TDA using several functionals, (vi) average populations of singlets and triplets in the dynamics of the 2nd molecule for three different values of the scaling factor α and extrapolations of lifetimes and time‐constants of singlet populations to α=1, (vii) histograms of the frequency of occurrence of the C(2)–B–C(8)–C(6) dihedral angle in the 2nd molecule at the time steps at which surface hops with a (possibly partial) Si→Sj, Si→Tk, Tk→Si or Tk→Tl character took place for all pairs of spin‐diabatic states, (viii) figures with shapes of the ground‐state electron density and differential electron density between S1 and S0 of the 2nd molecule, (ix) tables with optimized S0, S1 and T2 geometries of the 2nd molecule, (x) derivation of the formulae for the evaluation of matrix elements of the one‐electron one‐center effective spin‐orbit Hamiltonian implicitly included in a two‐component pseudopotential between non‐redundant Cartesian Gaussian functions. [file JCC-46-0-s001.pdf]

Supporting Information

Quantum Chemical and Trajectory Surface Hopping Molecular Dynamics Study of Iodine-based BODIPY Photosensitizer

Mirza Wasif Baig,[1,2] Marek Pederzoli,[1] Mojmír Kývala,[3] Jiří Pittner,[1*]*

[1]J. Heyrovský Institute of Physical Chemistry of the Czech Academy of Sciences, Dolejškova 3, 18223 Prague 8, Czech Republic

[2]Faculty of Science, Department of Physical and Macromolecular Chemistry, Charles University, Hlavova 8, 12840 Prague, Czech Republic

[3]Institute of Organic Chemistry and Biochemistry of the Czech Academy of Sciences, Flemingovo Nám. 2, 16000 Prague 6, Czech Republic

List of electronic structure methods used in the manuscript.

- DHK2 CASSCF and DHK2 CASPT2 using TZP contracted ANO-RCC basis set
- ADC(2) using dhf-TZVP basis set
- TD-DFT with three different functionals B3LYP, BHLYP and M06-2X using aug-cc-pVDZ, aug-cc-pVDZ-PP and dhf-TZVP basis sets
- TD-DFT(TDA) with B3LYP and BHLYP functionals using dhf-TZVP basis set
- sf-X2C-S-TD-DFT with B3LYP, BHLYP(TDA) and M06-2X functionals employing x2c-TZVPPall basis set.

Table S1a. Vertical excitation energies [eV] for singlets and triplets of monoiodinated BODIPY in the position 2 at CASPT2(18,15)/ANO-RCC level in optimized S_0 geometry

State	Character	Excitation energy [eV]	Symmetry
S_1	1(π, π*)	2.57	2SA'
S_2	2(π, π*)	3.65	3SA'
S_3	3(π, π*)	3.68	4SA'
S_4	4(π, π*)	4.28	5SA'
S_5	1(π, σ*)	4.84	1SA''
S_6	2(π, σ*)	4.85	2SA''
S_7	5(π, π*)	5.48	6SA'
S_8	6(π, π*)	5.55	3SA''
S_9	1(n, π*)	5.95	7SA'
S_{10}	7(π, π*)	6.18	8SA'

State	Character	Excitation energy [eV]	Symmetry
T_1	1(π, π*)	1.90	1TA'
T_2	2(π, π*)	2.96	2TA'
T_3	3(π, π*)	3.19	3TA'
T_4	4(π, π*)	3.87	4TA'
T_5	1(π, σ*)	4.31	1TA"
T_6	1(n, σ*)	4.69	5TA'
T_7	5(π, π*)	4.94	2TA"
T_8	2(π, σ*)	5.10	6TA'
T_9	6(π, π*)	5.37	7TA'
T_{10}	7(π, π*)	5.56	3TA"

Table S1b. Vertical excitation energies [eV] for singlets and triplets of monoiodinated BODIPY in the position 2 at ADC(2)/dhf-TZVP level in optimized S_0 geometry

State	Character	Excitation energy [eV]	Symmetry	Oscillator Strength	Orbital overlap
S_1	1(π, π*)	2.64	2SA'	0.53	0.67
S_2	2(π, π*)	3.68	3SA'	0.23	0.57
S_3	3(π, π*)	3.82	4SA'	0.15	0.56
S_4	1(n, π*)	4.71	1SA"	0.00	0.15
S_5	1(π, σ*)	4.81	2SA"	0.00	0.41
S_6	4(π, π*)	5.03	5SA'	0.02	0.74
S_7	1(n, σ*)	5.58	6SA'	0.01	0.57
S_8	5(π, π*)	5.62	7SA'	0.02	0.64
S_9	1(σ, π*)	5.72	8SA'	0.00	0.42
S_{10}	2(π, σ*)	5.93	3SA"	0.18	0.42

State	Character	Excitation energy [eV]	Symmetry	Orbital overlap
T_1	1(π, π*)	1.96	1TA'	0.69
T_2	2(π, π*)	2.99	2TA'	0.56
T_3	3(π, π*)	3.27	3TA'	0.58
T_4	4(π, π*)	4.03	4TA'	0.72
T_5	1(n, π*)	4.34	1TA"	0.43
T_6	1(π, σ*)	4.70	2TA"	0.15
T_7	5(π, π*)	4.93	5TA'	0.54
T_8	1(n, σ*)	5.39	6TA'	0.67
T_9	6(π, π*)	5.49	7TA'	0.71
T_{10}	2(π, σ*)	5.66	8TA'	0.42

Table S1c. Vertical excitation energies [eV] for singlets and triplets of monoiodinated BODIPY in the position 2 at TD-DFT/B3LYP-D3/dhf-TZVP level in optimized S_0 geometry

State	Character	Excitation energy [eV]	Symmetry	Oscillator Strength	Orbital overlap
S_1	1(π, π*)	2.76	2SA'	0.28	0.67
S_2	2(π, π*)	3.43	3SA'	0.32	0.62
S_3	1(n, π*)	3.53	1SA"	0.00	0.16
S_4	3(π, π*)	3.66	4SA'	0.13	0.53
S_5	1(π, σ*)	4.33	2SA"	0.00	0.43
S_6	4(π, π*)	4.42	5SA'	0.00	0.69
S_7	2(π, σ*)	5.09	3SA"	0.00	0.48
S_8	1(n, σ*)	5.29	6SA'	0.00	0.6
S_9	5(π, π*)	5.31	7SA'	0.02	0.74

State	Character	Excitation energy [eV]	Symmetry	Orbital overlap
T_1	1(π, π*)	1.54	1TA'	0.68
T_2	2(π, π*)	2.48	2TA'	0.63
T_3	3(π, π*)	2.84	3TA'	0.53
T_4	4(π, π*)	3.45	4TA'	0.70
T_5	1(n, π*)	3.51	1TA"	0.16
T_6	1(π, σ*)	3.90	2TA"	0.44
T_7	5(π, π*)	4.35	5TA'	0.73
T_8	1(n, σ*)	4.57	6TA'	0.59
T_9	6(π, π*)	4.76	7TA'	0.76
T_{10}	2(π, σ*)	4.91	3TA"	0.47

Table S1d. Vertical excitation energies [eV] for singlets and triplets of monoiodinated BODIPY in the position 2 at TD-DFT/BHLYP-D3/dhf-TZVP level in optimized S_0 geometry

State	Character	Excitation energy [eV]	Symmetry	Oscillator Strength	Orbital overlap
S_1	1(π, π*)	3.04	2SA'	0.59	0.69
S_2	2(π, π*)	3.95	3SA'	0.16	0.58
S_3	3(π, π*)	4.24	4SA'	0.06	0.54
S_4	1(π, σ*)	4.70	1SA''	0.00	0.41
S_5	1(n, π*)	4.74	2SA''	0.00	0.15
S_6	4(π, π*)	5.33	5SA'	0.00	0.71
S_7	1(n, σ*)	5.56	6SA'	0.00	0.58
S_8	2(π, σ*)	5.90	3SA''	0.00	0.46
S_9	5(π, π*)	5.98	7SA'	0.05	0.73
S_{10}	6(π, π*)	6.39	8SA'	0.14	0.76

State	Character	Excitation energy	Symmetry	Orbital overlap
T_1	1(π, π*)	1.19	1TA'	0.68
T_2	2(π, π*)	2.73	2TA'	0.59
T_3	3(π, π*)	2.97	3TA'	0.57
T_4	4(π, π*)	3.45	4TA'	0.70
T_5	1(π, σ*)	4.12	1TA''	0.43
T_6	1(n, π*)	4.70	2TA''	0.15
T_7	1(n, σ*)	4.74	5TA'	0.58
T_8	5(π, π*)	4.86	6TA'	0.74
T_9	6(π, π*)	5.22	7TA'	0.70
T_{10}	7(π, π*)	5.38	8TA'	0.70

Table S1e. Vertical excitation energies [eV] for singlets and triplets of monoiodinated BODIPY in the position 2 at TD-DFT/M06-2X-D3/dhf-TZVP level in optimized S_0 geometry

State	Character	Excitation energy [eV]	Symmetry	Oscillator Strength	Orbital overlap
S_1	1(π, π*)	2.99	2SA'	0.52	0.68
S_2	2(π, π*)	3.90	3SA'	0.17	0.59
S_3	3(π, π*)	4.17	4SA'	0.06	0.54
S_4	1(n, π*)	4.62	1SA''	0.00	0.16
S_5	1(π, σ*)	4.75	2SA''	0.00	0.43
S_6	4(π, π*)	5.27	5SA'	0.00	0.70
S_7	1(n, σ*)	5.51	6SA'	0.00	0.59
S_8	2(π, σ*)	5.91	3SA''	0.00	0.46
S_9	5(π, π*)	5.97	7SA'	0.06	0.74
S_{10}	1(σ, π*)	6.17	4SA''	0.14	0.39

State	Character	Excitation energy [eV]	Symmetry	Orbital overlap
T_1	1(π, π*)	1.52	1TA'	0.68
T_2	2(π, π*)	3.02	2TA'	0.59
T_3	3(π, π*)	3.30	3TA'	0.55
T_4	4(π, π*)	3.86	4TA'	0.72
T_5	1(π, σ*)	4.31	1TA''	0.44
T_6	1(n, π*)	4.59	2TA''	0.16
T_7	1(n, σ*)	4.87	5TA'	0.59
T_8	5(π, π*)	5.25	6TA'	0.72
T_9	6(π, π*)	5.42	7TA'	0.75
T_{10}	7(π, π*)	5.74	8TA'	0.73

Table S1f. Vertical excitation energies [eV] for singlets and triplets of monoiodinated BODIPY in the position 2 at TD-DFT(TDA)/B3LYP-D3/dhf-TZVP level in optimized S_0 geometry

State	Character	Excitation energy [eV]	Symmetry	Oscillator Strength	Orbital overlap
S_1	1(π, π*)	2.94	2SA'	0.25	0.66
S_2	1(n, π*)	3.53	1SA"	0.00	0.16
S_3	2(π, π*)	3.68	3SA'	0.02	0.57
S_4	3(π, π*)	3.91	4SA'	0.06	0.6
S_5	1(π, σ*)	4.37	2SA"	0.00	0.43
S_6	4(π, π*)	4.48	5SA'	0.00	0.69
S_7	2(π, σ*)	5.10	3SA"	0.00	0.48
S_8	1(σ, π*)	5.32	4SA"	0.00	0.36
S_9	1(n, σ*)	5.35	6SA'	0.00	0.59
S_{10}	5(π, π*)	5.44	7SA'	0.01	0.74

State	Character	Excitation energiy [eV]	Symmetry	Orbital overlap
T_1	1(π, π*)	1.66	1TA'	0.68
T_2	2(π, π*)	2.58	2TA'	0.63
T_3	3(π, π*)	2.94	3TA'	0.53
T_4	1(n, π*)	3.51	1TA"	0.16
T_5	4(π, π*)	3.58	4TA'	0.70
T_6	1(π, σ*)	3.94	2TA"	0.44
T_7	5(π, π*)	4.39	5TA'	0.73
T_8	1(n, σ*)	4.62	6TA'	0.59
T_9	6(π, π*)	4.87	7TA'	0.76
T_{10}	1(π, σ*)	4.92	3TA"	0.47

Table S1g. Vertical excitation energies [eV] for singlets and triplets of monoiodinated BODIPY in the position 2 at TD-DFT(TDA)/BHLYP-D3/dhf-TZVP level in optimized S_0 geometry

State	Character	Excitation energy [eV]	Symmetry	Oscillator Strength	Orbital overlap
S_1	1(π, π*)	3.32	2SA'	0.10	0.68
S_2	2(π, π*)	4.13	3SA'	0.04	0.59
S_3	3(π, π*)	4.39	4SA'	0.00	0.54
S_4	1(n, π*)	4.74	1SA"	0.00	0.23
S_5	1(π, σ*)	4.76	2SA"	0.00	0.34
S_6	4(π, π*)	5.43	5SA'	0.00	0.71
S_7	1(n, σ*)	5.63	6SA'	0.00	0.58
S_8	2(π, σ*)	5.91	3SA"	0.00	0.46
S_9	5(π, π*)	6.13	7SA'	0.00	0.74
S_{10}	1(σ, π*)	6.55	4SA"	0.00	0.37

State	Character	Excitation energy [eV]	Symmetry	Orbital overlap
T_1	1(π, π*)	1.63	1TA'	0.68
T_2	2(π, π*)	3.00	2TA'	0.59
T_3	3(π, π*)	3.26	3TA'	0.55
T_4	4(π, π*)	3.73	4TA'	0.72
T_5	1(π, σ*)	4.19	1TA"	0.44
T_6	1(n, π*)	4.71	2TA"	0.15
T_7	1(n, σ*)	4.82	5TA'	0.58
T_8	5(π, π*)	5.12	6TA'	0.75
T_9	6(π, π*)	5.34	7TA'	0.71
T_{10}	7(π, π*)	5.54	8TA'	0.72

Table S2a. Vertical excitation energies [eV] for singlets and triplets of I-BODIPY at ADC(2)/dhf-TZVP level in optimized S_0 geometry

State	Character	Excitation energy [eV]	Oscillator Strength	Orbital overlap
S_1	1(π, π*)	2.56	0.58	0.67
S_2	2(π, π*)	3.22	0.22	0.47
S_3	3(π, π*)	3.49	0.11	0.59
S_4	1(π, σ*)	4.39	0.00	0.37
S_5	1(n, π*)	4.52	0.00	0.15
S_6	2(π, σ*)	4.59	0.00	0.52
S_7	2(n, π*)	4.83	0.01	0.24
S_8	4(π, π*)	4.84	0.07	0.54

State	Character	Excitation energy [eV]	Orbital overlap
T_1	1(π, π*)	1.91	0.69
T_2	2(π, π*)	2.78	0.50
T_3	3(π, π*)	3.00	0.54
T_4	4(π, π*)	3.92	0.64
T_5	1(π, σ*)	4.09	0.47
T_6	2(π, σ*)	4.22	0.44
T_7	1(n, π*)	4.52	0.15
T_8	1(n, σ*)	4.71	0.50
T_9	2(n, π*)	4.81	0.21

Table S2b. Vertical excitation energies [eV] for singlets and triplets of I-BODIPY at ADC(2)/dhf-TZVP level in optimized S_1 geometry

State	Character	Excitation energy [eV]	Oscillator Strength	Orbital overlap
S_1	$1(\pi, \pi^*)$	2.13	0.17	0.56
S_2	$2(\pi, \pi^*)$	2.64	0.42	0.52
S_3	$3(\pi, \pi^*)$	3.37	0.07	0.63
S_4	$1(n, \pi^*)$	4.16	0.00	0.13
S_5	$2(n, \pi^*)$	4.41	0.00	0.18
S_6	$1(\pi, \sigma^*)$	4.47	0.00	0.48
S_7	$4(\pi, \pi^*)$	4.56	0.14	0.61
S_8	$2(\pi, \sigma^*)$	4.69	0.00	0.39

State	Character	Excitation energy [eV]	Orbital overlap
T_1	$1(\pi, \pi^*)$	1.82	0.61
T_2	$2(\pi, \pi^*)$	2.19	0.47
T_3	$3(\pi, \pi^*)$	2.78	0.63
T_4	$4(\pi, \pi^*)$	3.62	0.66
T_5	$1(\pi, \sigma^*)$	4.11	0.50
T_6	$1(n, \pi^*)$	4.16	0.13
T_7	$2(\pi, \sigma^*)$	4.31	0.39
T_8	$2(n, \pi^*)$	4.40	0.19
T_9	$1(n, \sigma^*)$	4.85	0.49

Table S2c. Vertical excitation energies [eV] for singlets and triplets of I-BODIPY at ADC(2)/dhf-TZVP level in optimized T_2 geometry

State	Character	Excitation energy [eV]	Oscillator Strength	Orbital overlap
S_1	1(*π*, *π**)	2.52	0.56	0.33
S_2	2(*π*, *π**)	3.18	0.22	0.47
S_3	3(*π*, *π**)	3.45	0.10	0.60
S_4	1(*π*, σ*)	4.31	0.00	0.60
S_5	1(n, *π**)	4.50	0.00	0.15
S_6	2(*π*, σ*)	4.51	0.00	0.52
S_7	4(*π*, *π**)	4.79	0.07	0.52
S_8	2(n, *π**)	4.80	0.01	0.26

State	Character	Excitation energy [eV]	Orbital overlap
T_1	1(*π*, *π**)	1.88	0.69
T_2	2(*π*, *π**)	2.76	0.50
T_3	3(*π*, *π**)	2.97	0.54
T_4	4(*π*, *π**)	3.87	0.64
T_5	1(*π*, σ*)	4.01	0.46
T_6	2(*π*, σ*)	4.13	0.43
T_7	1(n, *π**)	4.50	0.14
T_8	1(n, σ*)	4.62	0.47
T_9	2(n, *π**)	4.78	0.21

Table S3a. Vertical excitation energies [eV] for singlets and triplets of I-BODIPY at TD-DFT/B3LYP-D3/dhf-TZVP level in optimized S_0 geometry

State	Character	Excitation energy [eV]	Oscillator Strength	Orbital overlap
S_1	1(π, π*)	2.75	0.33	0.65
S_2	2(π, π*)	3.01	0.37	0.55
S_3	3(π, π*)	3.36	0.08	0.54
S_4	1(n, π*)	3.52	0.00	0.17
S_5	1(π, σ*)	3.79	0.00	0.35
S_6	2(n, π*)	3.92	0.00	0.24
S_7	4(π, π*)	4.11	0.05	0.58
S_8	2(π, σ*)	4.12	0.00	0.61
S_9	3(π, σ*)	4.66	0.00	0.40
S_{10}	1(n, σ*)	4.85	0.00	0.58

State	Character	Excitation energy [eV]	Orbital overlap
T_1	1(π, π*)	1.44	0.70
T_2	2(π, π*)	1.89	0.51
T_3	3(π, π*)	2.41	0.53
T_4	4(π, π*)	3.04	0.62
T_5	1(n, π*)	3.17	0.17
T_6	2(n, π*)	3.54	0.40
T_7	1(π, σ*)	3.65	0.55
T_8	2(π, σ*)	3.86	0.24
T_9	5(π, π*)	3.99	0.68
T_{10}	6(π, π*)	4.19	0.56

Table S3b. Vertical excitation energies [eV] for singlets and triplets of I-BODIPY at TD-DFT/B3LYP-D3/dhf-TZVP level in optimized S_1 geometry

State	Character	Excitation energy [eV]	Oscillator Strength	Orbital overlap
S_1	1(π, π*)	2.25	0.06	0.60
S_2	2(π, π*)	2.77	0.55	0.62
S_3	1(n, π*)	3.19	0.00	0.16
S_4	3(π, π*)	3.26	0.04	0.54
S_5	2(n, π*)	3.58	0.00	0.20
S_6	4(π, π*)	3.88	0.09	0.62
S_7	1(π, σ*)	3.98	0.00	0.35
S_8	2(π, σ*)	4.20	0.00	0.52
S_9	5(π, π*)	4.41	0.02	0.47
S_{10}	1(π, σ*)	4.64	0.00	0.48

State	Character	Excitation energy [eV]	Orbital overlap
T_1	1(π, π*)	1.51	0.60
T_2	2(π, π*)	2.40	0.62
T_3	3(π, π*)	2.59	0.54
T_4	4(π, π*)	3.33	0.63
T_5	1(n, π*)	3.50	0.16
T_6	1(π, σ*)	3.58	0.20
T_7	2(π, σ*)	3.72	0.45
T_8	2(n, π*)	3.89	0.45
T_9	5(π, π*)	4.20	0.56
T_{10}	1(n, σ*)	4.30	0.60

Table S3c. Vertical excitation energies [eV] for singlets and triplets of I-BODIPY at TD-DFT/B3LYP-D3/dhf-TZVP level in optimized T_2 geometry

State	Character	Excitation energy [eV]	Oscillator Strength	Orbital overlap
S_1	1(π, π*)	2.72	0.33	0.65
S_2	2(π, π*)	2.98	0.36	0.55
S_3	3(π, π*)	3.32	0.08	0.54
S_4	1(n, π*)	3.50	0.00	0.16
S_5	1(π, σ*)	3.71	0.00	0.35
S_6	2(n, π*)	3.88	0.00	0.22
S_7	2(π, σ*)	4.05	0.00	0.61
S_8	4(π, π*)	4.07	0.03	0.58
S_9	3(π, σ*)	4.56	0.00	0.41
S_{10}	1(n, σ*)	4.77	0.00	0.57

State	Character	Excitation energy [eV]	Orbital overlap
T_1	1(π, π*)	1.47	0.70
T_2	2(π, π*)	2.37	0.51
T_3	3(π, π*)	2.56	0.53
T_4	4(π, π*)	3.28	0.62
T_5	1(n, π*)	3.48	0.16
T_6	1(π, σ*)	3.50	0.40
T_7	2(π, σ*)	3.63	0.55
T_8	2(n, π*)	3.85	0.22
T_9	5(π, π*)	4.14	0.68
T_{10}	1(n, σ*)	4.22	0.56

Table S4a. Vertical excitation energies [eV] for singlets and triplets of I-BODIPY at TD-DFT/BHLYP-D3/dhf-TZVP level in optimized S_0 geometry

State	Character	Excitation energy [eV]	Oscillator Strength	Orbital Overlap
S_1	1(π, π*)	2.96	0.74	0.70
S_2	2(π, π*)	3.66	0.10	0.48
S_3	3(π, π*)	3.93	0.08	0.55
S_4	1(π, σ*)	4.37	0.00	0.38
S_5	2(π, σ*)	4.54	0.00	0.55
S_6	1(n, π*)	4.65	0.00	0.16
S_7	2(n, π*)	5.04	0.00	0.23
S_8	4(π, π*)	5.05	0.03	0.54
S_9	1(n, σ*)	5.31	0.00	0.55
S_{10}	3(π, σ*)	5.38	0.00	0.46

State	Character	Excitation energy [eV]	Orbital Overlap
T_1	1(π, π*)	1.14	0.69
T_2	2(π, π*)	2.61	0.54
T_3	3(π, π*)	2.88	0.51
T_4	4(π, π*)	3.45	0.66
T_5	1(π, σ*)	3.91	0.50
T_6	2(π, σ*)	4.07	0.47
T_7	1(n, σ*)	4.56	0.55
T_8	1(n, π*)	4.62	0.16
T_9	5(π, π*)	4.67	0.65
T_{10}	2(n, σ*)	4.74	0.58

Table S4b. Vertical excitation energies [eV] for singlets and triplets of I-BODIPY at TD-DFT/BHLYP-D3/dhf-TZVP level in optimized S_1 geometry

State	Character	Excitation energy [eV]	Oscillator Strength	Orbital Overlap
S_1	1(π, π*)	2.79	0.99	0.60
S_2	2(π, π*)	3.16	0.00	0.55
S_3	3(π, π*)	3.77	0.27	0.58
S_4	1(n, π*)	4.34	0.38	0.15
S_5	1(π, σ*)	4.48	0.02	0.49
S_6	2(π, σ*)	4.64	0.00	0.42
S_7	2(n, π*)	4.69	0.00	0.18
S_8	4(π, π*)	4.78	0.30	0.60
S_9	3(π, σ*)	5.31	0.00	0.40
S_{10}	5(π, π*)	5.38	0.00	0.49

State	Character	Excitation energy [eV]	Orbital Overlap
T_1	1(π, π*)	1.12	0.59
T_2	2(π, π*)	2.29	0.57
T_3	3(π, π*)	2.50	0.56
T_4	4(π, π*)	3.19	0.63
T_5	1(π, σ*)	3.96	0.52
T_6	2(π, σ*)	4.13	0.43
T_7	1(n, π*)	4.32	0.15
T_8	5(π, π*)	4.50	0.57
T_9	2(n, π*)	4.65	0.18
T_{10}	1(n, σ*)	4.70	0.53

Table S4c. Vertical excitation energies [eV] for singlets and triplets of I-BODIPY at TD-DFT/BHLYP-D3/dhf-TZVP level in optimized T_2 geometry

State	Character	Excitation energy [eV]	Oscillator Strength	Orbital Overlap
S_1	1(π, π*)	2.92	0.73	0.70
S_2	2(π, π*)	3.64	0.10	0.48
S_3	3(π, π*)	3.90	0.08	0.55
S_4	1(π, σ*)	4.29	0.00	0.38
S_5	2(π, σ*)	4.46	0.00	0.55
S_6	1(n, π*)	4.63	0.00	0.16
S_7	4(π, π*)	5.01	0.03	0.55
S_8	2(n, π*)	5.02	0.00	0.21
S_9	1(n, σ*)	5.22	0.00	0.55
S_{10}	3(π, σ*)	5.28	0.00	0.46

State	Character	Excitation energy [eV]	Orbital Overlap
T_1	1(π, π*)	1.09	0.69
T_2	2(π, π*)	2.56	0.54
T_3	3(π, π*)	2.85	0.51
T_4	4(π, π*)	3.39	0.66
T_5	1(π, σ*)	3.83	0.50
T_6	2(π, σ*)	3.98	0.47
T_7	1(n, σ*)	4.47	0.55
T_8	5(π, π*)	4.60	0.64
T_9	1(n, π*)	4.60	0.17
T_{10}	2(n, σ*)	4.65	0.58

Table S5a. Vertical excitation energies [eV] for singlets and triplets of I-BODIPY at TD-DFT/M06-2X-D3/dhf-TZVP level in optimized S_0 geometry

State	Character	Excitation energy [eV]	Oscillator Strength	Orbital overlap
S_1	1(π, π*)	2.91	0.66	0.70
S_2	2(π, π*)	3.57	0.11	0.48
S_3	3(π, π*)	3.83	0.07	0.54
S_4	1(π, σ*)	4.40	0.00	0.39
S_5	1(n, π*)	4.54	0.00	0.18
S_6	2(π, σ*)	4.56	0.00	0.54
S_7	2(n, π*)	4.89	0.00	0.21
S_8	4(π, π*)	5.00	0.02	0.57
S_9	1(n, σ*)	5.25	0.00	0.56
S_{10}	3(π, σ*)	5.38	0.00	0.47

State	Character	Excitation energy [eV]	Orbital overlap
T_1	1(π, π*)	1.51	0.70
T_2	2(π, π*)	2.87	0.52
T_3	3(π, π*)	3.08	0.51
T_4	4(π, π*)	3.81	0.66
T_5	1(π, σ*)	4.06	0.50
T_6	2(π, σ*)	4.21	0.48
T_7	1(n, π*)	4.52	0.17
T_8	1(n, σ*)	4.66	0.56
T_9	2(n, σ*)	4.86	0.58
T_{10}	2(n, π*)	4.87	0.21

Table S5b. Vertical excitation energies [eV] for singlets and triplets of I-BODIPY at TD-DFT/M06-2X-D3/dhf-TZVP level in optimized S_1 geometry

State	Character	Excitation energy [eV]	Oscillator Strength	Orbital overlap
S_1	1(π, π*)	2.72	0.34	0.59
S_2	2(π, π*)	3.09	0.34	0.56
S_3	3(π, π*)	3.67	0.06	0.57
S_4	1(n, π*)	4.24	0.00	0.16
S_5	1(π, σ*)	4.50	0.00	0.47
S_6	2(n, π*)	4.54	0.00	0.19
S_7	1(π, σ*)	4.67	0.00	0.44
S_8	4(π, π*)	4.73	0.05	0.60
S_9	2(π, σ*)	5.32	0.00	0.41
S_{10}	5(π, π*)	5.34	0.02	0.47

State	Character	Excitation energy [eV]	Orbital overlap
T_1	1(π, π*)	1.52	0.59
T_2	2(π, π*)	2.45	0.57
T_3	3(π, π*)	2.77	0.57
T_4	4(π, π*)	3.55	0.63
T_5	1(π, σ*)	4.12	0.52
T_6	1(n, π*)	4.23	0.16
T_7	2(π, σ*)	4.30	0.43
T_8	2(n, π*)	4.52	0.19
T_9	1(n, σ*)	4.81	0.54
T_{10}	5(π, π*)	4.89	0.56

Table S5c. Vertical excitation energies [eV] for singlets and triplets of I-BODIPY at TD-DFT/M06-2X-D3/dhf-TZVP level in optimized T_2 geometry

State	Character	Excitation energy [eV]	Oscillator Strength	Orbital overlap
S_1	1(π, π*)	2.88	0.65	0.70
S_2	2(π, π*)	3.55	0.11	0.48
S_3	3(π, π*)	3.79	0.07	0.54
S_4	1(π, σ*)	4.31	0.00	0.39
S_5	2(π, σ*)	4.48	0.00	0.55
S_6	1(n, π*)	4.52	0.00	0.17
S_7	2(n, π*)	4.87	0.00	0.20
S_8	4(π, π*)	4.95	0.02	0.56
S_9	1(n, σ*)	5.16	0.00	0.55
S_{10}	3(π, σ*)	5.27	0.00	0.48

State	Character	Excitation energy [eV]	Orbital overlap
T_1	1(π, π*)	1.47	0.70
T_2	2(π, π*)	2.84	0.52
T_3	3(π, π*)	3.06	0.50
T_4	4(π, π*)	3.75	0.66
T_5	1(π, σ*)	3.98	0.50
T_6	2(π, σ*)	4.12	0.48
T_7	1(n, π*)	4.50	0.17
T_8	1(n, σ*)	4.57	0.55
T_9	2(n, σ*)	4.76	0.59
T_{10}	2(n, π*)	4.84	0.20

Table S6a. Vertical excitation energies [eV] for singlets and triplets of I-BODIPY at TD-DFT(TDA)/B3LYP-D3/dhf-TZVP level in optimized S_0 geometry

State	Character	Excitation energy [eV]	Oscillator Strength	Orbital overlap
S_1	$1(\pi, \pi^*)$	2.90	0.00	0.56
S_2	$2(\pi, \pi^*)$	3.24	0.12	0.61
S_3	$3(\pi, \pi^*)$	3.50	0.02	0.55
S_4	$1(n, \pi^*)$	3.52	0.00	0.17
S_5	$1(\pi, \sigma^*)$	3.80	0.00	0.35
S_6	$2(n, \pi^*)$	3.92	0.00	0.24
S_7	$2(\pi, \sigma^*)$	4.16	0.00	0.61
S_8	$4(\pi, \pi^*)$	4.22	0.06	0.58
S_9	$3(\pi, \sigma^*)$	4.68	0.00	0.40
S_{10}	$1(n, \sigma^*)$	4.89	0.00	0.57

State	Character	Excitation energy [eV]	Orbital overlap
T_1	$1(\pi, \pi^*)$	1.63	0.71
T_2	$2(\pi, \pi^*)$	2.47	0.51
T_3	$3(\pi, \pi^*)$	2.69	0.53
T_4	$4(\pi, \pi^*)$	3.44	0.62
T_5	$1(n, \pi^*)$	3.50	0.17
T_6	$1(\pi, \sigma^*)$	3.61	0.40
T_7	$2(\pi, \sigma^*)$	3.75	0.56
T_8	$2(n, \pi^*)$	3.89	0.24
T_9	$5(\pi, \pi^*)$	4.25	0.69
T_{10}	$1(n, \sigma^*)$	4.34	0.56

Table S6b. Vertical excitation energies [eV] for singlets and triplets of I-BODIPY at TD-DFT(TDA)/B3LYP-D3/dhf-TZVP level in optimized S_1 geometry

State	Character	Excitation energy [eV]	Oscillator Strength	Orbital overlap
S_1	1(π, π*)	2.34	0.03	0.60
S_2	2(π, π*)	3.03	0.10	0.61
S_3	1(n, π*)	3.19	0.00	0.16
S_4	3(π, π*)	3.39	0.02	0.54
S_5	2(n, π*)	3.58	0.00	0.20
S_6	1(π, σ*)	3.99	0.00	0.35
S_7	4(π, π*)	4.00	0.08	0.62
S_8	2(π, σ*)	4.23	0.00	0.53
S_9	5(π, π*)	4.45	0.01	0.47
S_{10}	3(π, σ*)	4.67	0.00	0.48

State	Character	Excitation energy [eV]	Orbital overlap
T_1	1(π, π*)	1.56	0.60
T_2	2(π, π*)	1.96	0.62
T_3	3(π, π*)	2.52	0.54
T_4	4(π, π*)	3.16	0.63
T_5	1(n, π*)	3.18	0.16
T_6	2(n, π*)	3.55	0.20
T_7	1(π, σ*)	3.68	0.45
T_8	2(π, σ*)	3.89	0.45
T_9	5(π, π*)	4.03	0.57
T_{10}	6(π, π*)	4.22	0.59

Table S6c. Vertical excitation energies [eV] for singlets and triplets of I-BODIPY at TD-DFT(TDA)/B3LYP-D3/dhf-TZVP level in optimized T_2 geometry

State	Character	Excitation energy [eV]	Oscillator Strength	Orbital overlap
S_1	1(π, π*)	2.87	0.00	0.56
S_2	2(π, π*)	3.21	0.12	0.61
S_3	3(π, π*)	3.46	0.02	0.56
S_4	1(n, π*)	3.50	0.00	0.16
S_5	1(π, σ*)	3.72	0.00	0.35
S_6	2(n, π*)	3.88	0.00	0.22
S_7	2(π, σ*)	4.08	0.00	0.61
S_8	4(π, π*)	4.17	0.06	0.58
S_9	3(π, σ*)	4.57	0.00	0.40
S_{10}	1(n, σ*)	4.89	0.00	0.57

State	Character	Excitation energy [eV]	Orbital overlap
T_1	1(π, π*)	1.60	0.71
T_2	2(π, π*)	2.44	0.51
T_3	3(π, π*)	2.65	0.53
T_4	4(π, π*)	3.39	0.62
T_5	1(n, π*)	3.48	0.16
T_6	1(π, σ*)	3.53	0.39
T_7	2(π, σ*)	3.66	0.56
T_8	2(n, π*)	3.85	0.22
T_9	5(π, π*)	4.19	0.69
T_{10}	1(n, σ*)	4.26	0.56

Table S7a. Vertical excitation energies [eV] for singlets and triplets of I-BODIPY at TD-DFT(TDA)/BHLYP-D3/dhf-TZVP level in optimized S_0 geometry

State	Character	Excitation energy [eV]	Oscillator Strength	Orbital overlap
S_1	1(π, π*)	3.23	0.29	0.69
S_2	2(π, π*)	3.77	0.06	0.48
S_3	3(π, π*)	4.07	0.01	0.55
S_4	1(π, σ*)	4.41	0.00	0.38
S_5	2(π, σ*)	4.59	0.00	0.56
S_6	1(n, π*)	4.65	0.00	0.16
S_7	2(n, π*)	5.05	0.00	0.20
S_8	4(π, π*)	5.15	0.02	0.57
S_9	1(n, σ*)	5.37	0.00	0.55
S_{10}	3(π, σ*)	5.41	0.00	0.46

State	Character	Excitation energy [eV]	Orbital overlap
T_1	1(π, π*)	1.61	0.70
T_2	2(π, π*)	2.88	0.53
T_3	3(π, π*)	3.10	0.50
T_4	4(π, π*)	3.71	0.67
T_5	1(π, σ*)	3.98	0.50
T_6	2(π, σ*)	4.13	0.47
T_7	1(n, π*)	4.63	0.16
T_8	1(n, σ*)	4.64	0.55
T_9	2(n, σ*)	4.82	0.58
T_{10}	5(π, π*)	4.93	0.66

Table S7b. Vertical excitation energies [eV] for singlets and triplets of I-BODIPY at TD-DFT(TDA)/BHLYP-D3/dhf-TZVP level in optimized S_1 geometry

State	Character	Excitation energy [eV]	Oscillator Strength	Orbital overlap
S_1	1(π, π*)	2.96	0.08	0.60
S_2	2(π, π*)	3.33	0.19	0.56
S_3	3(π, π*)	3.93	0.02	0.58
S_4	1(n, π*)	4.34	0.00	0.15
S_5	1(π, σ*)	4.53	0.00	0.48
S_6	2(π, σ*)	4.68	0.00	0.43
S_7	2(n, π*)	4.70	0.00	0.18
S_8	4(π, π*)	4.90	0.04	0.60
S_9	3(π, σ*)	5.33	0.00	0.41
S_{10}	5(π, π*)	5.48	0.01	0.48

State	Character	Excitation energy [eV]	Orbital overlap
T_1	1(π, π*)	1.60	0.60
T_2	2(π, π*)	2.50	0.56
T_3	3(π, π*)	2.77	0.57
T_4	4(π, π*)	3.46	0.63
T_5	1(π, σ*)	4.03	0.52
T_6	2(π, σ*)	4.20	0.43
T_7	1(n, π*)	4.32	0.15
T_8	2(n, π*)	4.66	0.18
T_9	1(n, σ*)	4.78	0.53
T_{10}	5(π, π*)	4.80	0.57

Table S7c. Vertical excitation energies [eV] for singlets and triplets of I-BODIPY at TD-DFT(TDA)/BHLYP-D3/dhf-TZVP level in optimized T_2 geometry

State	Character	Excitation energy [eV]	Oscillator Strength	Orbital overlap
S_1	1(π, π*)	3.20	0.29	0.69
S_2	2(π, π*)	3.75	0.06	0.48
S_3	3(π, π*)	4.04	0.01	0.55
S_4	1(π, σ*)	4.33	0.00	0.38
S_5	2(π, σ*)	4.51	0.00	0.56
S_6	1(n, π*)	4.63	0.00	0.16
S_7	2(n, π*)	5.02	0.00	0.20
S_8	4(π, π*)	5.11	0.02	0.56
S_9	1(n, σ*)	5.28	0.00	0.55
S_{10}	3(π, σ*)	5.31	0.00	0.47

State	Character	Excitation energy [eV]	Orbital overlap
T_1	1(π, π*)	1.58	0.70
T_2	2(π, π*)	2.84	0.53
T_3	3(π, π*)	3.07	0.50
T_4	4(π, π*)	3.65	0.67
T_5	1(π, σ*)	3.90	0.50
T_6	2(π, σ*)	4.04	0.47
T_7	1(n, σ*)	4.55	0.55
T_8	1(n, π*)	4.61	0.16
T_9	2(n, σ*)	4.73	0.58
T_{10}	5(π, π*)	4.87	0.66

Table S8a. Vertical excitation energies [eV] for singlets and triplets of I-BODIPY at sf-X2C-TD-DFT/B3LYP-D3/x2c-TZVPPall level in optimized S_0 geometry

State	Character	Excitation energy [eV]	Oscillator Strength
S_1	$1(\pi, \pi^*)$	2.74	0.30
S_2	$2(\pi, \pi^*)$	2.99	0.38
S_3	$3(\pi, \pi^*)$	3.36	0.08
S_4	$1(n, \pi^*)$	3.50	0.00
S_5	$1(\pi, \sigma^*)$	3.89	0.00
S_6	$2(n, \pi^*)$	3.90	0.00
S_7	$4(\pi, \pi^*)$	4.10	0.06
S_8	$2(\pi, \sigma^*)$	4.21	0.00
S_9	$3(\pi, \sigma^*)$	4.81	0.01
S_{10}	$1(n, \sigma^*)$	4.87	0.00

State	Character	Excitation energy [eV]
T_1	$1(\pi, \pi^*)$	1.51
T_2	$2(\pi, \pi^*)$	2.40
T_3	$3(\pi, \pi^*)$	2.59
T_4	$4(\pi, \pi^*)$	3.33
T_5	$1(n, \pi^*)$	3.48
T_6	$1(\pi, \sigma^*)$	3.67
T_7	$2(\pi, \sigma^*)$	3.80
T_8	$2(n, \pi^*)$	3.87
T_9	$5(\pi, \pi^*)$	4.19
T_{10}	$1(n, \sigma^*)$	4.37

Table S8b. Vertical excitation energies [eV] for singlets and triplets of I-BODIPY at sf-X2C-S-TD-DFT/B3LYP-D3/x2c-TZVPPall level at the optimized S_1 geometry

State	Character	Excitation energy [eV]	Oscillator Strength
S_1	1(π, π*)	2.23	0.06
S_2	2(π, π*)	2.77	0.54
S_3	1(n, π*)	3.17	0.00
S_4	3(π, π*)	3.26	0.04
S_5	2(n, π*)	3.57	0.00
S_6	4(π, π*)	3.87	0.09
S_7	1(π, σ*)	4.31	0.00
S_8	2(π, σ*)	4.39	0.00
S_9	5(π, π*)	4.78	0.02
S_{10}	1(π, σ*)	4.95	0.00

State	Character	Excitation energy [eV]
T_1	1(π, π*)	1.44
T_2	2(π, π*)	1.87
T_3	3(π, π*)	2.41
T_4	4(π, π*)	3.03
T_5	1(n, π*)	3.15
T_6	2(n, π*)	3.52
T_7	1(π, σ*)	3.73
T_8	2(π, σ*)	3.96
T_9	5(π, π*)	3.98
T_{10}	6(π, π*)	4.17

Table S8c. Vertical excitation energies [eV] for singlets and triplets of I-BODIPY at sf-X2C-S-TD-DFT/B3LYP-D3/x2c-TZVPPall level in optimized T_2 geometry

State	Character	Excitation energy [eV]	Oscillator Strength
S_1	1(π, π*)	2.71	0.31
S_2	2(π, π*)	2.97	0.37
S_3	3(π, π*)	3.32	0.08
S_4	1(n, π*)	3.48	0.00
S_5	1(π, σ*)	3.80	0.00
S_6	2(n, π*)	3.86	0.00
S_7	2(π, σ*)	4.05	0.05
S_8	4(π, π*)	4.13	0.00
S_9	3(π, σ*)	4.70	0.00
S_{10}	1(n, σ*)	4.80	0.01

State	Character	Excitation energy [eV]
T_1	1(π, π*)	1.47
T_2	2(π, π*)	2.35
T_3	3(π, π*)	2.56
T_4	4(π, π*)	3.27
T_5	1(n, π*)	3.46
T_6	1(π, σ*)	3.58
T_7	2(π, σ*)	3.71
T_8	2(n, π*)	3.83
T_9	5(π, π*)	4.13
T_{10}	1(n, σ*)	4.29

Table S9a. Vertical excitation energies [eV] for singlets and triplets of I-BODIPY at sf-X2C-TD-DFT(TDA)/BHLYP-D3/x2c-TZVPPall level in optimized S_0 geometry

State	Character	Excitation energy [eV]	Oscillator Strength
S_1	1(π, π*)	3.23	0.98
S_2	2(π, π*)	3.77	0.21
S_3	3(π, π*)	4.07	0.12
S_4	1(π, σ*)	4.51	0.00
S_5	2(π, σ*)	4.64	0.00
S_6	1(n, π*)	4.69	0.00
S_7	2(n, π*)	5.04	0.00
S_8	4(π, π*)	5.15	0.05
S_9	1(n, σ*)	5.47	0.00
S_{10}	3(π, σ*)	5.55	0.00

State	Character	Excitation energy [eV]
T_1	1(π, π*)	1.61
T_2	2(π, π*)	2.88
T_3	3(π, π*)	3.09
T_4	4(π, π*)	3.70
T_5	1(π, σ*)	4.05
T_6	2(π, σ*)	4.21
T_7	1(n, π*)	4.62
T_8	1(n, σ*)	4.71
T_9	2(n, σ*)	4.89
T_{10}	5(π, π*)	4.93

Table S9b. Vertical excitation energies [eV] for singlets and triplets of I-BODIPY at sf-X2C-TD-DFT(TDA)/BHLYP-D3/x2c-TZVPPall level in optimized S_1 geometry

State	Character	Excitation energy [eV]	Oscillator Strength
S_1	$1(\pi, \pi^*)$	2.95	0.36
S_2	$2(\pi, \pi^*)$	3.33	0.68
S_3	$3(\pi, \pi^*)$	3.93	0.09
S_4	$1(n, \pi^*)$	4.33	0.00
S_5	$1(\pi, \sigma^*)$	4.64	0.00
S_6	$2(\pi, \sigma^*)$	4.68	0.00
S_7	$2(n, \pi^*)$	4.79	0.00
S_8	$4(\pi, \pi^*)$	4.88	0.10
S_9	$3(\pi, \sigma^*)$	5.47	0.02
S_{10}	$5(\pi, \pi^*)$	5.48	0.01

State	Character	Excitation energy [eV]
T_1	$1(\pi, \pi^*)$	1.61
T_2	$2(\pi, \pi^*)$	2.49
T_3	$3(\pi, \pi^*)$	2.77
T_4	$4(\pi, \pi^*)$	3.46
T_5	$1(\pi, \sigma^*)$	4.10
T_6	$2(\pi, \sigma^*)$	4.29
T_7	$1(n, \pi^*)$	4.31
T_8	$2(n, \pi^*)$	4.64
T_9	$1(n, \sigma^*)$	4.80
T_{10}	$5(\pi, \pi^*)$	4.85

Table S9c. Vertical excitation energies [eV] for singlets and triplets of I-BODIPY at TD-DFT(TDA)/BHLYP-D3/x2c-TZVPPall level in optimized T_2 geometry

State	Character	Excitation energy [eV]	Oscillator Strength
S_1	1(π, π*)	3.20	0.98
S_2	2(π, π*)	3.74	0.22
S_3	3(π, π*)	4.04	0.12
S_4	1(π, σ*)	4.43	0.00
S_5	2(π, σ*)	4.60	0.00
S_6	1(n, π*)	4.62	0.00
S_7	2(n, π*)	5.00	0.00
S_8	4(π, π*)	5.10	0.05
S_9	1(n, σ*)	5.38	0.00
S_{10}	3(π, σ*)	5.44	0.00

State	Character	Excitation energy [eV]
T_1	1(π, π*)	1.58
T_2	2(π, π*)	2.84
T_3	3(π, π*)	3.06
T_4	4(π, π*)	3.65
T_5	1(π, σ*)	3.97
T_6	2(π, σ*)	4.12
T_7	1(n, σ*)	4.60
T_8	1(n, π*)	4.62
T_9	2(n, σ*)	4.79
T_{10}	5(π, π*)	4.87

Table S10a. Vertical excitation energies [eV] for singlets and triplets of I-BODIPY at sf-X2C-S-TD-DFT/M06-2X/x2c-TZVPPall level in optimized S_0 geometry

State	Character	Excitation energy [eV]	Oscillator Strength
S_1	1(π, π*)	2.88	0.67
S_2	2(π, π*)	3.53	0.11
S_3	3(π, π*)	3.80	0.07
S_4	1(π, σ*)	4.38	0.00
S_5	1(n, π*)	4.49	0.00
S_6	2(π, σ*)	4.54	0.00
S_7	2(n, π*)	4.82	0.00
S_8	4(π, π*)	4.96	0.02
S_9	1(n, σ*)	5.22	0.00
S_{10}	3(π, σ*)	5.52	0.00

State	Character	Excitation energy [eV]
T_1	1(π, π*)	1.49
T_2	2(π, π*)	2.85
T_3	3(π, π*)	3.05
T_4	4(π, π*)	3.78
T_5	1(π, σ*)	4.01
T_6	2(π, σ*)	4.19
T_7	1(n, π*)	4.47
T_8	1(n, σ*)	4.79
T_9	2(n, σ*)	4.80
T_{10}	2(n, π*)	5.02

Table S10b. Vertical excitation energies [eV] for singlets and triplets of I-BODIPY at sf-X2C-S-TD-DFT/M06-2X/x2c-TZVPPall level in optimized S_1 geometry

State	Character	Excitation energy [eV]	Oscillator Strength
S_1	1(π, π*)	2.68	0.34
S_2	2(π, π*)	3.05	0.35
S_3	3(π, π*)	3.63	0.06
S_4	1(n, π*)	4.20	0.00
S_5	1(π, σ*)	4.46	0.00
S_6	2(n, π*)	4.47	0.00
S_7	1(π, σ*)	4.67	0.00
S_8	4(π, π*)	4.69	0.05
S_9	2(π, σ*)	5.31	0.01
S_{10}	5(π, π*)	5.38	0.00

State	Character	Excitation energy [eV]
T_1	1(π, π*)	1.49
T_2	2(π, π*)	2.42
T_3	3(π, π*)	2.74
T_4	4(π, π*)	3.52
T_5	1(π, σ*)	4.07
T_6	1(n, π*)	4.18
T_7	2(π, σ*)	4.28
T_8	2(n, π*)	4.44
T_9	1(n, σ*)	4.76
T_{10}	5(π, π*)	4.85

Table S10c. Vertical excitation energies [eV] for singlets and triplets of I-BODIPY at sf-X2C-S-TD-DFT/M06-2X/x2c-TZVPPall level in optimized T_2 geometry

State	Character	Excitation energy [eV]	Oscillator Strength
S_1	1(π, π*)	2.84	0.66
S_2	2(π, π*)	3.51	0.12
S_3	3(π, π*)	3.76	0.07
S_4	1(π, σ*)	4.29	0.00
S_5	2(π, σ*)	4.45	0.00
S_6	1(n, π*)	4.47	0.00
S_7	2(n, π*)	4.80	0.00
S_8	4(π, π*)	4.91	0.02
S_9	1(n, σ*)	5.14	0.00
S_{10}	3(π, σ*)	5.30	0.00

State	Character	Excitation energy [eV]
T_1	1(π, π*)	1.46
T_2	2(π, π*)	2.81
T_3	3(π, π*)	3.02
T_4	4(π, π*)	3.73
T_5	1(π, σ*)	3.93
T_6	2(π, σ*)	4.09
T_7	1(n, π*)	4.45
T_8	1(n, σ*)	4.51
T_9	2(n, σ*)	4.70
T_{10}	2(n, π*)	4.77

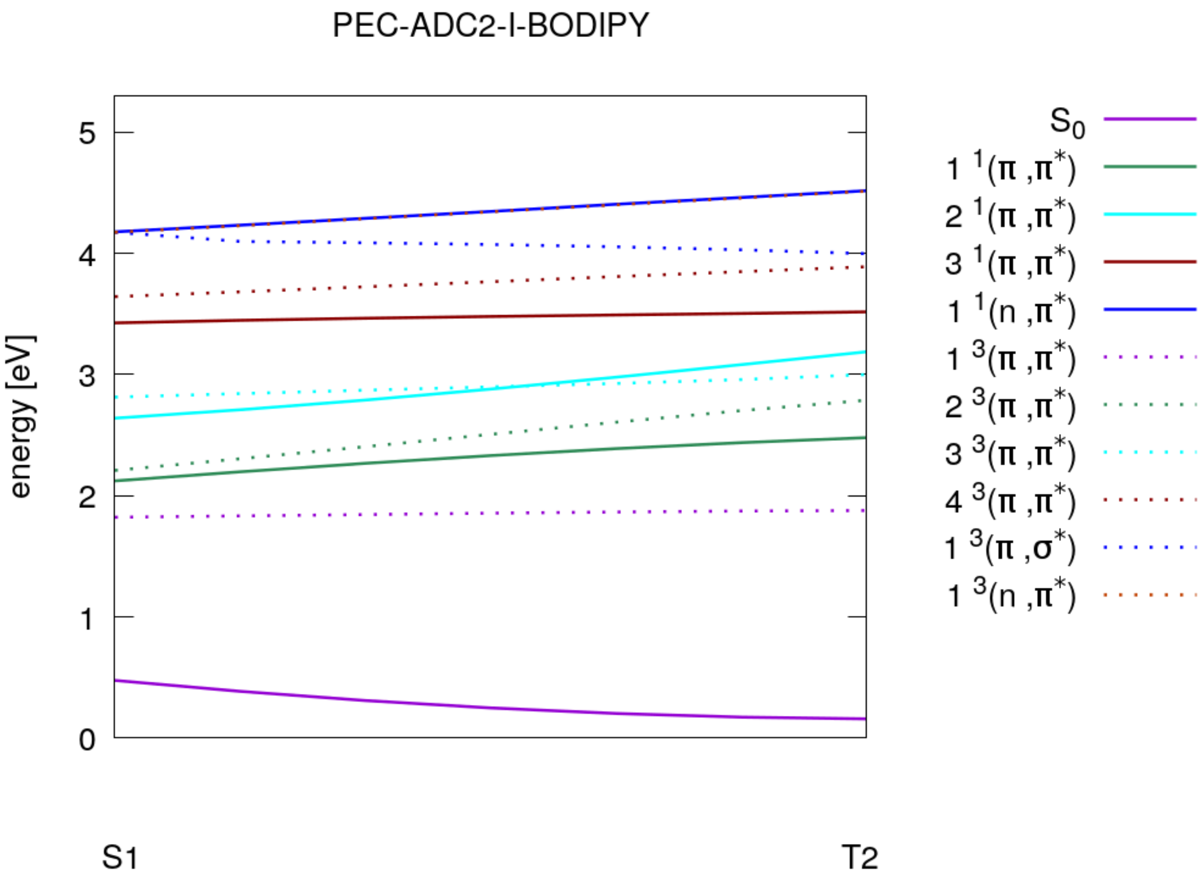

Figure S1. Relative electronic energies for I-BODPY at the ADC(2)/dhf-TZVP level along the PEC with respect to the S_0 energy at S_0 optimized geometry.

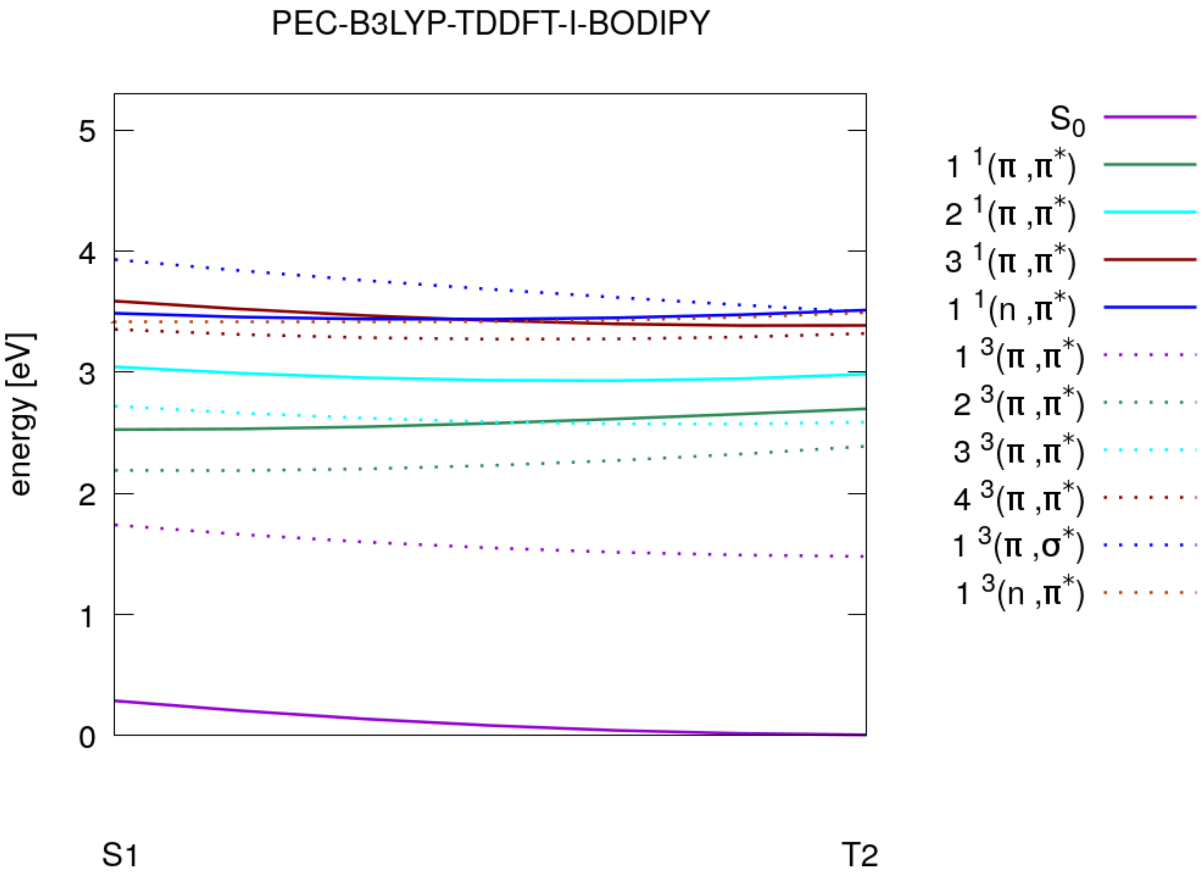

Figure S2. Relative electronic energies for I-BODPY at the TD-DFT/B3LYP/dhf-TZVP level along the PEC with respect to the S_0 energy at S_0 optimized geometry.

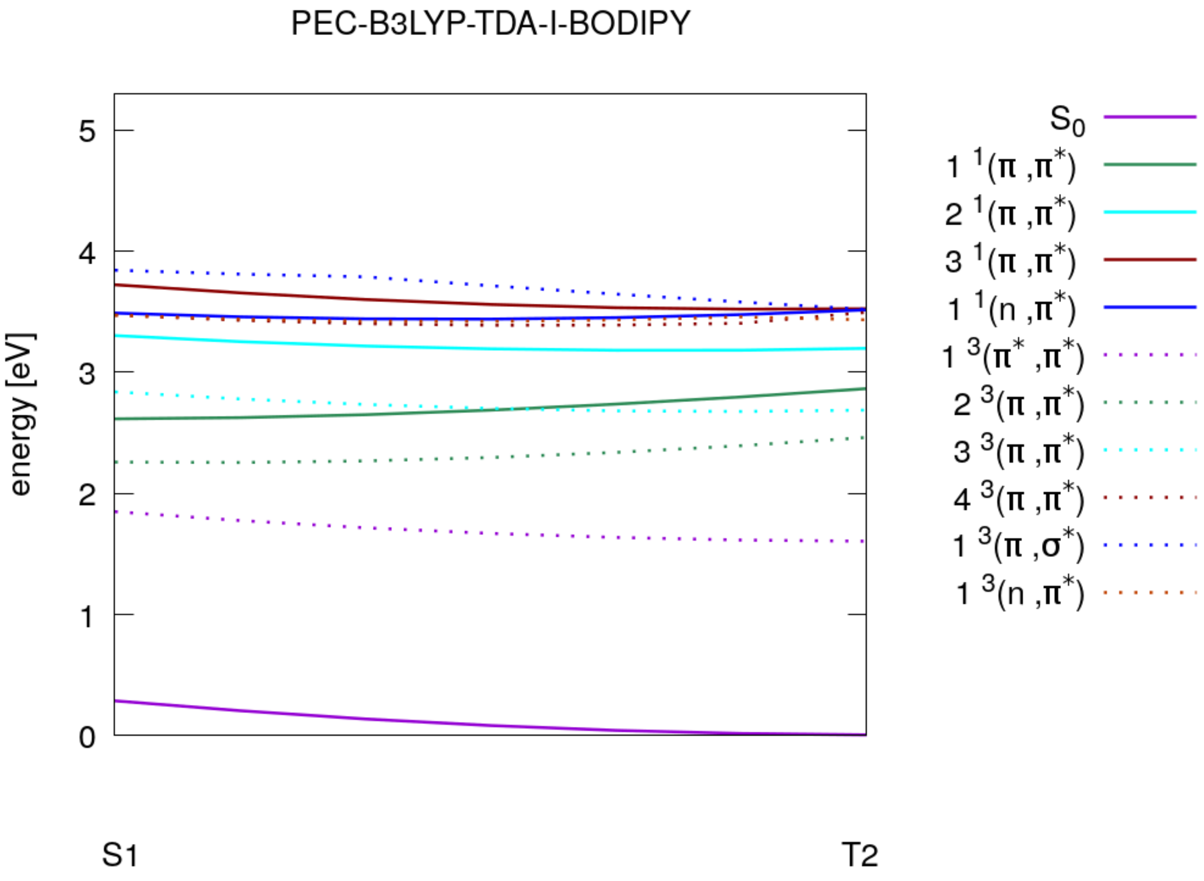

Figure S3. Relative electronic energies for I-BODPY at the TD-DFT(TDA)/B3LYP/dhf-TZVP level along the PEC with respect to the S_0 energy at S_0 optimized geometry.

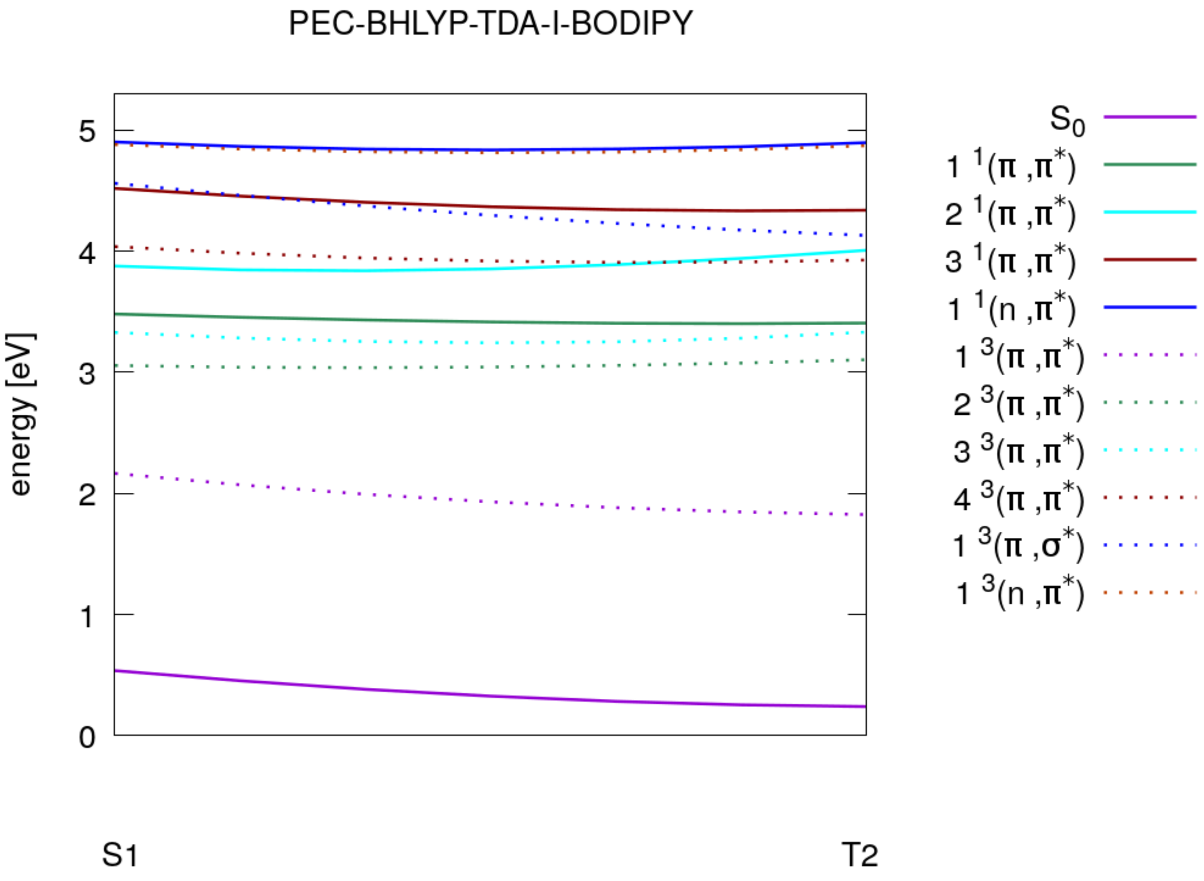

Figure S4. Relative electronic energies for I-BODPY at the TD-DFT(TDA)/BHLYP/dhf-TZVP level along the PEC with respect to the S_0 energy at S_0 optimized geometry.

Table S11. Non-parallelities [eV] between ADC(2) —TD-DFT/B3LYP and ADC(2)—TD-DFT(TDA)/BHLYP for I-BODIPY along the PEC.

State	ADC(2) and TD-DFT/B3LYP	ADC(2) and TD-DFT(TDA)/BHLYP
S_0	0.04	0.02
$1^1(\pi, \pi^*)$	0.19	0.43
$2^1(\pi, \pi^*)$	0.36	0.42
$3^1(\pi, \pi^*)$	0.16	0.27
$1^1(n, \pi^*)$	0.31	0.35
$1^3(\pi, \pi^*)$	0.32	0.33
$2^3(\pi, \pi^*)$	0.38	0.53
$3^3(\pi, \pi^*)$	0.32	0.19
$4^3(\pi, \pi^*)$	0.28	0.36
$1^3(n, \pi^*)$	0.26	0.25
$1^3(\pi, \sigma^*)$	0.27	0.35

Table S12. Shapes and energies [eV] of frontier molecular orbitals of I-BODIPY at DFT/B3LYP/dhf-TZVP level of theory

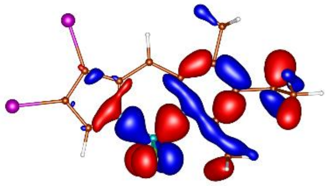	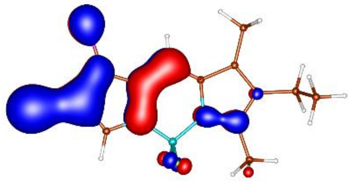
HOMO-8<br>(-9.25)	HOMO-7<br>(-8.71)
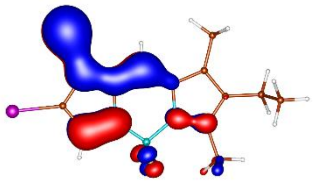	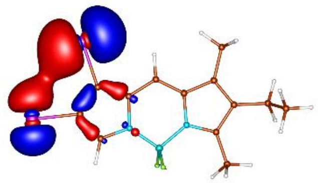
HOMO-6<br>(-8.44)	HOMO-5<br>(-7.67)

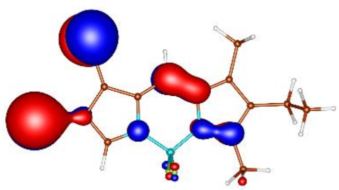	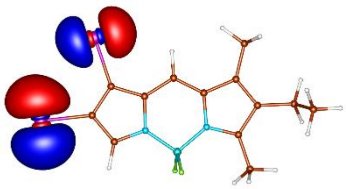
HOMO-4<br>(-7.65)	HOMO-3<br>(-7.20)

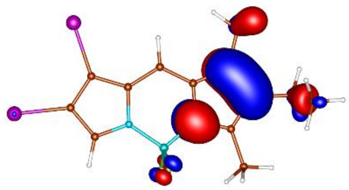	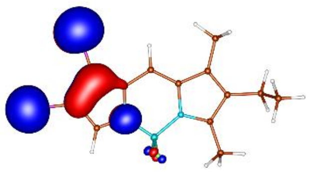
HOMO-2<br>(-7.07)	HOMO-1<br>(-6.53)

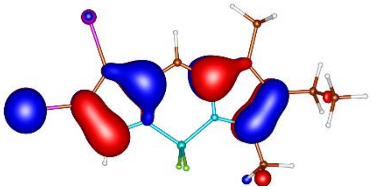
HOMO<br>(-5.98)

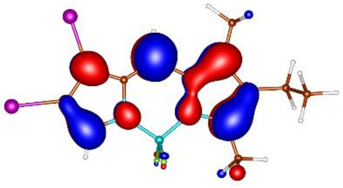
LUMO<br>(-3.07)

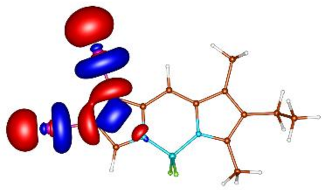	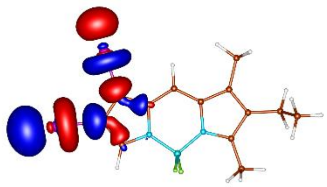
LUMO+1<br>(-1.38)	LUMO+2<br>(-0.55)

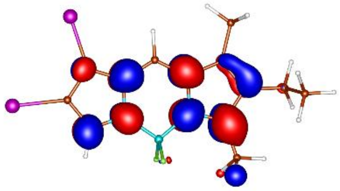	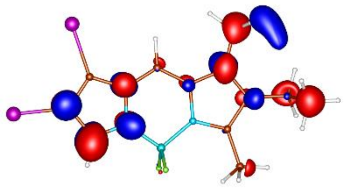
LUMO+3<br>(0.23)	LUMO+4<br>(0.33)

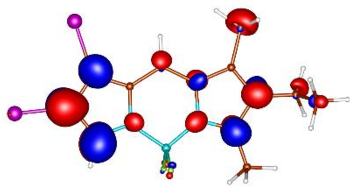
LUMO+5<br>(0.54)

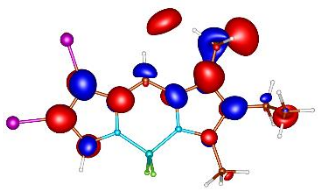	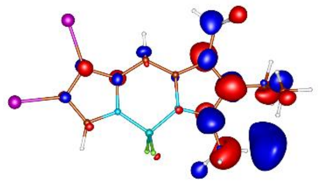
LUMO+6<br>(0.82)	LUMO+7<br>(1.09)

Table S13a. Spin-Orbit Couplings [cm^{-1}] in monoiodinated BODIPY in the position 2 at S_0 optimized geometry at CAS(18,15)SCF/ANO-RCC(TZP) level employing full two-electron Breit--Pauli spin--orbit Hamiltonian

STATE	T_1	T_2	T_3	T_4	T_5	T_6	T_7	T_8	T_9	T_{10}
S_0	0.29	5.05	4.72	4.80	1893	2781	329	28.4	37.2	1121
S_1	1.00	1.37	0.82	0.17	69.1	329	708	1.15	2.09	249
S_2	1.11	0.58	0.96	0.09	30.8	87.3	215	0.71	0.31	106
S_3	2.65	0.03	0.35	0.93	32.7	65.5	703	0.37	2.07	62.6
S_4	1.13	0.74	1.06	0.30	29.1	154	378	1.00	4.08	44.6
S_5	175	96.9	66.5	36.4	2.03	1300	78.4	157	268	4.60
S_6	382	692	645	458	67.7	181	2.87	23.1	20.5	22.4
S_7	3.62	0.86	0.93	1.76	67.8	87.7	1717	0.78	0.64	41.8
S_8	356	162	160	93.3	2.30	1402	55.3	36.4	34.2	3.95
S_9	3.89	1.44	0.22	0.69	6.18	85.0	681	0.35	0.10	115
S_{10}	2.49	2.74	2.92	0.27	16.8	29.8	266	1.06	0.43	84.6

Table S13b. Spin-Orbit Couplings [cm^{-1}] in monoiodinated BODIPY in the position 2 at S_0 optimized geometry at CAS(18,15)SCF/ANO-RCC(TZP) level employing full two-electron DKH1 spin--orbit Hamiltonian

STATE	T_1	T_2	T_3	T_4	T_5	T_6	T_7	T_8	T_9	T_{10}
S_0	0.20	4.27	3.93	3.98	1564	2296	271	23.5	30.7	925
S_1	0.85	1.16	0.62	0.13	57.7	272	584	0.97	1.80	206
S_2	0.92	0.50	0.85	0.04	25.4	72.1	177	0.62	0.30	87.4
S_3	2.28	0.09	0.31	0.78	27.1	54.1	581	0.32	1.75	51.9
S_4	0.91	0.6	0.89	0.25	24.2	127	312	0.84	3.41	36.9
S_5	145	80.2	55.04	30.1	1.67	1074	64.7	130	222	3.80
S_6	315	571	532	378	55.9	151	2.37	19.1	16.9	18.5
S_7	2.99	0.71	0.81	1.49	56.3	72.4	1418	0.65	0.54	34.6
S_8	294	133	133	77.30	1.90	1157	45.7	30.0	28.1	3.27
S_9	3.25	1.22	0.25	0.56	5.02	70.2	562	0.28	0.08	95.7
S_{10}	2.08	2.36	2.47	0.22	13.9	24.6	220	0.90	0.37	70.0

Table S13c. Spin-Orbit Couplings [cm^{-1}] in monoiodinated BODIPY in the position 2 at S_0 optimized geometry at CAS(18,15)SCF/ANO-RCC(TZP) level employing one-electron mean-field DKH1 spin--orbit Hamiltonian

STATE	T_1	T_2	T_3	T_4	T_5	T_6	T_7	T_8	T_9	T_{10}
S_0	0.20	4.29	3.92	3.97	1564	2296	271	23.5	30.7	924
S_1	0.85	1.15	0.61	0.12	57.7	272	584	0.95	1.80	206
S_2	0.91	0.50	0.84	0.05	25.4	72.1	177	0.61	0.30	87.4
S_3	2.31	0.09	0.30	0.78	27.1	54.1	581	0.32	1.76	51.7
S_4	0.89	0.62	0.89	0.25	24.2	127	312	0.84	3.40	36.9
S_5	145	80.3	55.0	30.2	1.67	1074	64.7	130	222	3.81
S_6	315	571	532	378	55.9	150	2.37	19.1	17.0	18.5
S_7	2.98	0.70	0.83	1.50	56.3	72.4	1418	0.65	0.53	34.6
S_8	294	134	133	77.3	1.90	1157	45.7	30.0	28.1	3.27
S_9	3.26	1.24	0.26	0.57	5.03	70.2	562	0.27	0.08	95.7
S_{10}	2.08	2.38	2.47	0.22	14.00	24.6	220	0.90	0.39	70.1

Table S13d. Spin-Orbit Couplings [cm^{-1}] in monoiodinated BODIPY in the position 2 at S_0 optimized geometry at CAS(18,15)SCF/ANO-RCC(TZP) level employing one-electron one-center flexible nuclear screening DKH1 spin--orbit Hamiltonian

STATE	T_1	T_2	T_3	T_4	T_5	T_6	T_7	T_8	T_9	T_{10}
S_0	0.19	4.37	3.92	3.96	1564	2296	271	23.5	30.7	925
S_1	0.85	1.16	0.60	0.11	57.6	272	585	0.99	1.81	206
S_2	0.90	0.50	0.87	0.06	25.4	72.2	177	0.63	0.31	87.4
S_3	2.38	0.09	0.31	0.79	27.1	54.1	581	0.32	1.76	52.0
S_4	0.88	0.62	0.90	0.25	24.1	127	312	0.83	3.41	36.8
S_5	145	80.2	55.0	30.1	1.66	1074	64.8	130	222	3.83
S_6	315	571	533	379	55.9	150	2.38	19.1	17.0	18.5
S_7	2.96	0.69	0.83	1.49	56.2	72.5	1419	0.63	0.53	34.7
S_8	294	134	133	77.2	1.92	1158	45.8	30.0	28.2	3.29
S_9	3.27	1.26	0.28	0.58	5.02	70.2	562	0.29	0.06	95.5
S_{10}	2.08	2.43	2.47	0.22	13.9	24.7	220	0.90	0.41	70.0

Table S14. Spin-Orbit Couplings [cm^{-1}] in monoiodinated BODIPY in the position 2 at S_0 optimized geometry at ADC(2)/dhf-TZVP level

STATE	T_1	T_2	T_3	T_4	T_5	T_6	T_7	T_8	T_9	T_{10}
S_0	1.69	8.29	3.55	11.1	1604	1892	22.7	36.0	5.98	4.54
S_1	0.13	0.42	0.52	0.45	17.8	55.2	0.55	1.56	0.73	0.21
S_2	0.87	1.51	3.01	0.21	74.5	38.0	0.19	0.68	0.36	0.45
S_3	0.40	3.10	2.23	1.70	12.11	23.6	3.16	1.28	0.11	1.36
S_4	413	766	430	596	2.84	172	1717	612	106	183
S_5	53.9	76.7	39.8	32.5	1.36	1781	143	119	141	111
S_6	0.69	2.78	2.26	0.12	103	30.2	2.16	2.65	1.68	0.87
S_7	4.02	10.9	5.41	2.53	1745	23.2	19.2	63.4	59.4	71.9
S_8	2.14	5.91	4.52	2.51	846	52.5	9.82	31.6	30.6	36.7
S_9	42.8	40.4	24.5	5.86	0.31	892	25.5	19.8	4.75	75.4
S_{10}	0.36	0.34	0.28	0.25	35.1	41.4	1.32	0.07	2.16	1.47

Table S15. Spin-Orbit Couplings [cm^{-1}] in monoiodinated BODIPY in the position 2 at S_0 optimized geometry at TD-DFT(TDA)/BHLYP/dhf-TZVP level

STATE	T_1	T_2	T_3	T_4	T_5	T_6	T_7	T_8	T_9	T_{10}
S_0	1.27	8.79	3.39	6.18	1527	234	1886	26.3	33.6	4.3
S_1	0.11	0.55	0.37	0.42	18.2	928	88.4	1.62	1.95	0.91
S_2	0.66	0.38	2.26	0.28	116	1104	74.7	1.15	2.00	0.74
S_3	0.49	2.43	1.20	1.29	14.2	399	21.1	0.37	2.16	0.53
S_4	320	781	392	374	3.16	18.1	987	377	1474	483
S_5	214	515	257	241	3.16	30.9	1405	241	1014	342
S_6	0.43	2.99	1.61	0.21	227	1545	70.7	3.97	7.44	5.14
S_7	3.98	12.3	6.07	2.42	1891	200	8.38	33.5	59.0	56.4
S_8	56.4	50.3	27.4	3.92	0.59	0.85	1072	13.2	50.8	5.12
S_9	0.00	0.69	0.52	0.82	79.1	803	94.0	0.48	0.71	1.89
S_{10}	61.4	158	72.7	74.6	5.03	178	40.3	76.5	296	101

Table S16. Spin-Orbit Couplings [cm^{-1}] in monoiodinated BODIPY in the position 2 at S_0 optimized geometry at TD-DFT/B3LYP/dhf-TZVP level

STATE	T_1	T_2	T_3	T_4	T_5	T_6	T_7	T_8	T_9	T_{10}
S_0	1.93	8.08	0.81	5.12	261	1361	23	1843	66.9	833
S_1	0.36	0.41	1.61	0.65	1476	83.2	0.33	23.3	0.94	15.9
S_2	0.83	1.07	2.67	0.15	962	55.2	1.35	63.8	2.61	146
S_3	583	1248	411	696	1.97	15.5	1723	109	51.9	59.7
S_4	0.34	3.79	2.15	3.75	221	5.54	3.28	20.5	0.63	25.1
S_5	88.8	76.8	14.3	0.97	18.7	0.23	10.9	1425	113	0.61
S_6	1.35	2.84	5.13	0.69	1454	41.7	1.07	55.1	2.72	86.2
S_7	45.8	103	30.9	10.6	14.6	0.64	20.9	1188	45.3	1.06
S_8	3.47	12.4	3.12	7.36	189	1652	25.7	9.05	45.9	1028
S_9	0.38	6.09	0.57	4.17	499	495	10.5	45.9	11.7	306
S_{10}	85.7	188	56.0	106	262	4.72	256	33.8	6.32	7.36

Table S17. Spin-Orbit Couplings [cm^{-1}] in monoiodinated BODIPY in the position 2 at S_0 optimized geometry at TD-DFT/M06-2X/dhf-TZVP level

STATE	T_1	T_2	T_3	T_4	T_5	T_6	T_7	T_8	T_9	T_{10}
S_0	2.04	10.7	4.46	6.95	1541	227	1881	4.72	24.5	8.48
S_1	0.07	0.45	0.25	0.32	18.7	794	62.9	0.17	1.00	0.72
S_2	0.66	0.23	2.71	0.55	94.6	1129	50.4	0.14	1.86	1.79
S_3	0.52	2.66	0.98	1.48	18.1	433	8.47	1.94	0.58	0.77
S_4	393	958	483	494	6.61	1.57	198	1694	878	204
S_5	66.6	113	54.9	38.9	0.93	21.5	1801	168	171	242
S_6	0.20	3.23	1.57	0.46	165	1559	60.7	0.09	9.33	11.2
S_7	8.20	19.9	11.7	0.14	1926	155	9.97	5.92	128	172
S_8	61.2	54.0	28.2	4.69	0.16	4.90	926	39.2	19.5	25.9
S_9	0.04	0.61	0.71	0.81	71.9	938	74.7	0.74	1.29	2.55
S_{10}	71.8	184	86.7	92.5	2.87	104	27.1	319	160	45.5

Table S18. Spin-Orbit Couplings [cm^{-1}] in I-BODIPY at S_0 optimized geometry at ADC(2)/dhf-TZVP level

STATE	T_1	T_2	T_3	T_4	T_5	T_6	T_7	T_8	T_9	T_{10}
S_0	1.64	4.93	2.54	13.5	1556	1431	250	2681	203	500
S_1	0.19	0.48	0.53	1.78	41.6	19.6	504	66.7	465	6.20
S_2	0.89	1.36	2.24	2.07	37.0	29.1	1002	46.5	716	8.26
S_3	0.53	2.46	1.66	1.27	14.5	9.56	265	23.3	217	2.33
S_4	60.2	15.8	17.6	22.4	15.7	2.82	15.6	1018	13.5	42.9
S_5	354	705	624	579	1.81	5.32	1.63	115	98.5	35.5
S_6	63.6	91.8	77.03	41.6	1.42	5.78	28.7	1224	50.8	31.5
S_7	340	477	422	545	18.9	26.9	333	48.3	317	14.8
S_8	110	157	138	180	52.7	79.2	1005	58.4	986	8.93
S_9	49.0	72.5	55.8	21.3	71.8	20.3	31.5	847	6.38	42.5
S_{10}	3.61	15.5	14.3	2.70	45.0	42.6	36.3	53.8	18.3	2.43

Table S19. Spin-Orbit Couplings [cm^{-1}] in I-BODIPY at S_0 optimized geometry at TD-DFT(TDA)/BHLYP/dhf-TZVP level

STATE	T_1	T_2	T_3	T_4	T_5	T_6	T_7	T_8	T_9	T_{10}
S_0	1.74	2.49	3.81	11.7	1544	1326	339	2635	445	25.5
S_1	0.12	0.64	0.70	1.55	46.1	24.6	491	118	25.9	1.99
S_2	0.73	1.31	1.36	1.26	47.9	34.4	1243	70.7	59.0	1.09
S_3	0.63	1.46	1.66	0.88	17.4	21.5	423	28.6	1.03	0.50
S_4	76.0	18.6	33.8	22.6	13.7	3.95	39.1	1057	780	108
S_5	71.0	86.2	158	23.3	1.8	9.19	47.8	1188	1162	8.47
S_6	332	466	898	491	2.28	8.48	6.20	66.9	119	828
S_7	359	339	670	596	8.02	7.09	143	63.9	83.2	801.8
S_8	4.29	6.43	10.3	9.18	97.7	152	1320	135	40.7	15.6
S_9	7.28	2.77	5.10	1.69	1496	1032	116	15.1	12.9	9.41
S_{10}	54.8	56.0	97.7	22.8	86.1	18.0	40.7	795	1015	41.4

Table S20. Spin-Orbit Couplings [cm^{-1}] in I-BODIPY at S_0 optimized geometry at TD-DFT B3LYP/dhf-TZVP level

STATE	T_1	T_2	T_3	T_4	T_5	T_6	T_7	T_8	T_9	T_{10}
S_0	1.89	3.25	1.41	8.29	290	1090	1314	348	16.2	2470
S_1	0.43	1.15	1.61	2.65	1071	86.4	38.7	753	3.14	37.3
S_2	0.88	2.07	1.99	3.87	1121	16.1	104	1079	5.05	43.5
S_3	0.81	2.82	1.32	2.16	392	19.0	12.6	316	2.69	14.9
S_4	432	1077	616	892	3.02	11.1	18.8	124	1241	93.5
S_5	112	42.6	15.1	23.1	0.24	20.0	15.2	1.15	12.5	769
S_6	470	811	452	907	125	4.39	28.3	14.8	1479	50.9
S_7	1.48	8.31	3.24	9.37	1408	58.4	6.92	1463	16.8	98.6
S_8	48.9	139	68.6	22.4	23.9	5.62	8.33	42.9	33.2	1274
S_9	93.5	56.7	20.6	14.9	8.68	96.6	27.7	9.59	22.9	294
S_{10}	4.95	2.87	0.77	5.05	254	1136	1009	311	11.7	42.3

Table S21. Spin-Orbit Couplings [cm^{-1}] in I-BODIPY at S_0 optimized geometry at TD-DFT(TDA)/M06-2X/dhf-TZVP level

STATE	T_1	T_2	T_3	T_4	T_5	T_6	T_7	T_8	T_9	T_{10}
S_0	2.05	4.17	6.87	12.1	1527	1328	321	2605	450	270
S_1	0.13	0.56	0.48	1.98	43.9	31.2	440	82.5	52.4	572
S_2	0.70	1.11	1.82	1.74	53.6	51.0	1253	34.4	71.9	794
S_3	0.66	1.72	1.49	1.19	25.9	22.1	484	17.8	34.0	418
S_4	69.3	23.2	33.0	18.8	10.3	2.56	8.60	1182	802	71.3
S_5	338	573	934	536	8.06	13.4	2.41	293	177	146
S_6	121	55.8	85.9	128	1.15	6.25	22.4	1119	1200	111
S_7	384	408	686	700	13.3	4.01	155	60.2	70.1	40.7
S_8	6.93	9.82	14.5	16.0	82.5	115	1315	102	130	1550
S_9	14.0	6.42	10.1	4.87	1537	1014	105	7.58	25.6	45.2
S_{10}	48.5	61.8	90.9	21.5	86.8	8.55	30.6	840	1050	86.6

Graphs showing variation in magnitudes of SOC in I-BODIPY between selected singlets and triplets along the PEC.

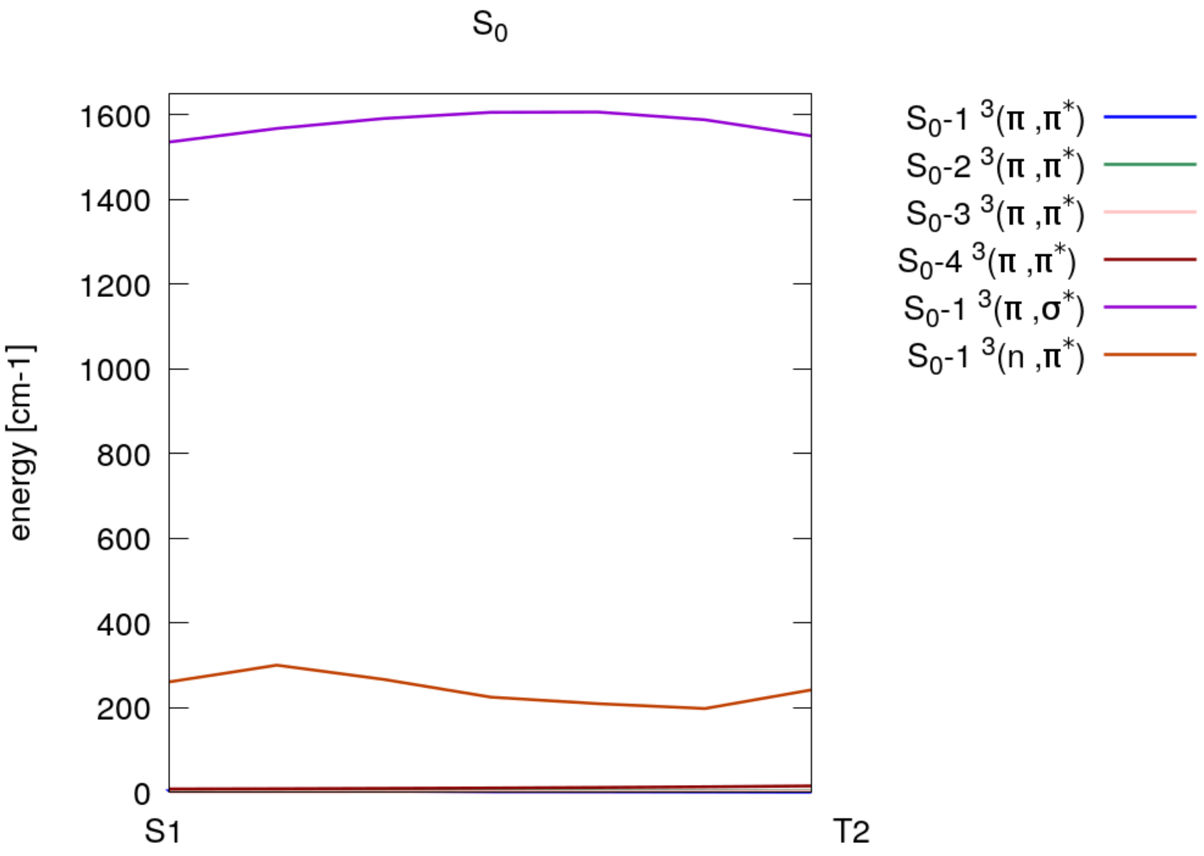

Figure S5. SOCs between ground state S_0 and 6 triplets at ADC(2)/dhf-TZVP level along the PEC.

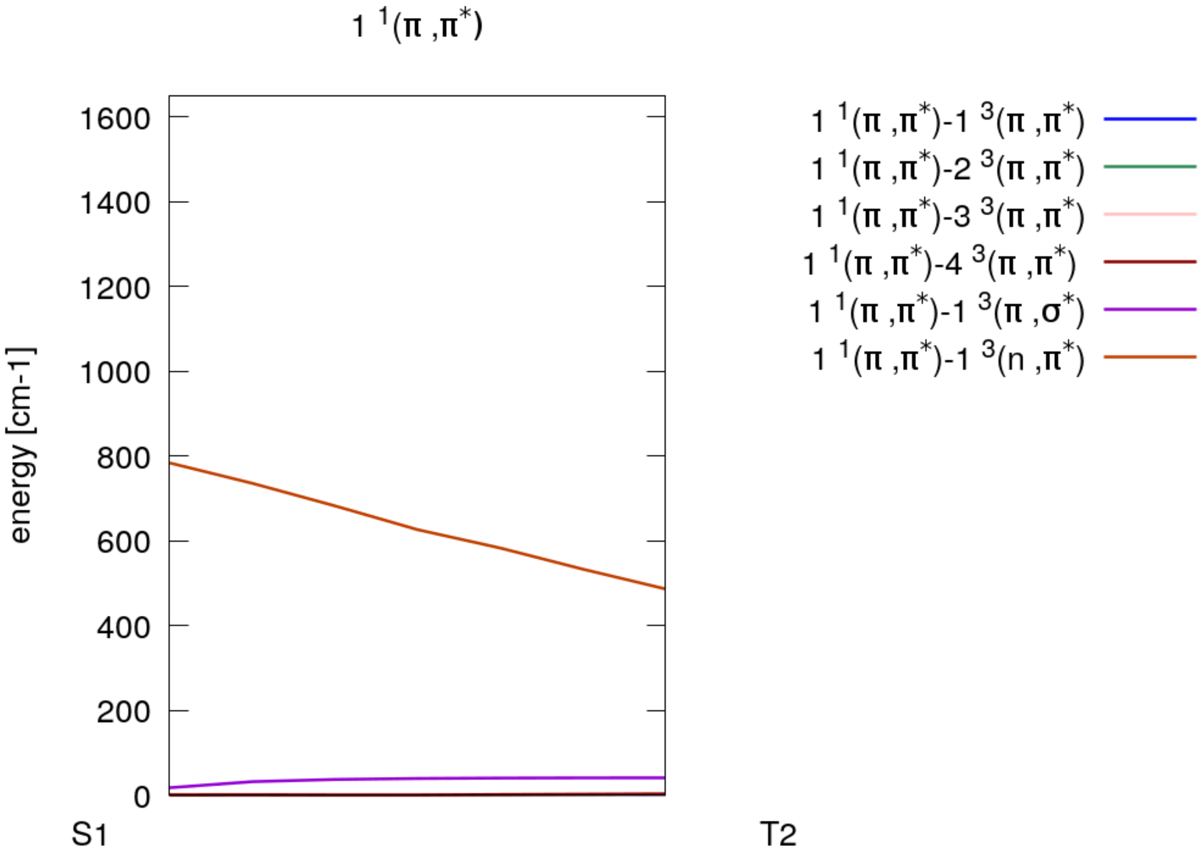

Figure S6. SOCs between excited state 1^1 (π, π^*) and 6 triplets at ADC(2)/dhf-TZVP level along the PEC.

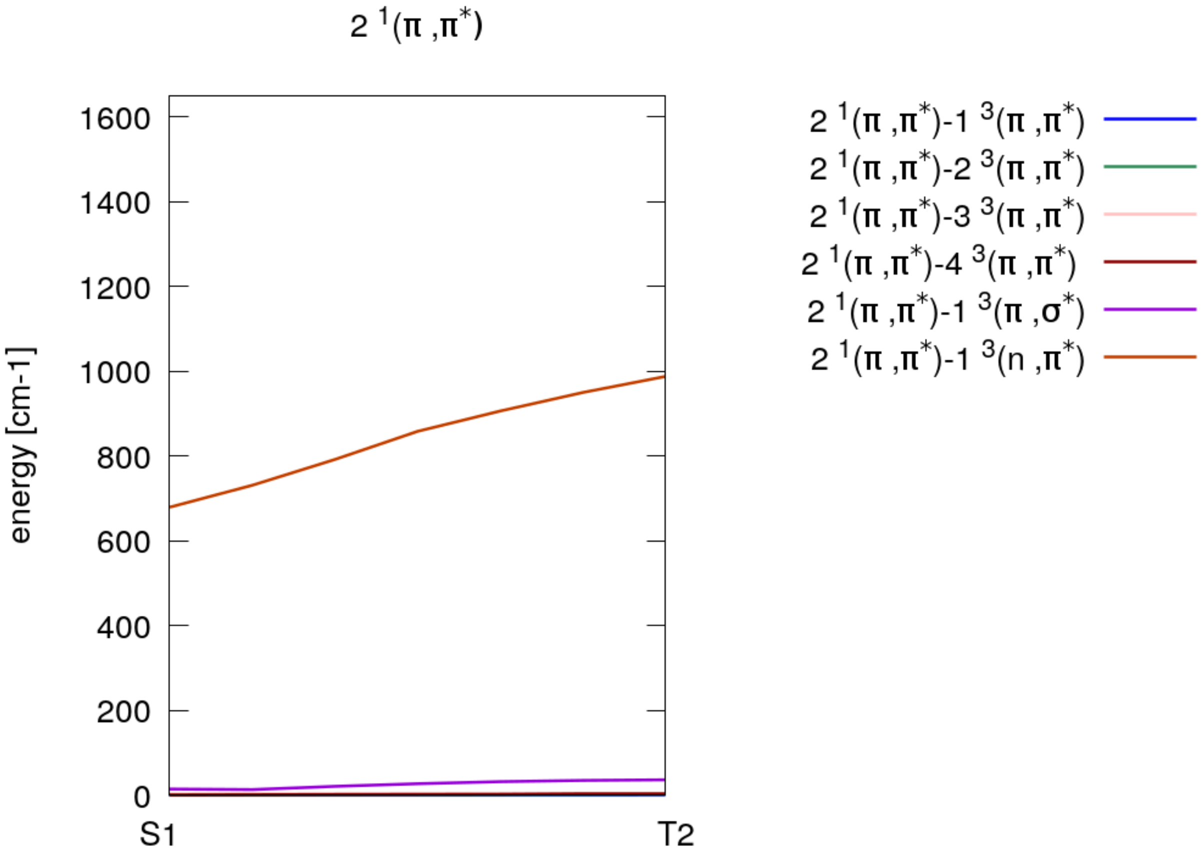

Figure S7. SOCs between excited state 2^1 (π, π*) and 6 triplets at ADC(2)/dhf-TZVP level along the PEC.

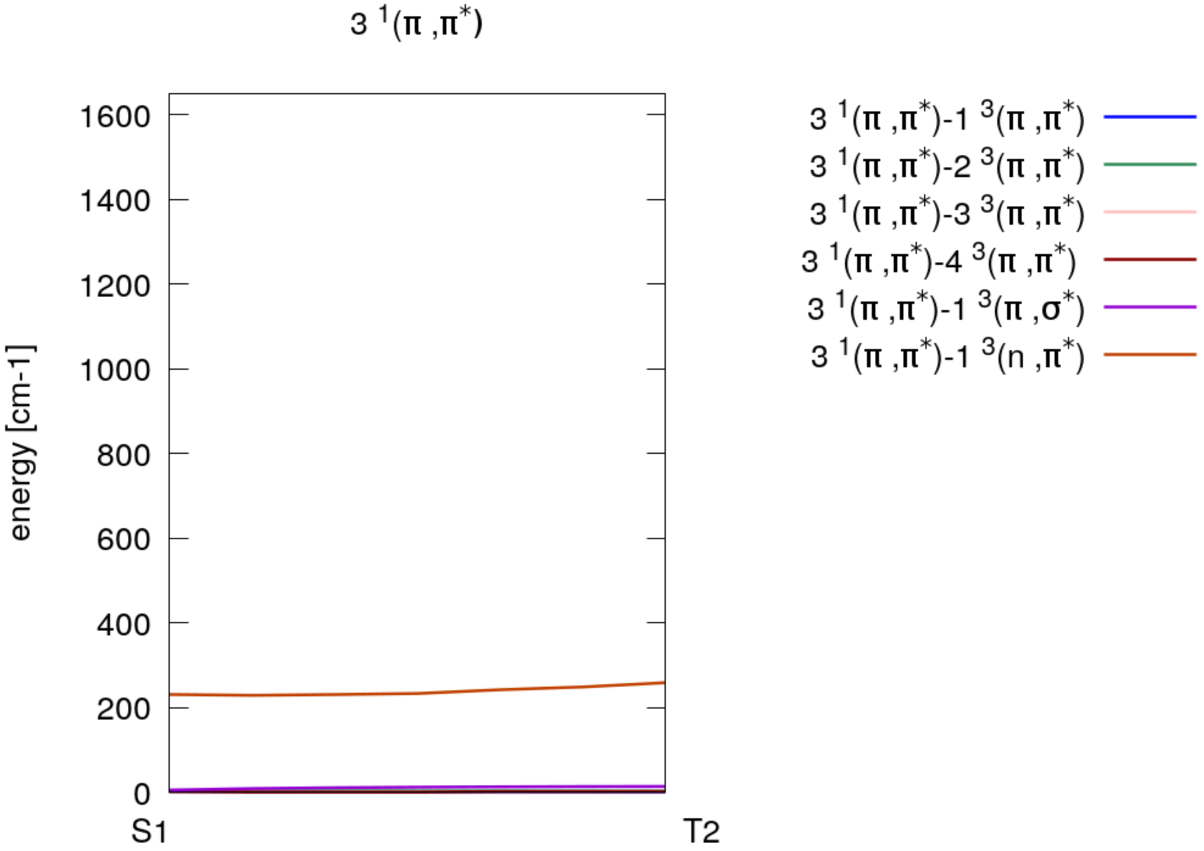

Figure S8. SOCs between excited state 3^1 (π, π*) and 6 triplets at ADC(2)/dhf-TZVP level along the PEC.

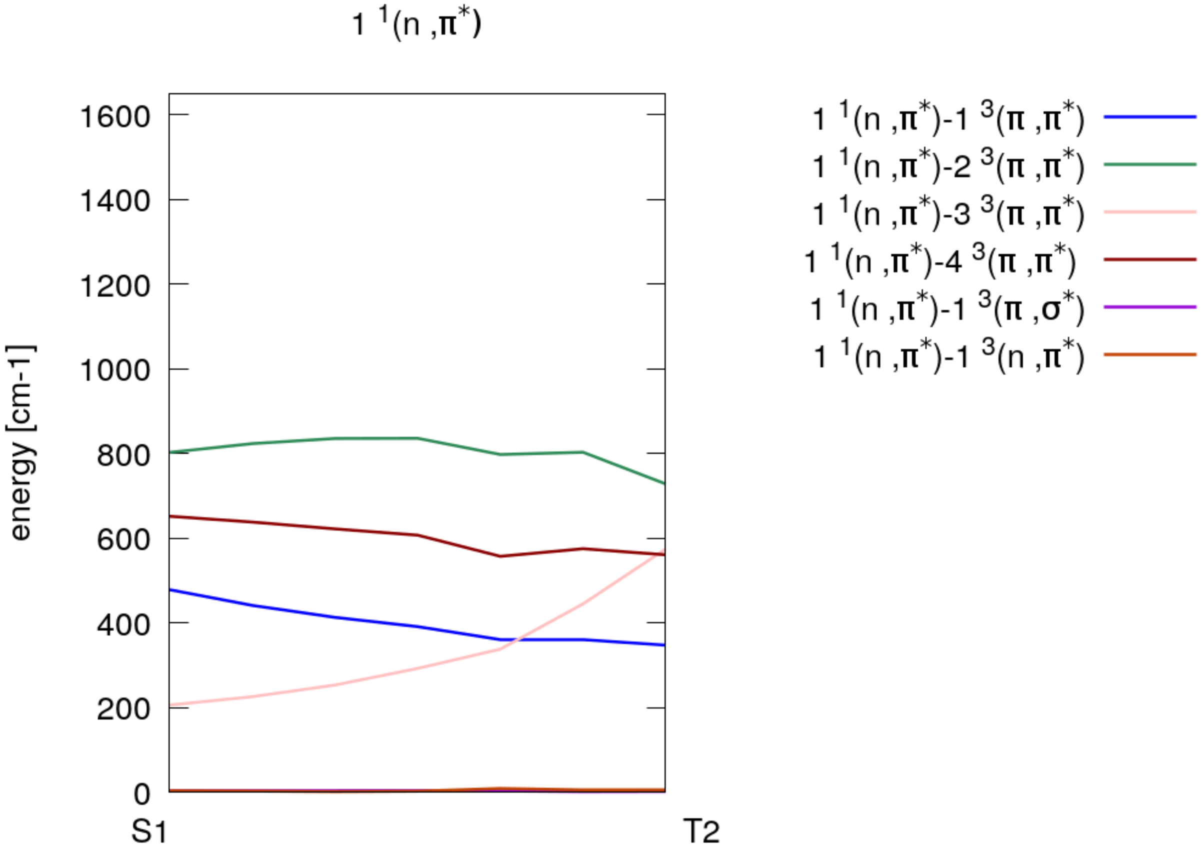

Figure S9. SOCs between excited state 1^1 (n, π*) and 6 triplets at ADC(2)/dhf-TZVP level along the PEC.

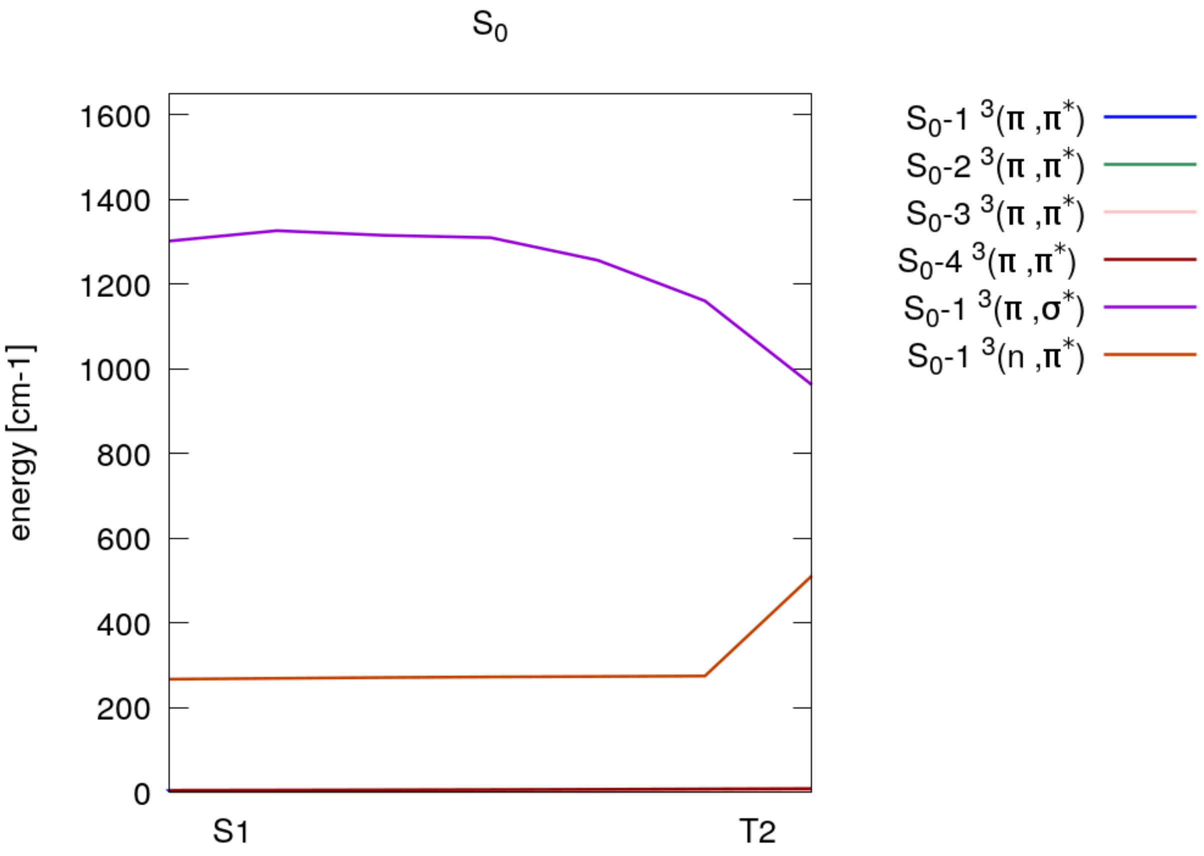

Figure S10. SOCs between ground state S_0 and 6 triplets at TD-DFT/B3LYP/dhf-TZVP level along the PEC.

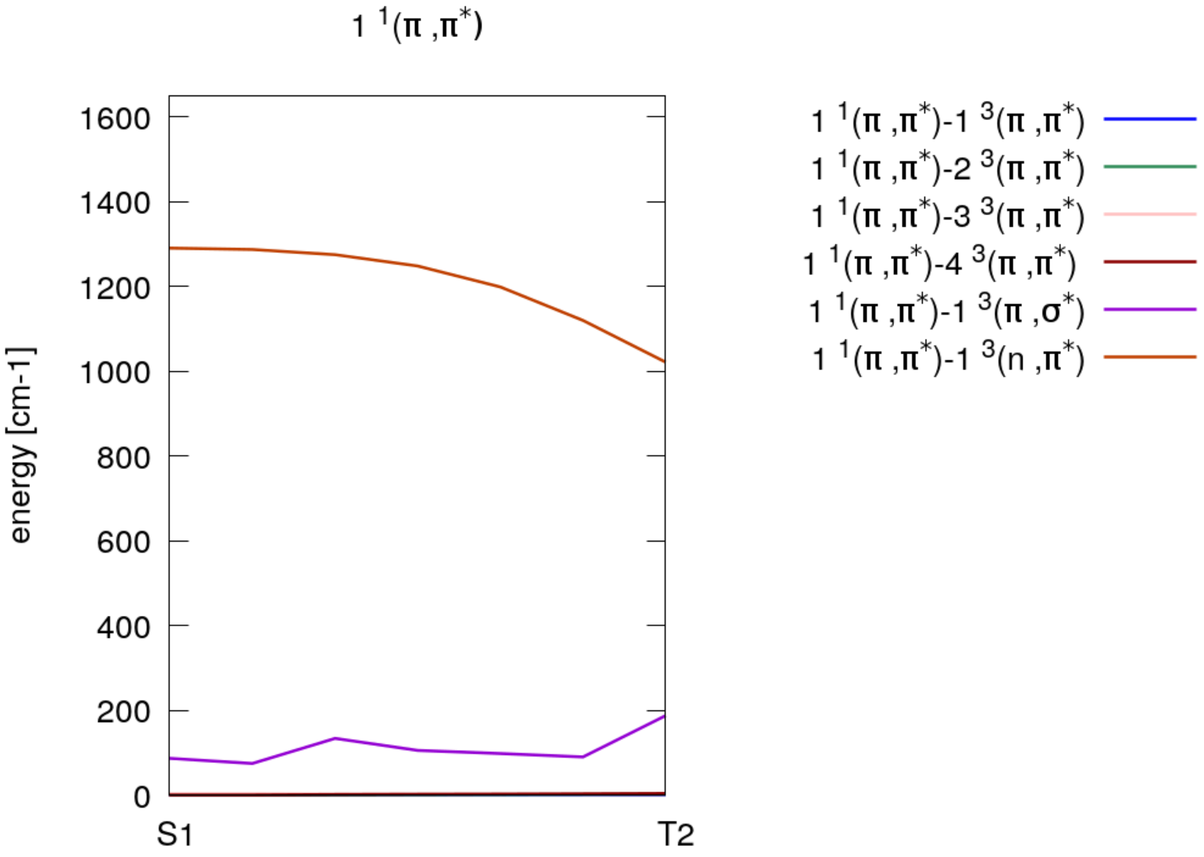

Figure S11. SOCs between excited state 1^1 (π, π*) and 6 triplets at TD-DFT/B3LYP/dhf-TZVP level along the PEC.

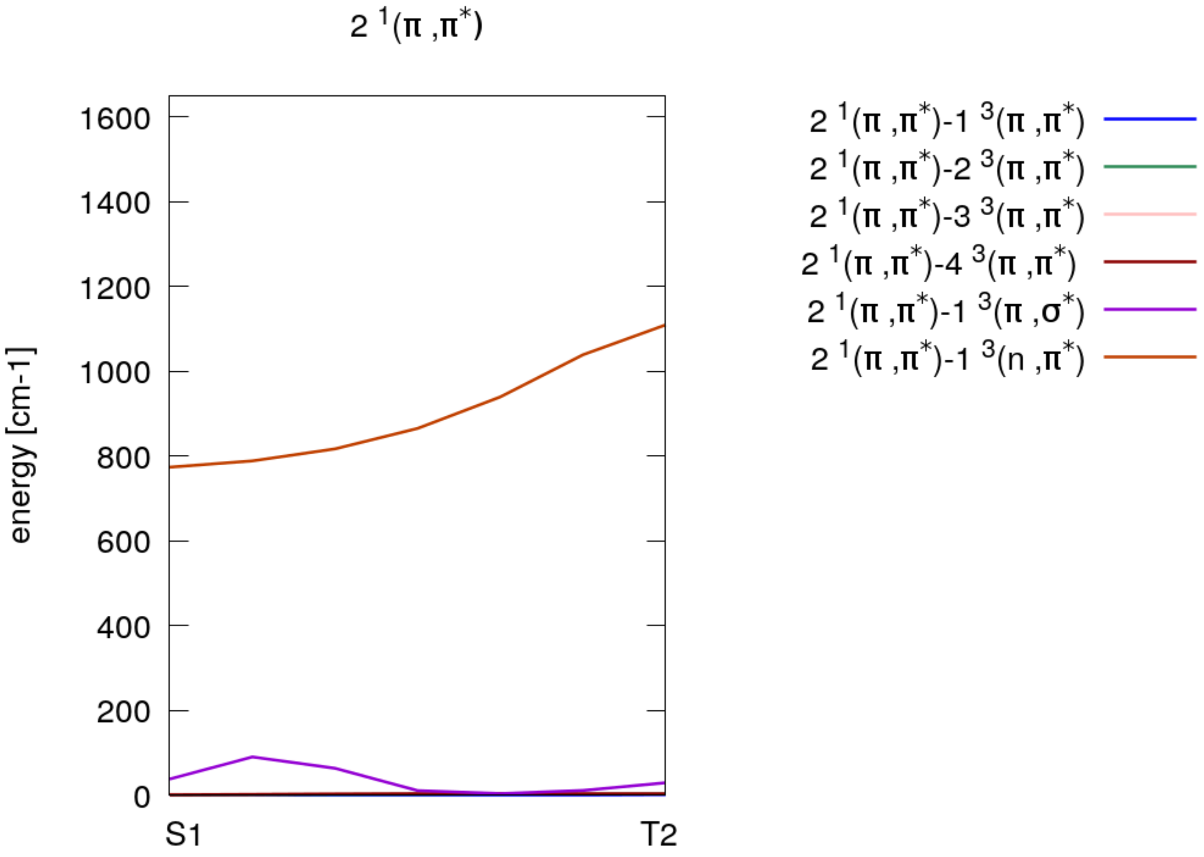

Figure S12. SOCs between excited state 2^1 (π, π*) and 6 triplets at TD-DFT/B3LYP/dhf-TZVP level along the PEC.

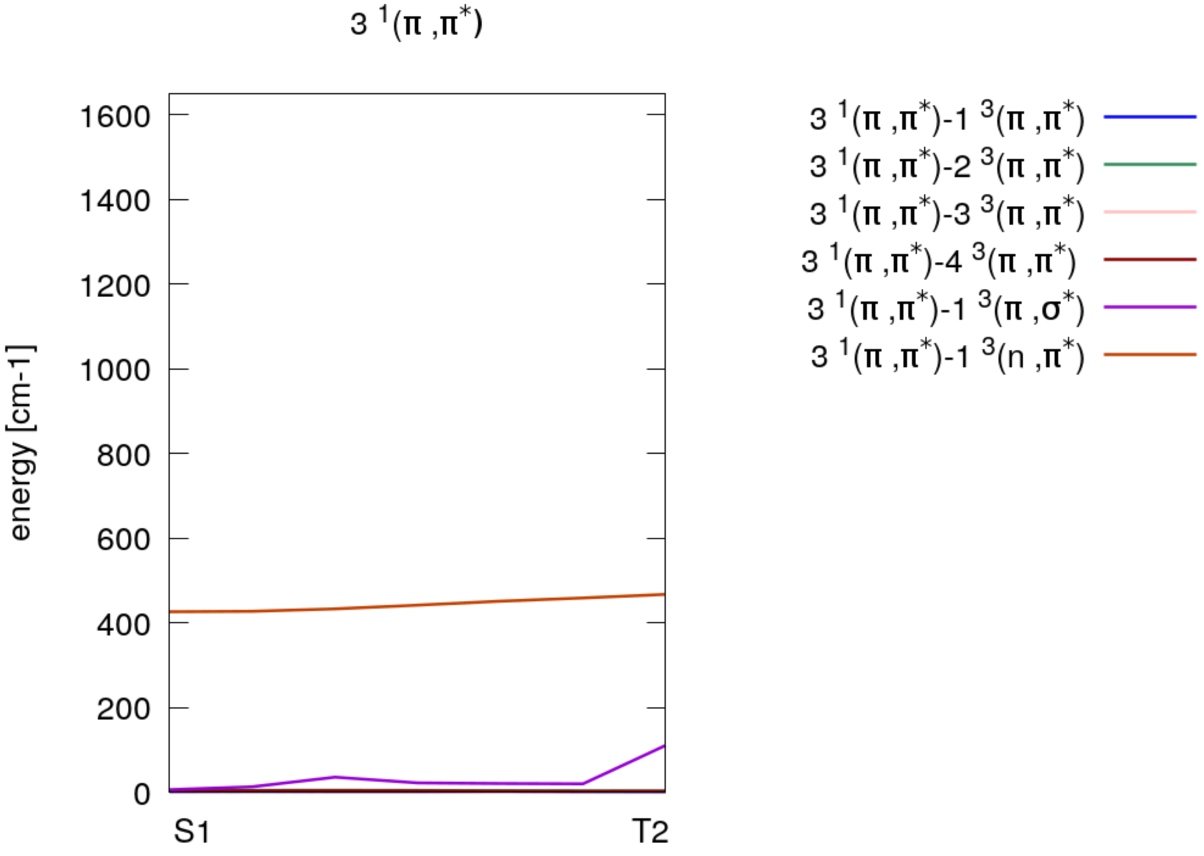

Figure S13. SOCs between excited state 3^1 (π, π*) and 6 triplets at TD-DFT/B3LYP/dhf-TZVP level along the PEC.

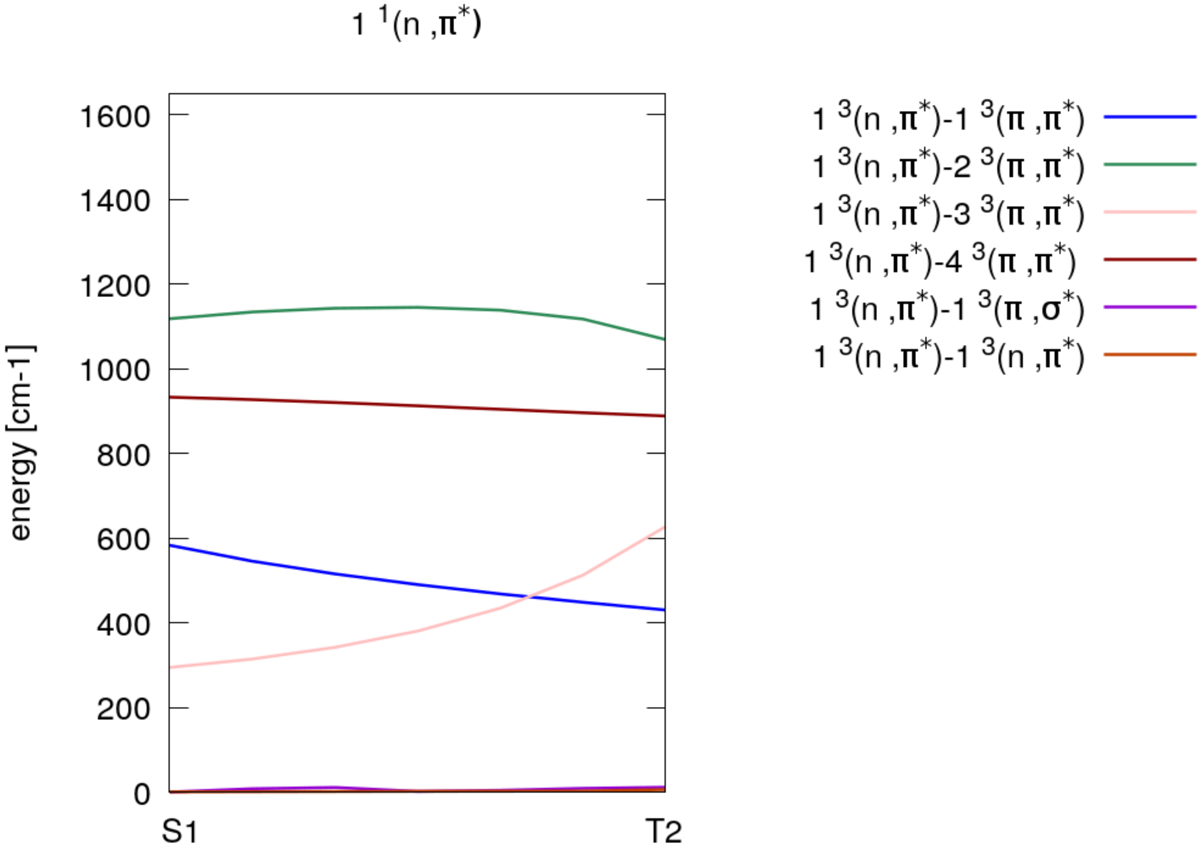

Figure S14. SOCs between excited state 1^1 (n, π*) and 6 triplets at TD-DFT/B3LYP/dhf-TZVP level along the PEC.

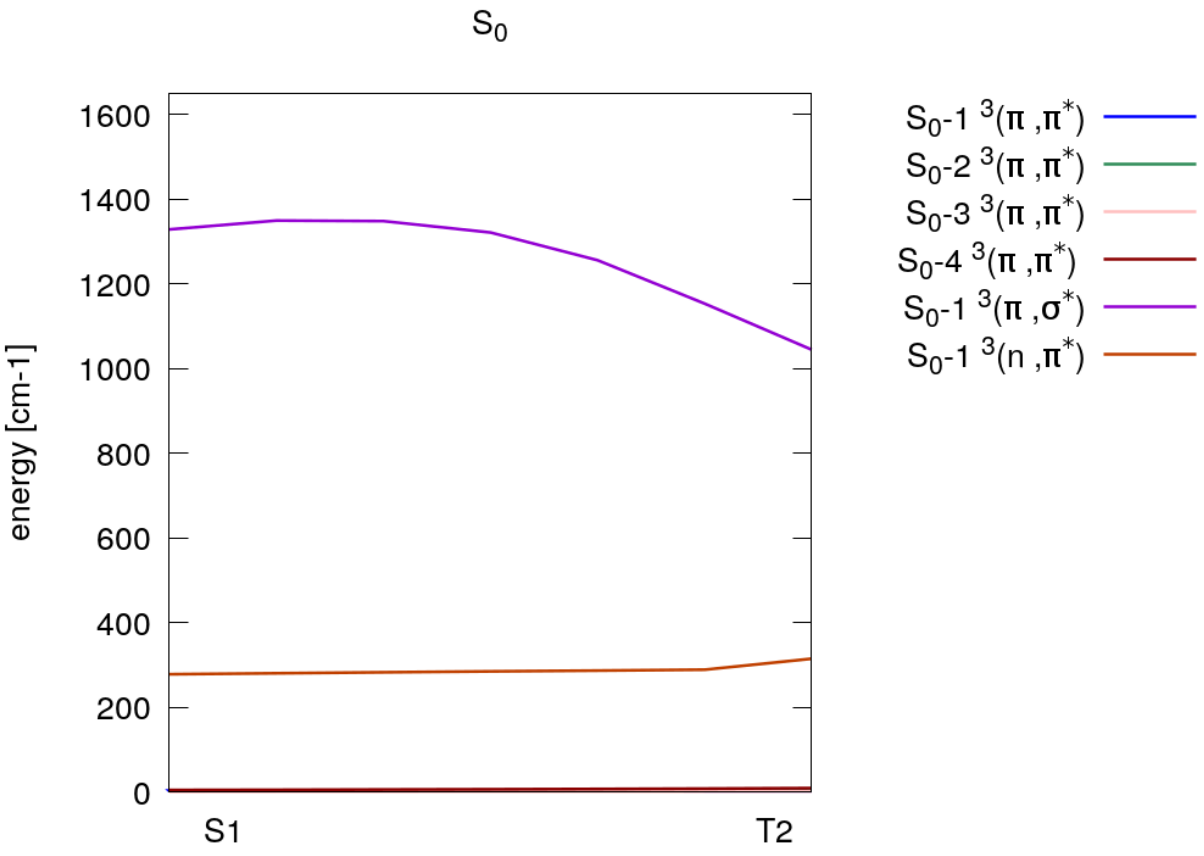

Figure S15. SOCs between ground state S_0 and 6 triplets at TD-DFT(TDA)/B3LYP/dhf-TZVP level along the PEC.

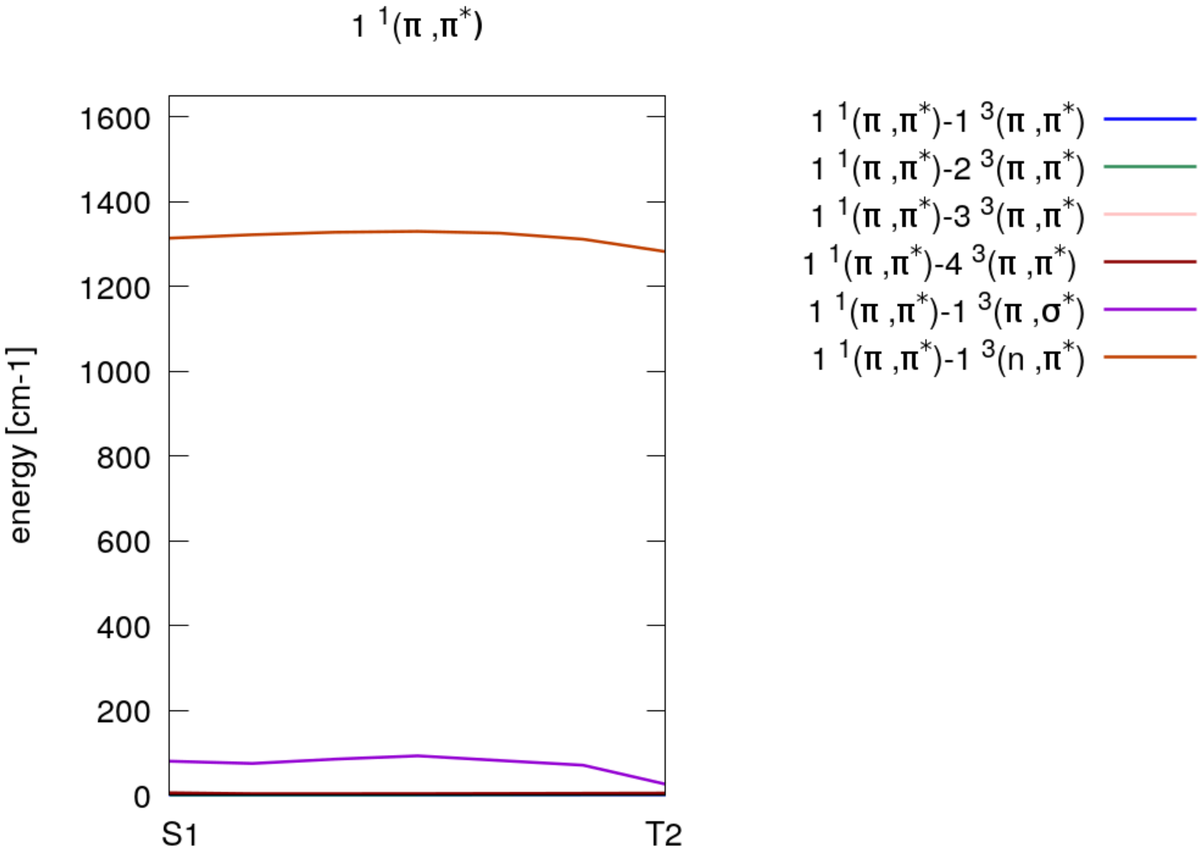

Figure S16. SOCs between excited state 1^1 (π, π*) and 6 triplets at TD-DFT(TDA)/B3LYP/dhf-TZVP level along the PEC.

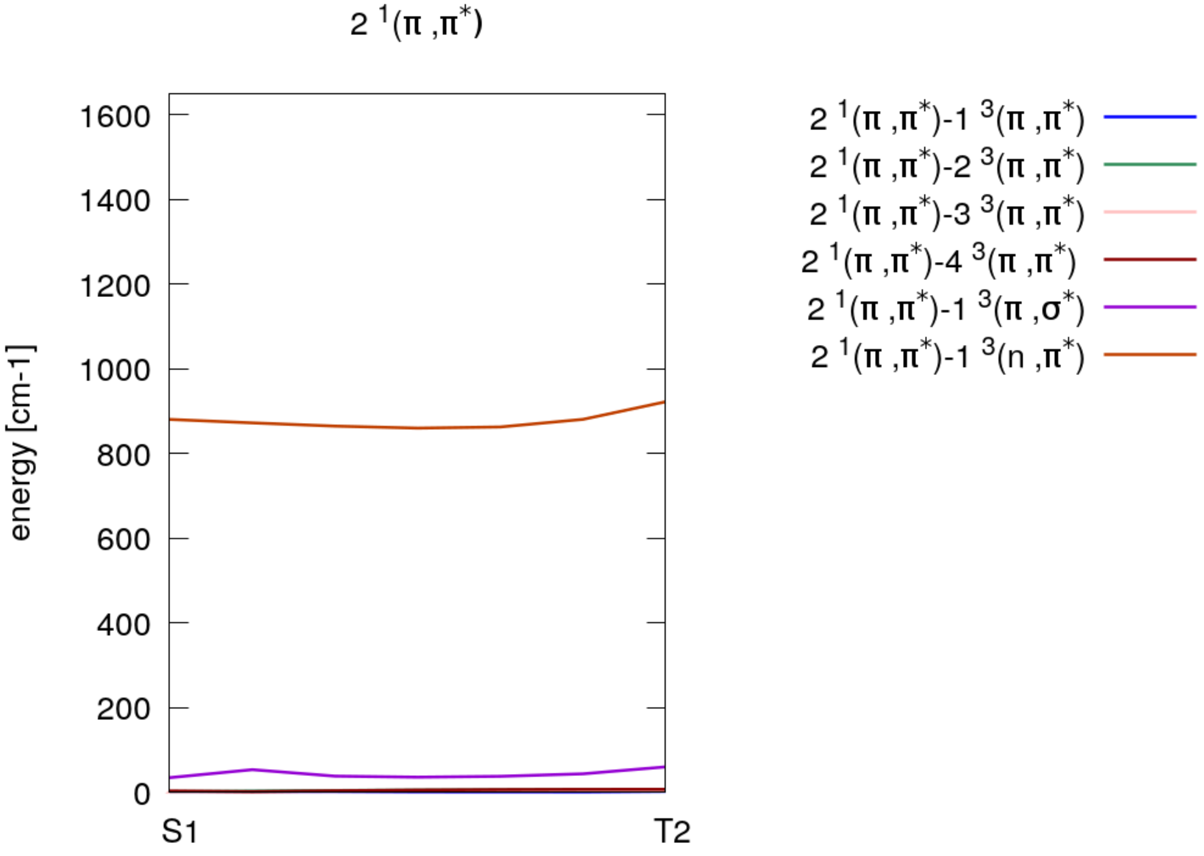

Figure S17. SOCs between excited state 2^1 (π, π*) and 6 triplets at TD-DFT(TDA)/B3LYP/dhf-TZVP level along the PEC.

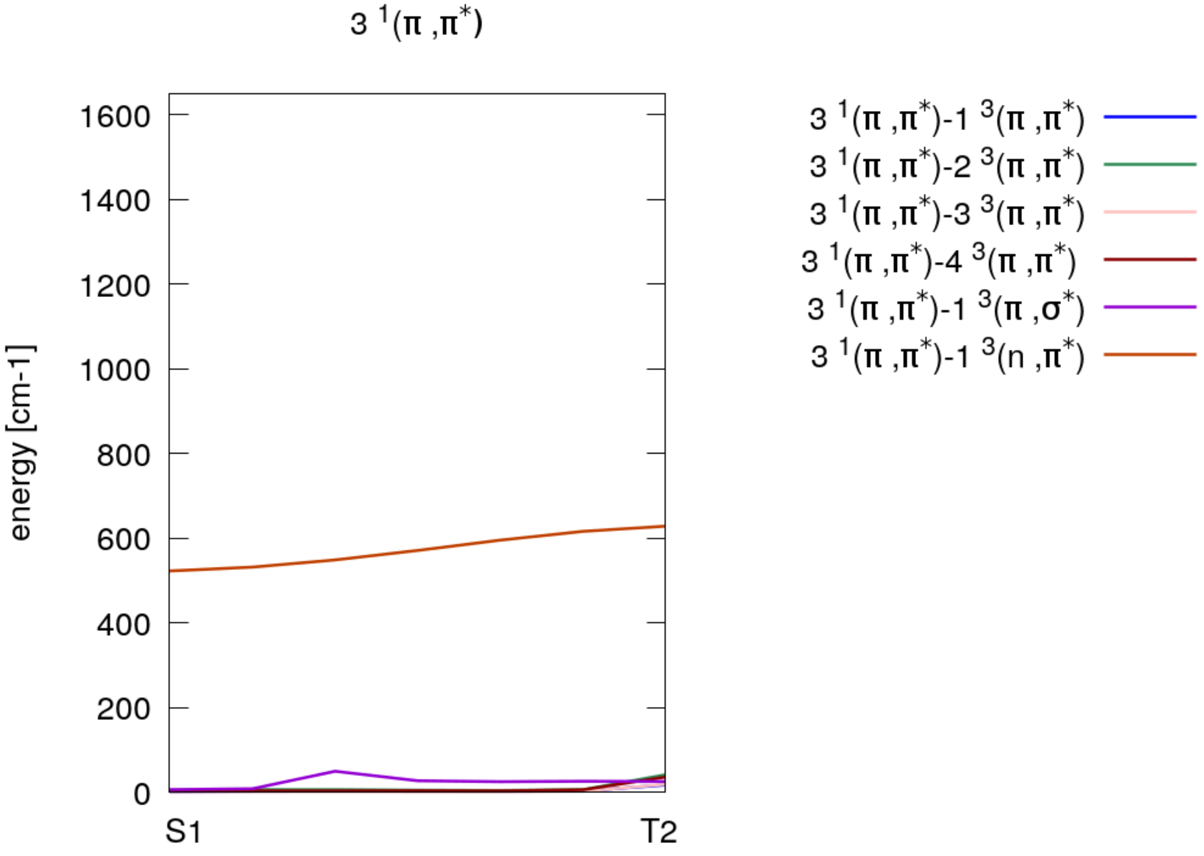

Figure S18. SOCs between excited state 3^1 (π, π*) and 6 triplets at TD-DFT(TDA)/B3LYP/dhf-TZVP level along the PEC.

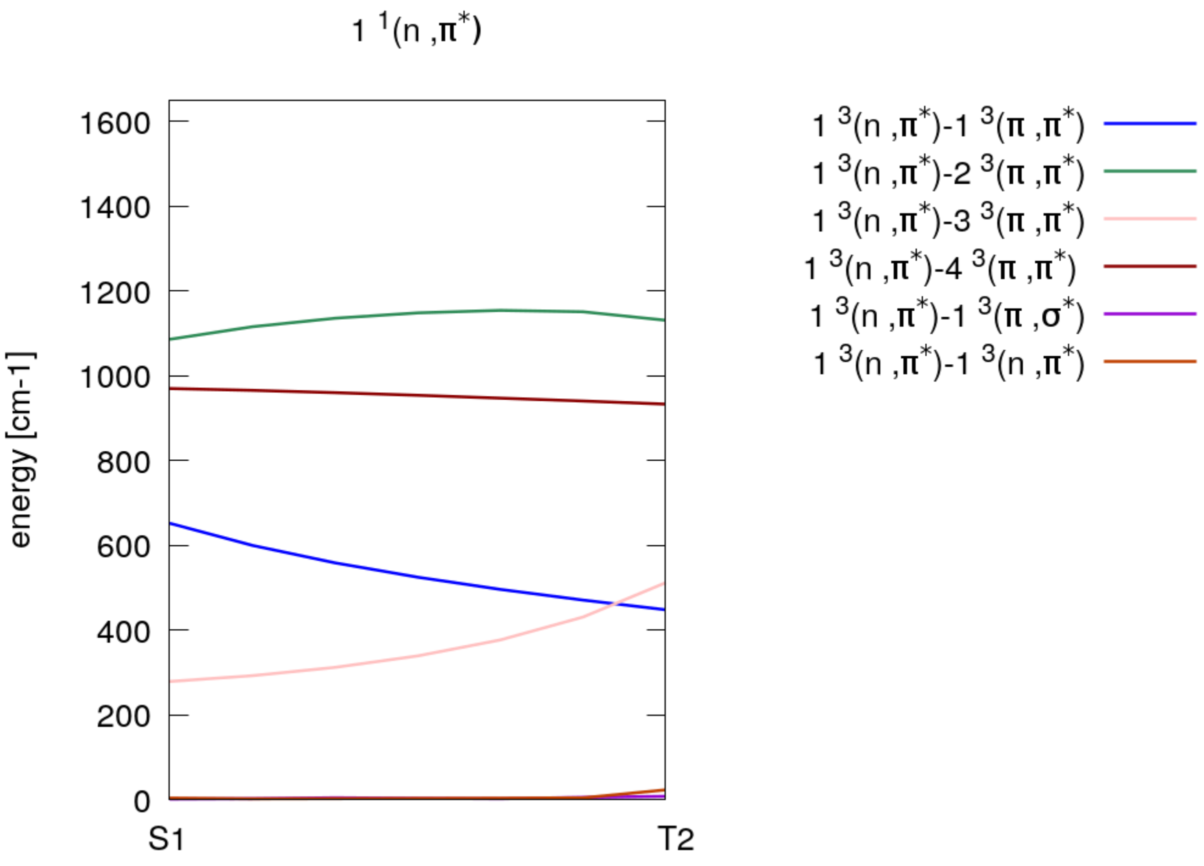

Figure S19. SOCs between excited state 1^1 (n, π*) and 6 triplets at TD-DFT(TDA)/B3LYP/dhf-TZVP level along the PEC.

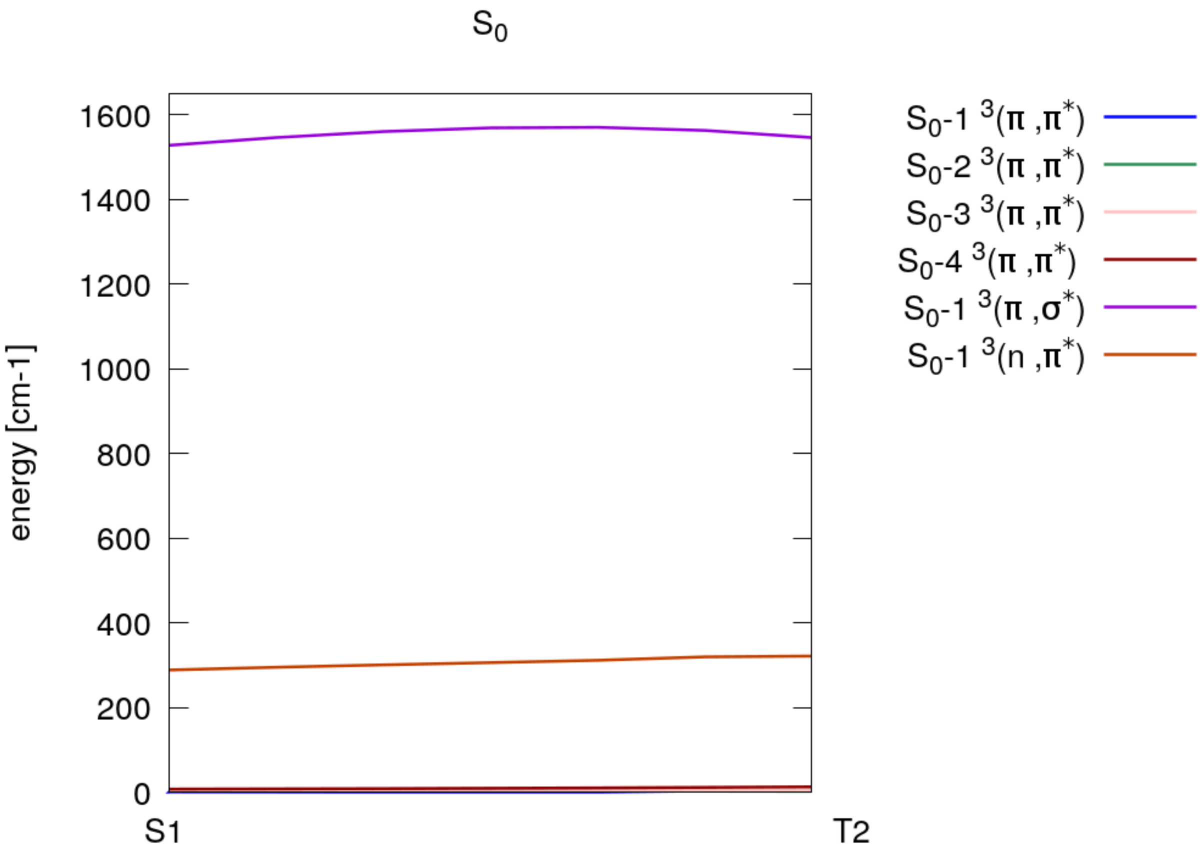

Figure S20. SOCs between ground state S_0 and 6 triplets at TD-DFT/BHLYP/dhf-TZVP level along the PEC.

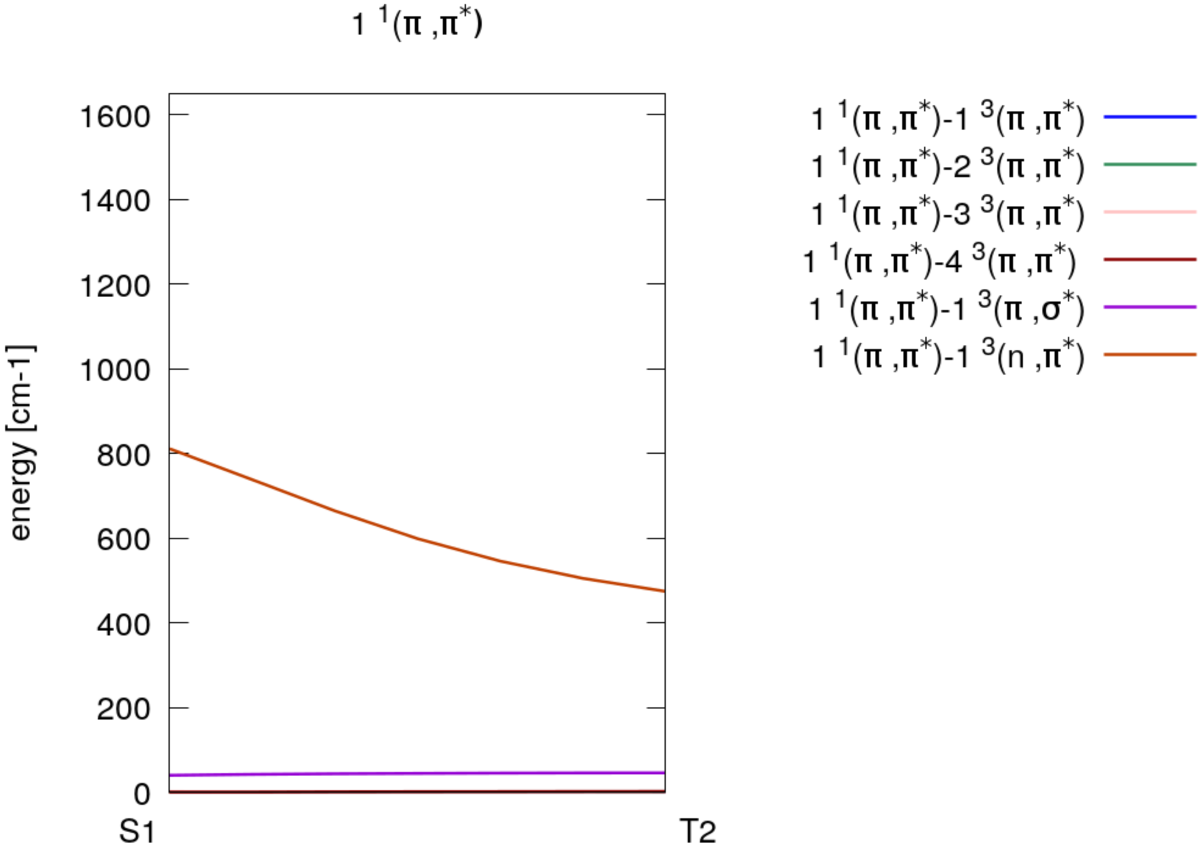

Figure S21. SOCs between excited state 1^1 (π, π*) and 6 triplets at TD-DFT/BHLYP/dhf-TZVP level along the PEC.

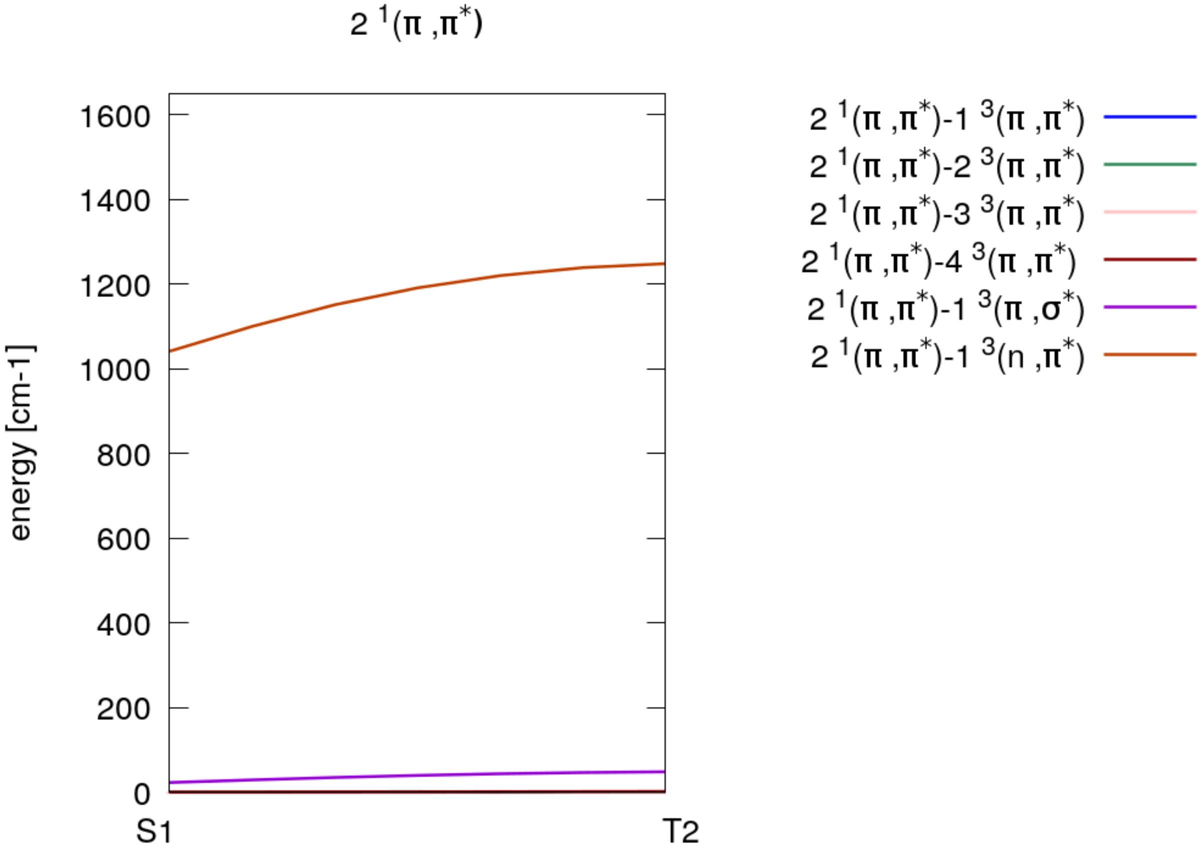

Figure S22. SOCs between excited state 2^1 (π, π*) and 6 triplets at TD-DFT(TDA)/BHLYP/dhf-TZVP level along the PEC.

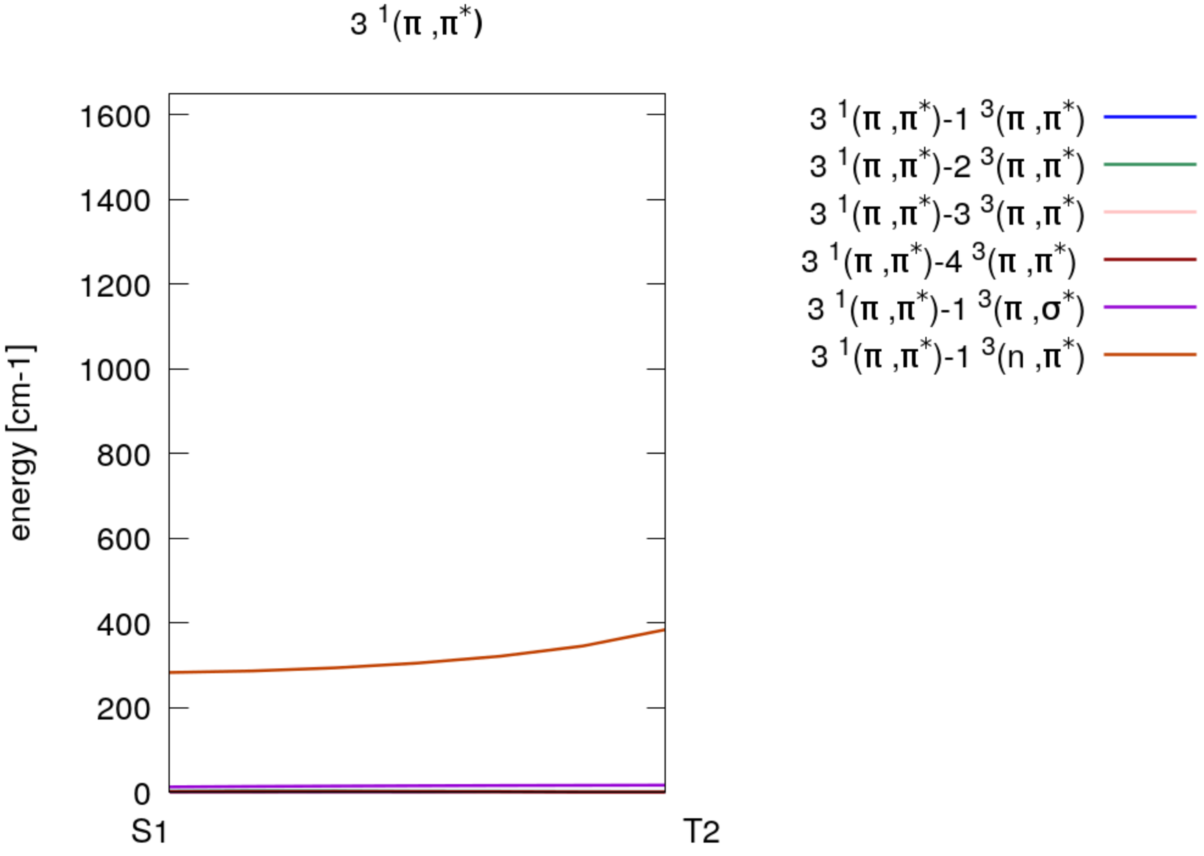

Figure S23. SOCs between excited state 3^1 (π, π*) and 6 triplets at TD-DFT(TDA)/BHLYP/dhf-TZVP level along the PEC.

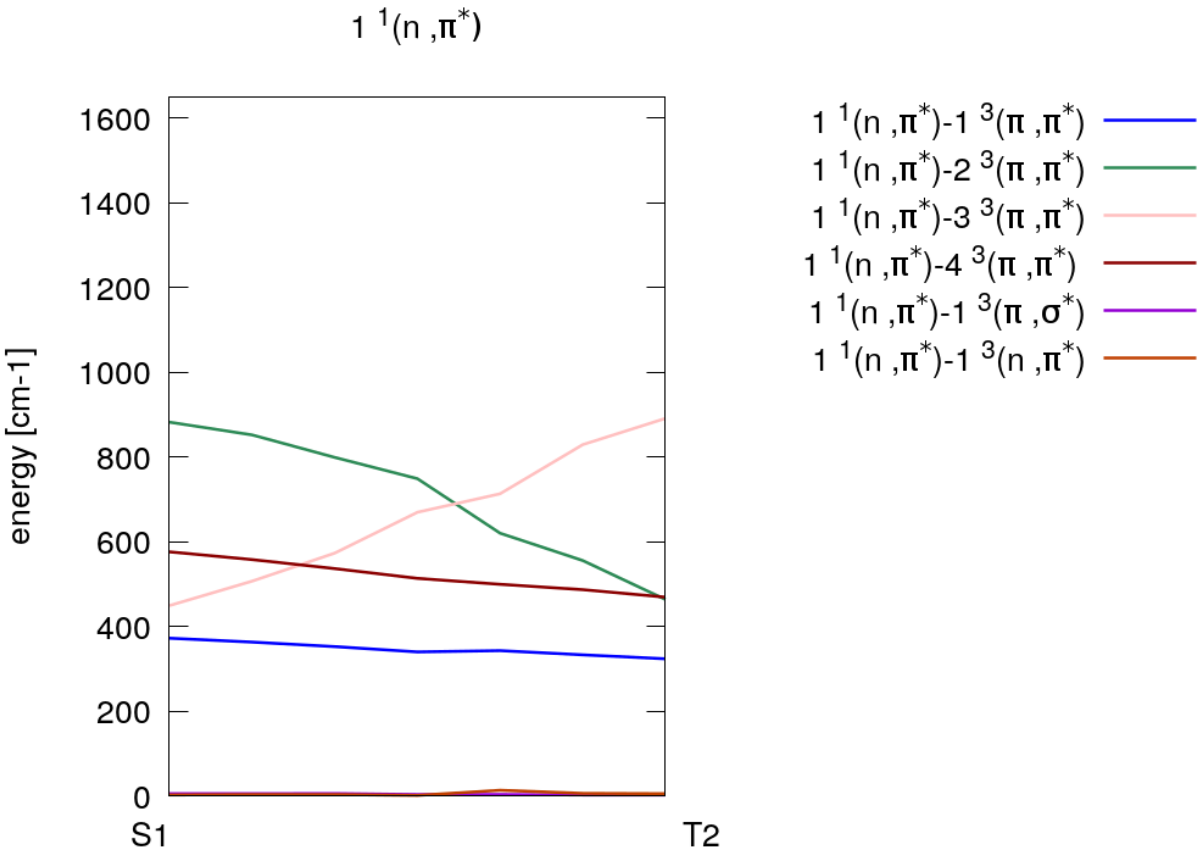

Figure S24. SOCs between excited state 1^1 (n, π*) and 6 triplets at TD-DFT(TDA)/BHLYP/dhf-TZVP level along the PEC.

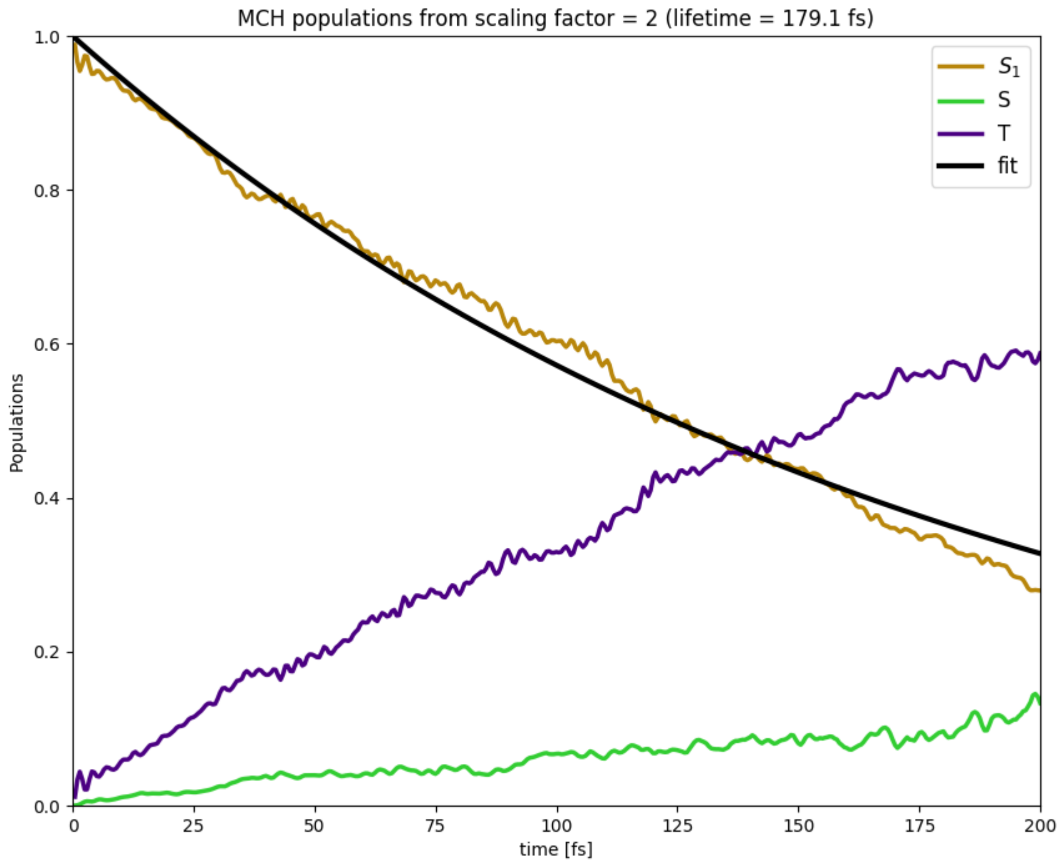

Figure S25. Time evolution of populations of singlets and triplets in I-BODIPY for excited state dynamics started in excited state S_1 using scaling factor 2

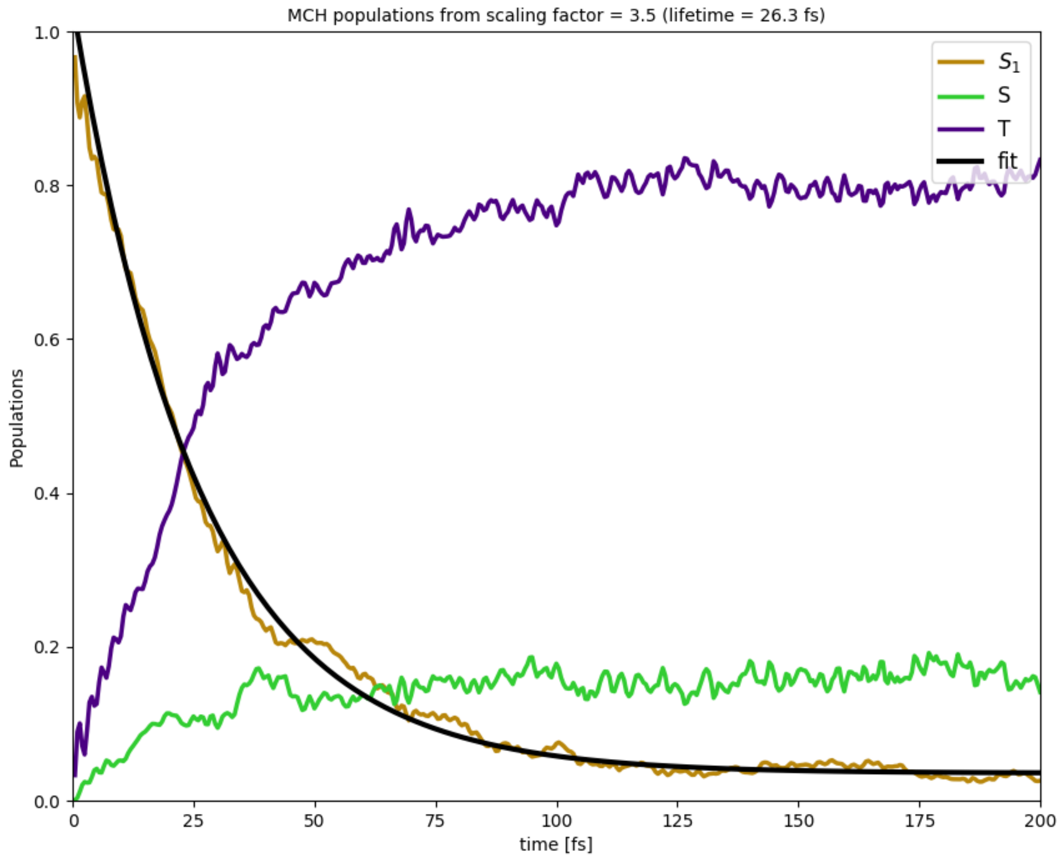

Figure S26. Time evolution of populations of singlets and triplets in I-BODIPY for excited state dynamics started in excited state S_1 using scaling factor 3.5

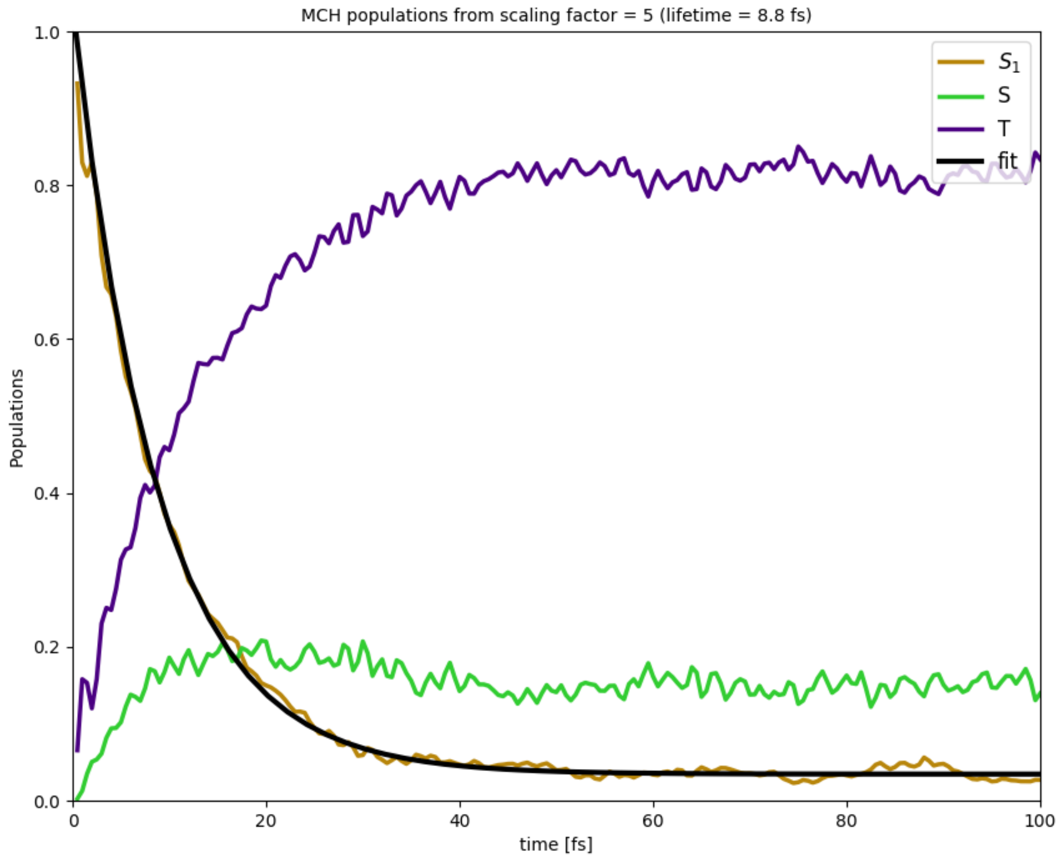

Figure S27. Time evolution of populations of singlets and triplets in I-BODIPY for excited state dynamics started in excited state S_1 using scaling factor 5

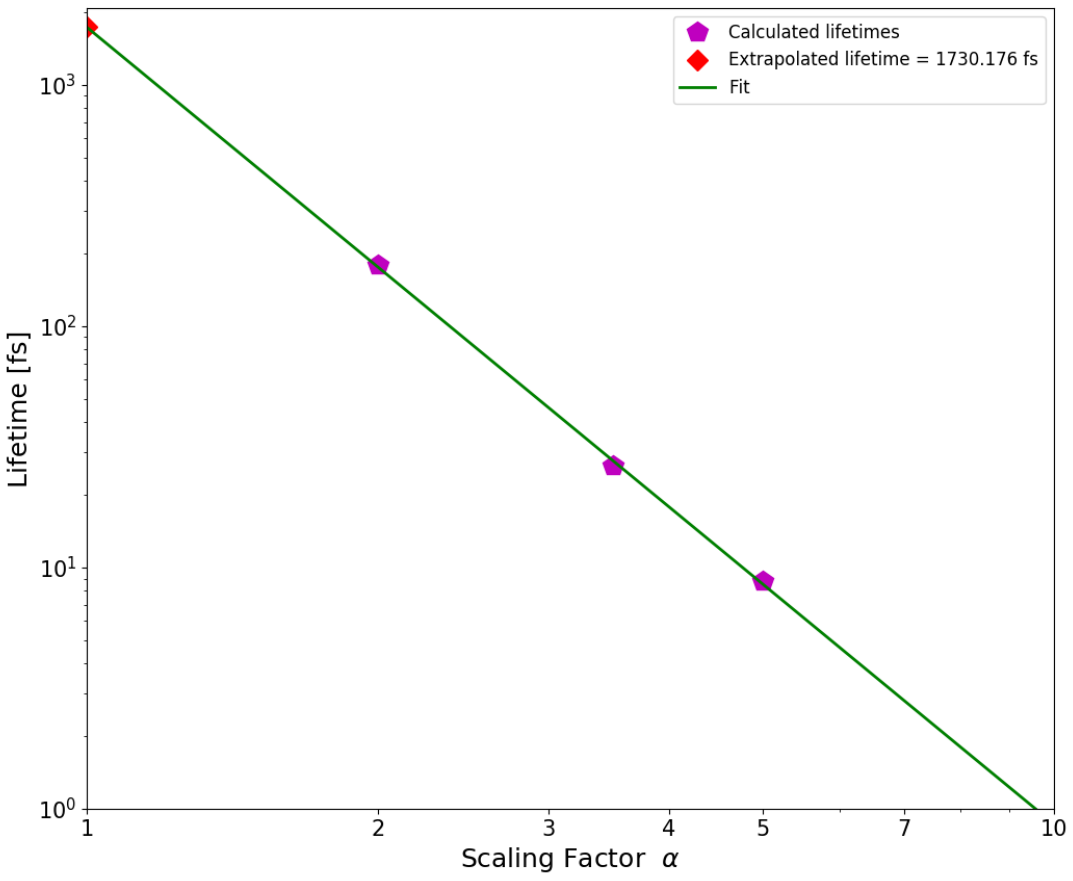

Figure S28. Extrapolation of the lifetime of the S_1 state to unit scaling factor

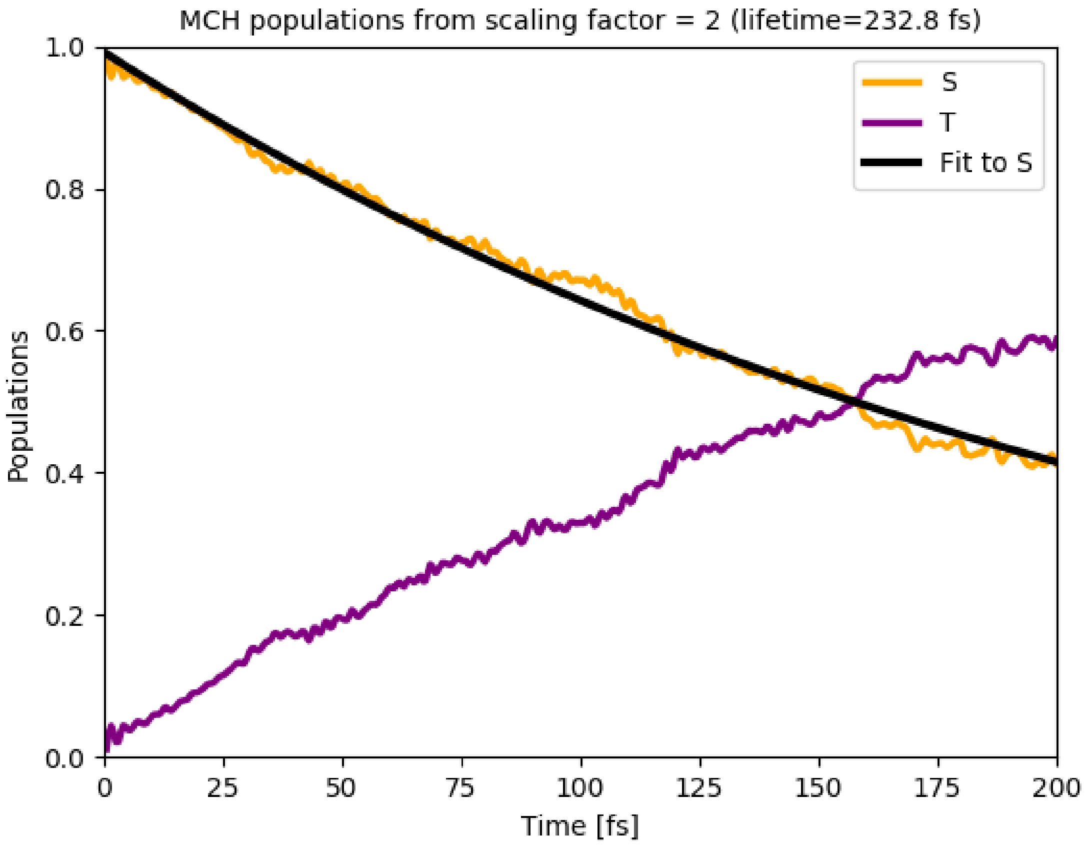

Figure S29. Time evolution of populations of singlets and triplets in I-BODIPY for excited state dynamics started in excited state S_1 using scaling factor 2

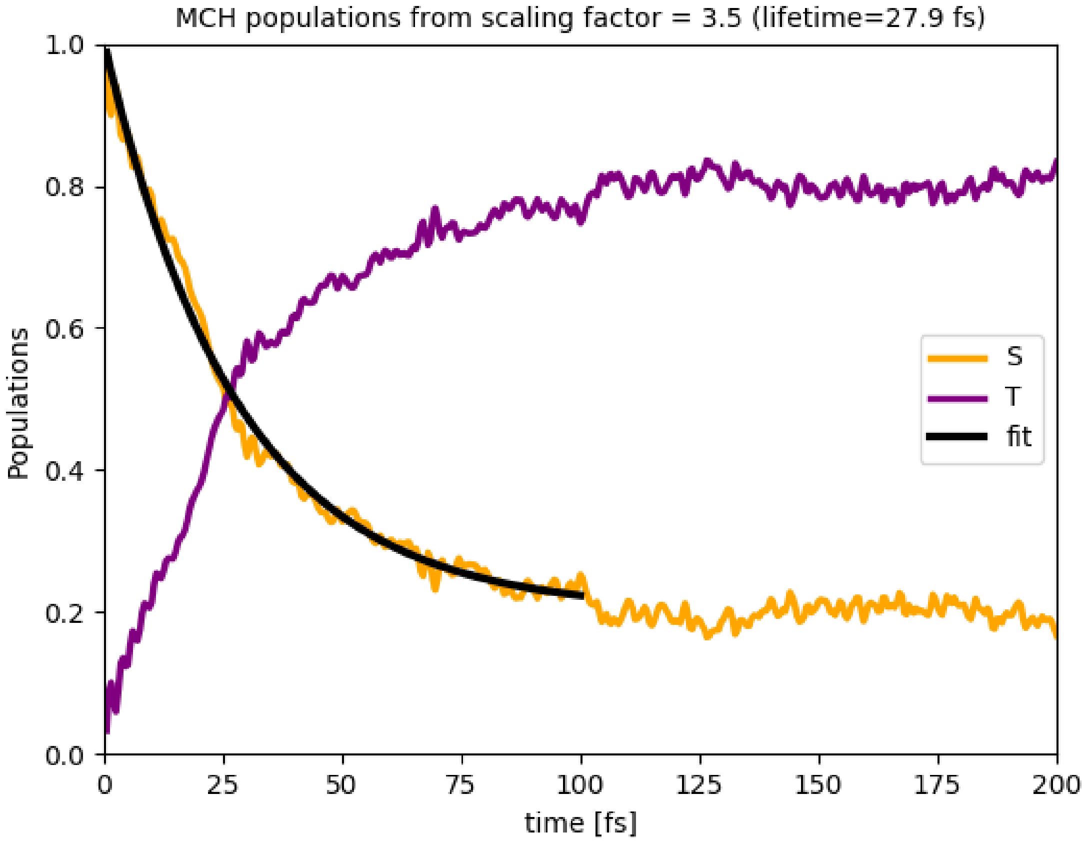

Figure S30. Time evolution of populations of singlets and triplets in I-BODIPY for excited state dynamics started in excited state S_1 using scaling factor 3.5

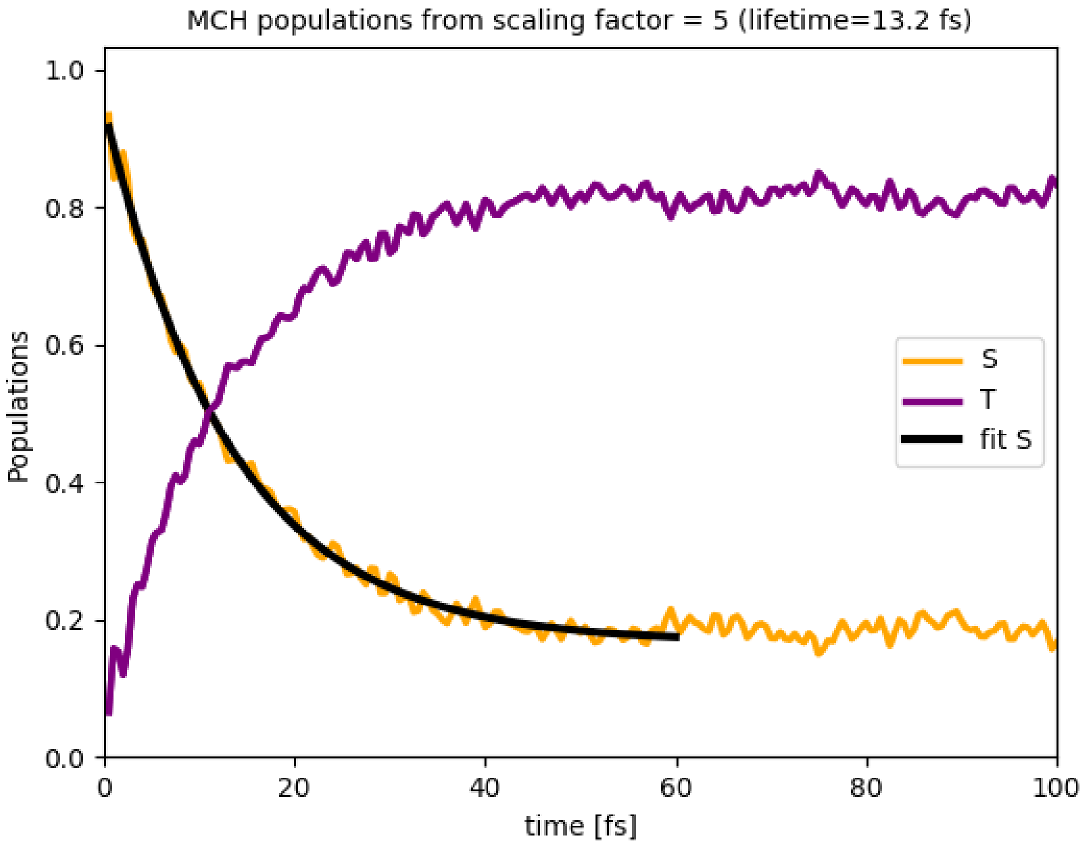

Figure S31. Time evolution of populations of singlets and triplets in I-BODIPY for excited state dynamics started in excited state S_1 using scaling factor 5

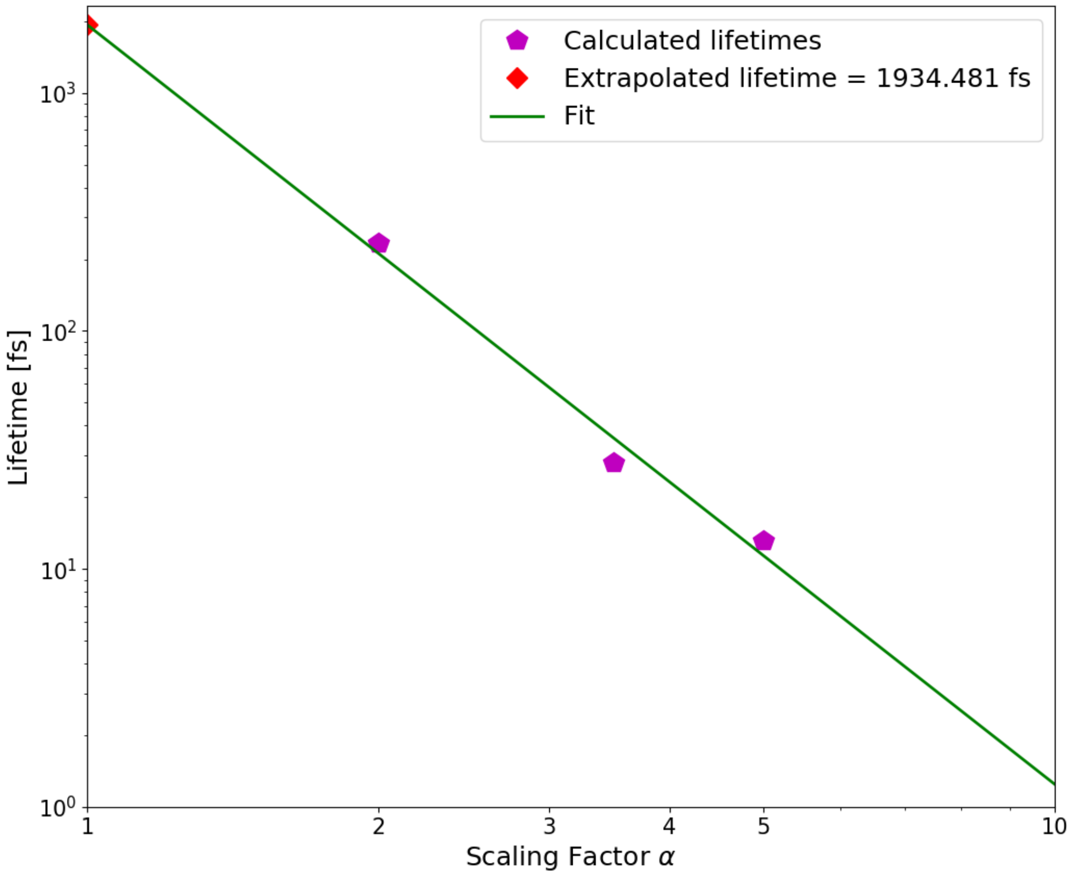

Figure S32. Extrapolation of the lifetime of the total singlet population to unit scaling factor

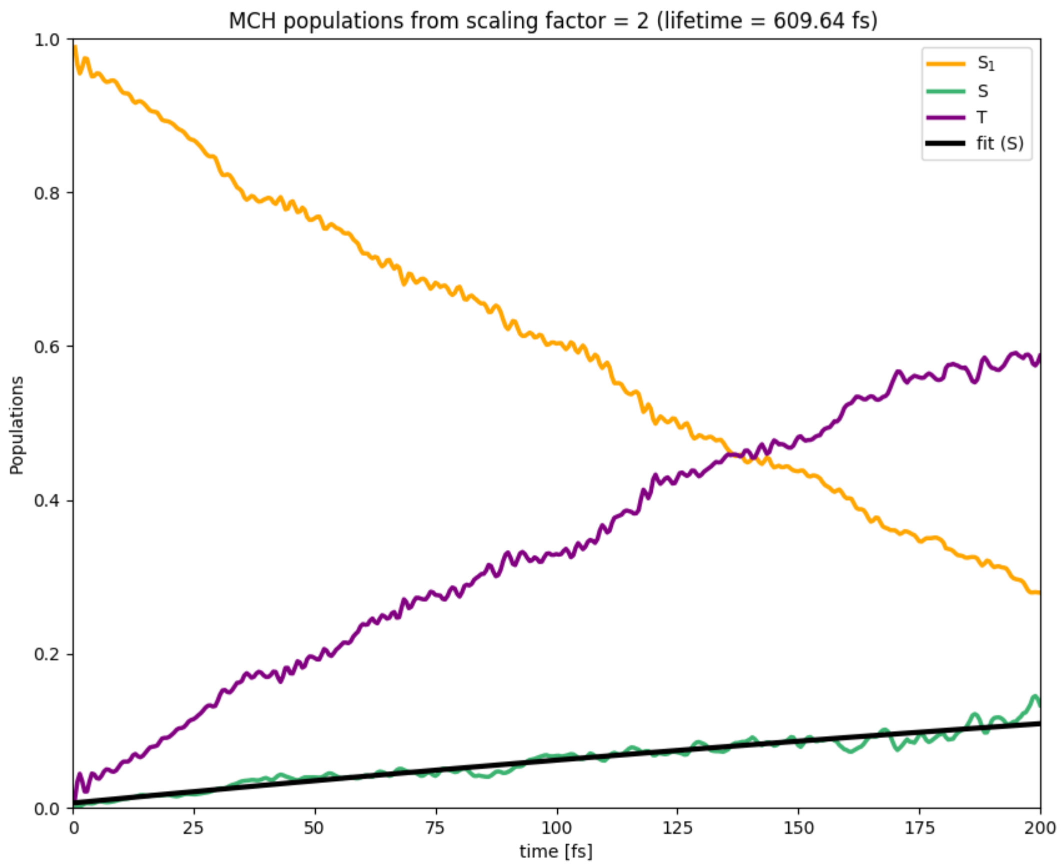

Figure S33. Time evolution of populations of singlets and triplets in I-BODIPY for excited state dynamics started in excited state S_1 using scaling factor 2

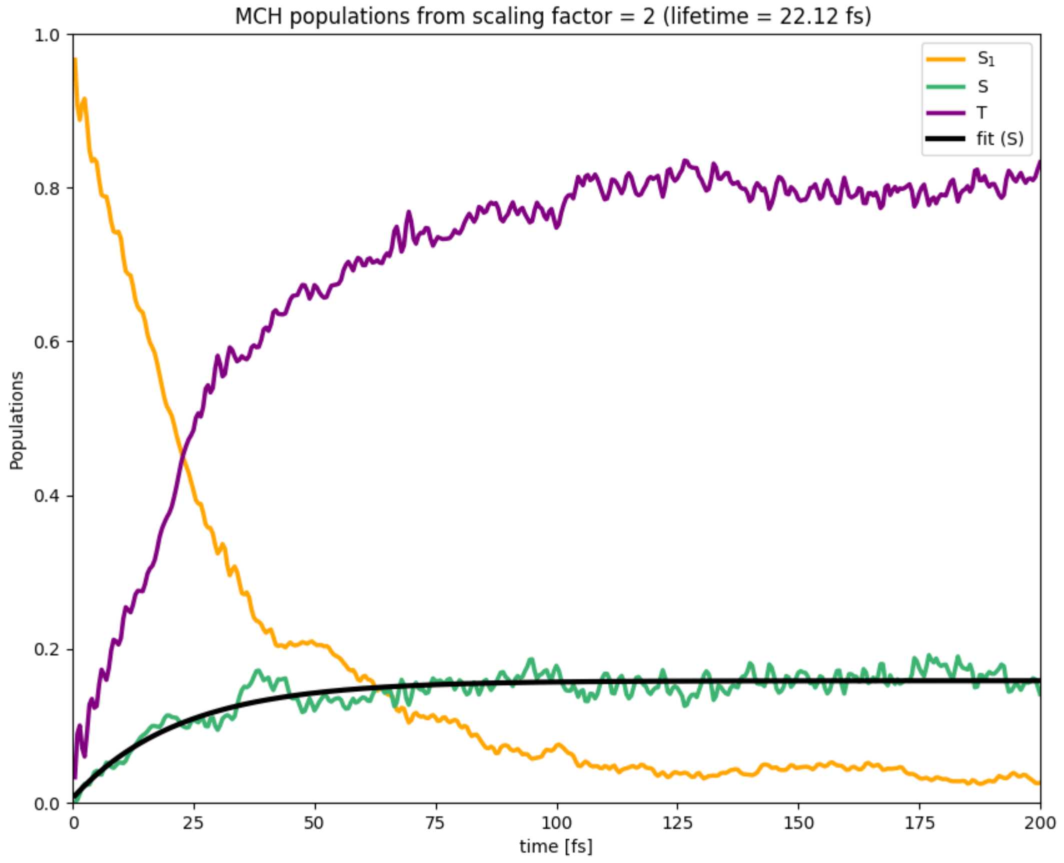

Figure S34. Time evolution of populations of singlets and triplets in I-BODIPY for excited state dynamics started in excited state S_1 using scaling factor 3.5

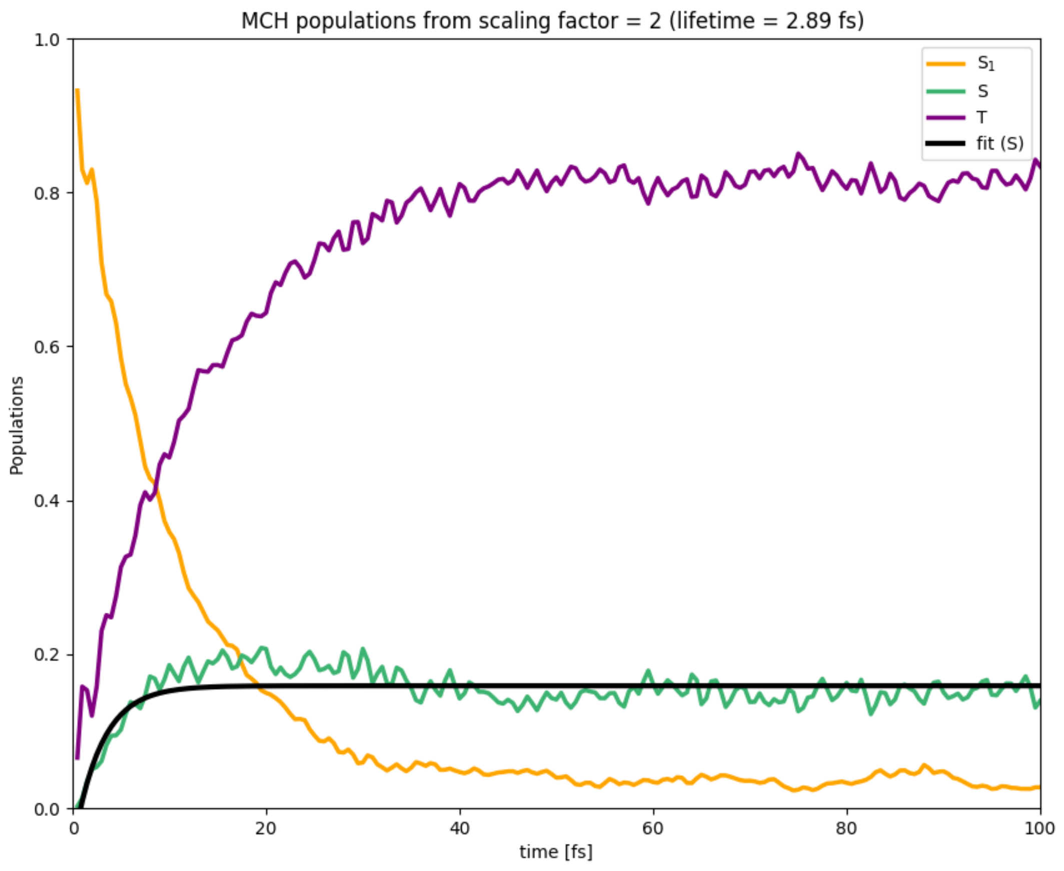

Figure S35. Time evolution of populations of singlets and triplets in I-BODIPY for excited state dynamics started in excited state S_1 using scaling factor 5

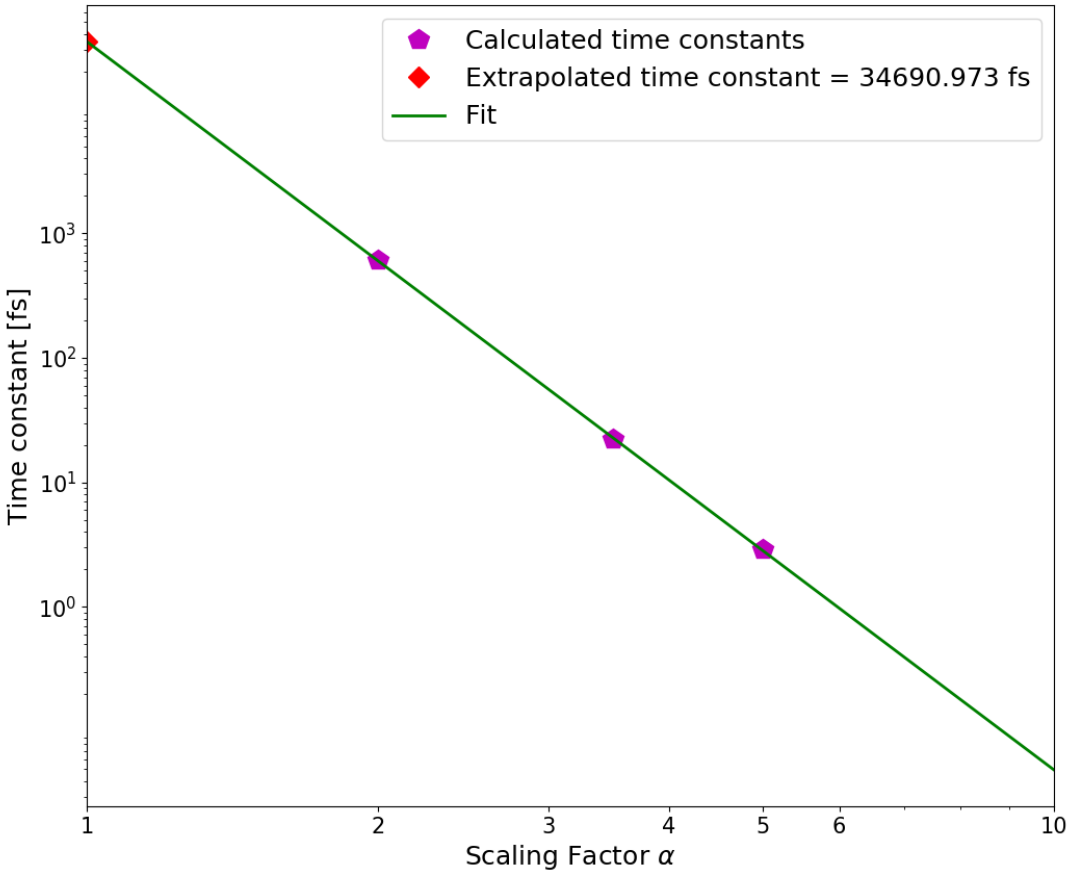

Figure S36. Extrapolation of the time constant of the overall singlet population except S_1 to unit scaling factor

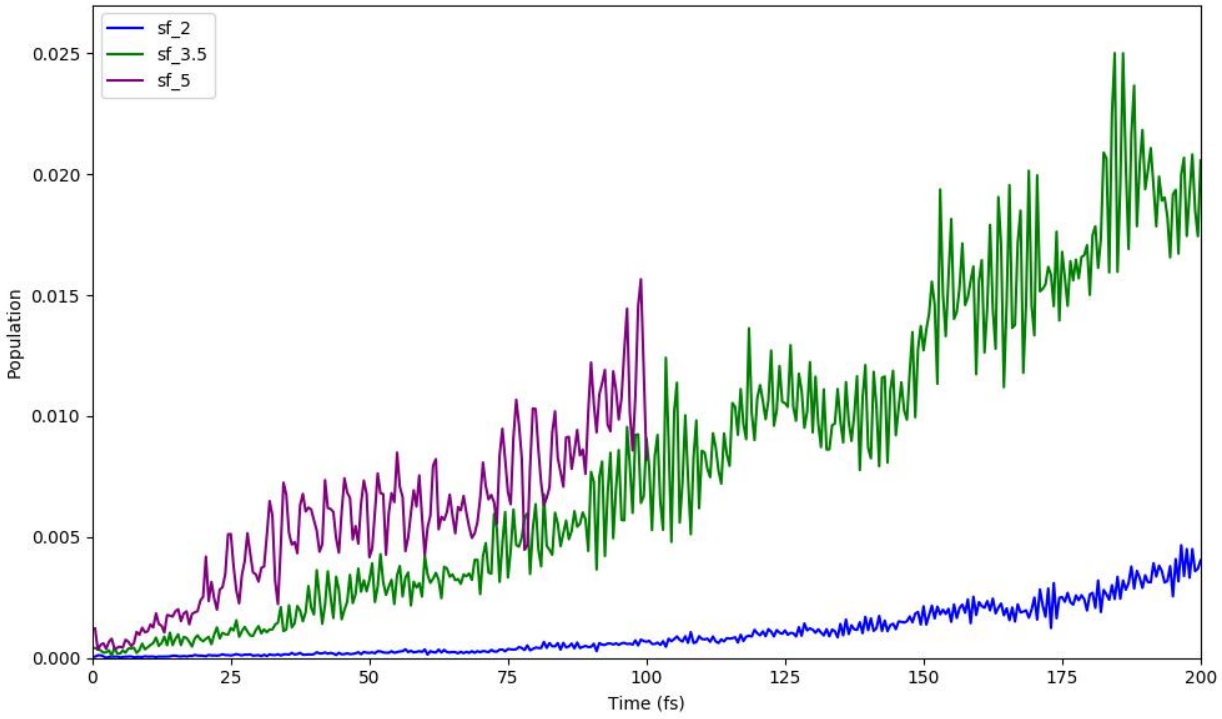

Figure S37. Evolution of populations S_0 for all three scaling factors (sf) over the entire simulation time.

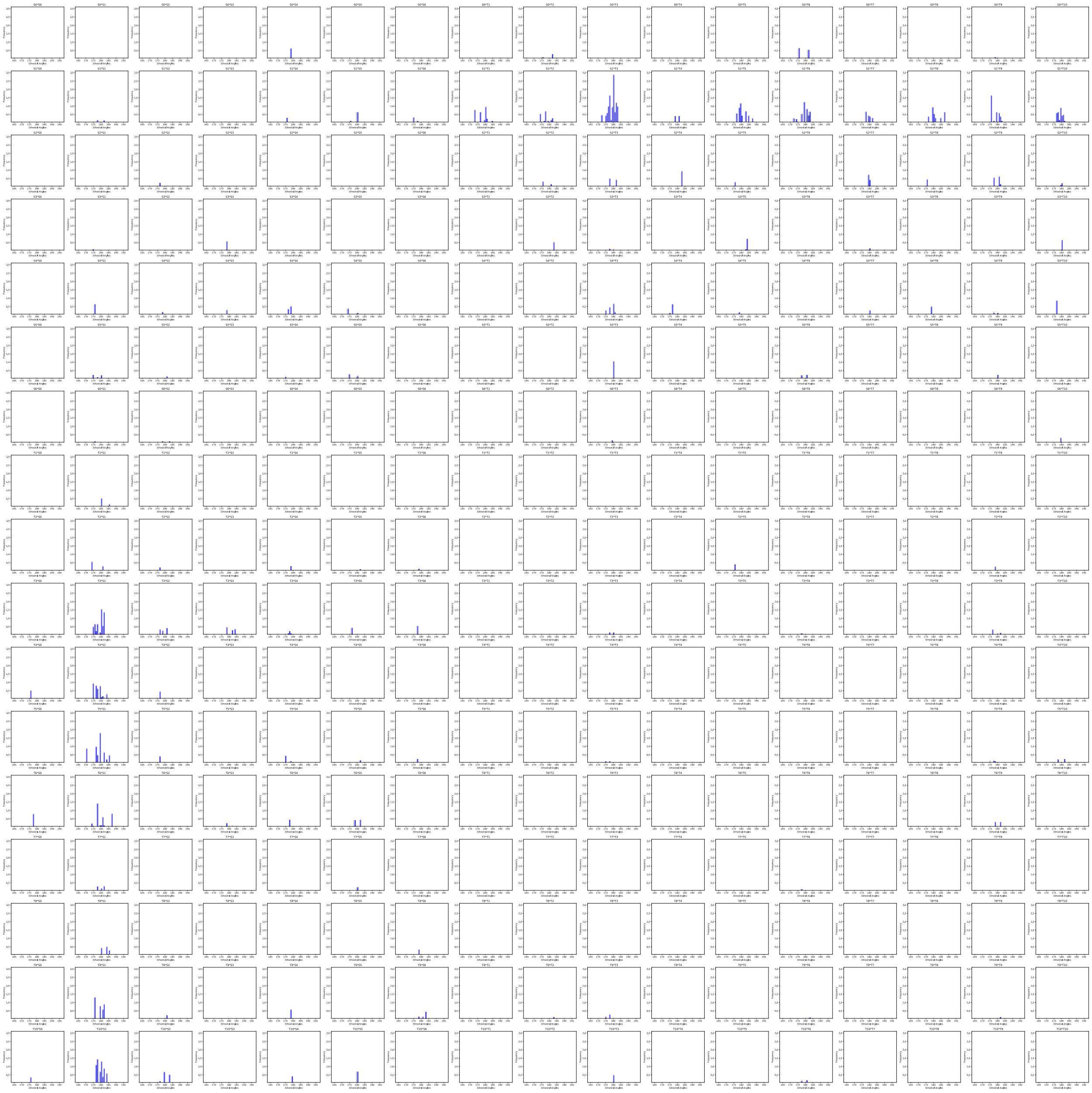

Figure S38. Matrix of histograms showing the fractional frequencies of transitions between all pairs of states (rows and columns ordered as S_0-S_6, T_1-T_{10}) as a function of the dihedral angle (see text for details)

Table S22. Fluorescence emission energy [eV] from electronic excited state S_1 to ground electronic state S_0 in I-BODIPY

States	Fluorescence emission energy [eV] (E_F)	Oscillator Strength (*f*)
$S_1 \longrightarrow S_0$	2.25	0.06

Rate constant for fluorescence is mathematically defined as;

$$k_F = \frac{2\pi e^2 E_F^2}{h^2 \varepsilon_0 m c^3} f$$

Table S23. Rate constants for all possible radiative and non-radiative photochemical processes for I-BODIPY

Photochemical phenomenon	Rate constant (s^{-1})	Method Employed
Intersystem Crossing from excited state S_1	1.65×10^{11}	Trajectory Surface Hopping Method $\left(k_{ISC}^{TSH}\right)$
Internal Conversion from excited state S_1 to all other singlets (S_0-S_6)	2.88×10^{10}	Trajectory Surface Hopping Method $\left(k_{IC}^{TSH}\right)$
Emissive decay from excited state S_1 to ground state S_0	1.32×10^{7}	$k_F = \frac{2\pi e^2 E_F^2}{h^2 \varepsilon_0 m c^3} f$

Table S24. Leading and second leading CASCI weights in the ground states of some planar unsaturated molecules calculated for state-specific CASSCF natural orbitals using the TZP contracted ANO-RCC basis set.

Molecule	Active Space	Leading Weight	Second Leading Weight
BODIPY	(6,4)	0.9710	0.0170
	(12,11)	0.8394	0.0159
Monoiodinated BODIPY (Position 2)	(6,4)	0.9707	0.0199
	(12,11)	0.8382	0.0161
	(16,14)	0.8205	0.0151
Pyrrole	(6,5)	0.9339	0.0194
2-Iodopyrrole	(6,5)	0.9338	0.0190
	(10,8)	0.9143	0.0183
Benzene	(6,6)	0.8886	0.0268
Fulvene	(6,6)	0.8730	0.0234
Naphtalene	(10,10)	0.8162	0.0195
Azulene	(10,10)	0.7715	0.0300
Anthracene	(14,14)	0.7404	0.0188
Fluorene	(12,12)	0.7889	0.0174
Phenalene	(12,12)	0.7798	0.0150

Table S25. Orbital entropies in monoiodinated BODIPY computed by DMRG.

Orbital entropies computed by DMRG
0.00773208
0.03760542
0.00848550
0.00661217
0.00927742
0.00895513
0.04985414
0.00442448
0.00575732
0.06601696
0.11877988
0.13721921
0.12890688
0.01368032
0.11684634
0.24945763
0.29726541
0.07475163
0.00631764
0.18092810
0.00423184
0.00426829
0.13467771
0.00474474
0.00557975
0.00379389
0.12413380
0.00630708
0.03729784
0.01836661

Table S26. Orbital entropies in I-BODIPY computed by DMRG.

Orbital entropies computed by DMRG
0.01167575
0.01108050
0.01904563
0.01845215
0.00621430
0.01170264
0.01115083
0.02694420
0.00889872
0.01251517
0.02629546
0.02503590
0.02861417
0.05353056
0.01867683
0.08298607
0.08727715
0.13156104
0.01876879
0.09950085
0.01964089
0.11631518
0.09400009
0.24359040
0.28950457
0.09950810
0.00967959
0.08059509
0.01786873
0.13357890
0.06745873
0.02839748
0.05850051
0.08485443
0.00619368
0.02810880
0.01354781
0.06660888
0.01369278
0.02804484
0.02551423
0.01355629
0.01428985
0.03795330
0.02676967
0.03306580

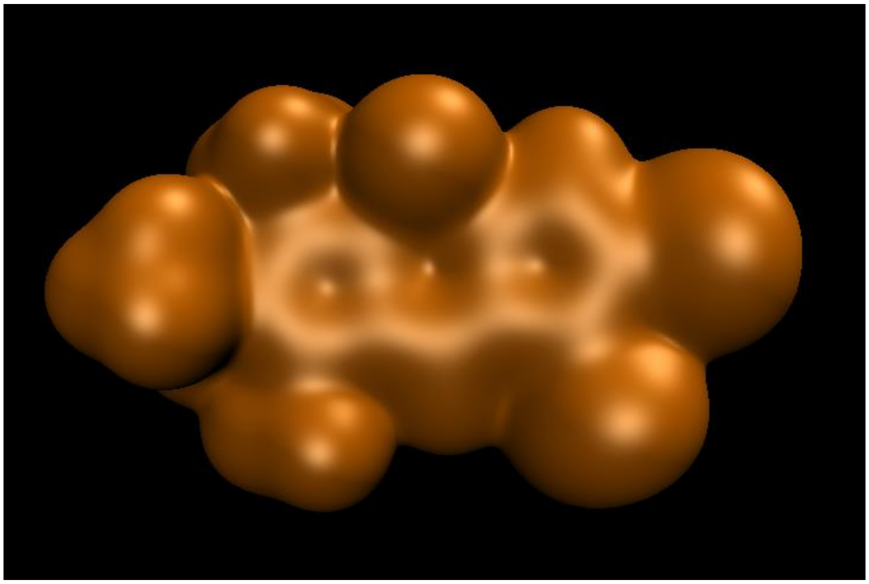

Figure S39. Electron density of ground state S_0 computed at sf-X2C-S-TD-DFT/B3LYP/x2c-TZVPPall level for Br-BODIPY

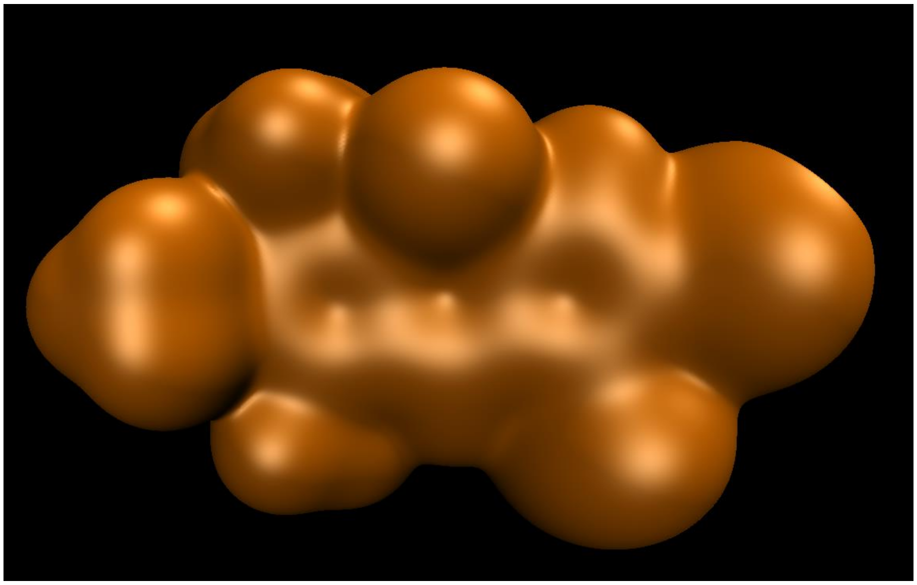

Figure S40. Electron density of ground state S_0 computed at sf-X2C-S-TD-DFT/B3LYP/x2c-TZVPPall level for I-BODIPY

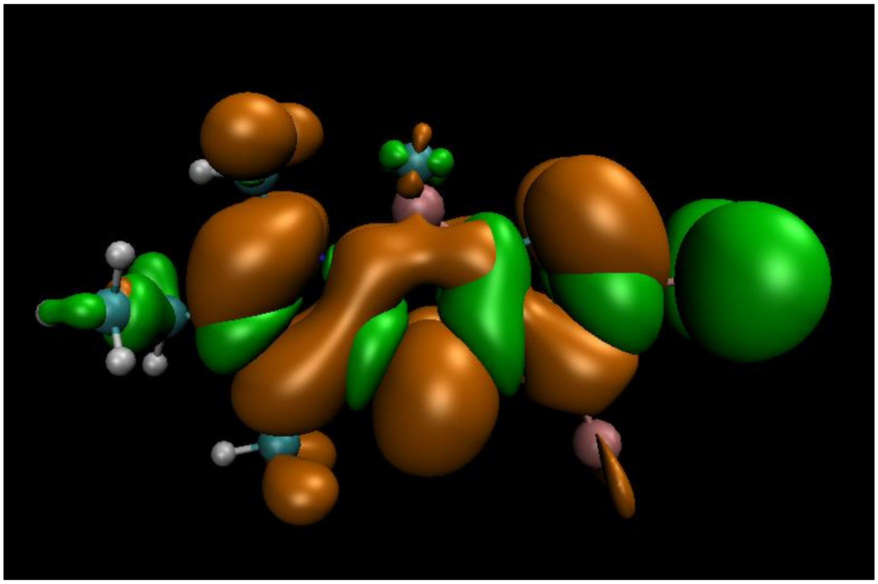

Figure S41. Differential Electron density between excited state S_1 and ground state S_0 computed at sf-X2C-S-TD-DFT/B3LYP/x2c-TZVPPall level for Br-BODIPY

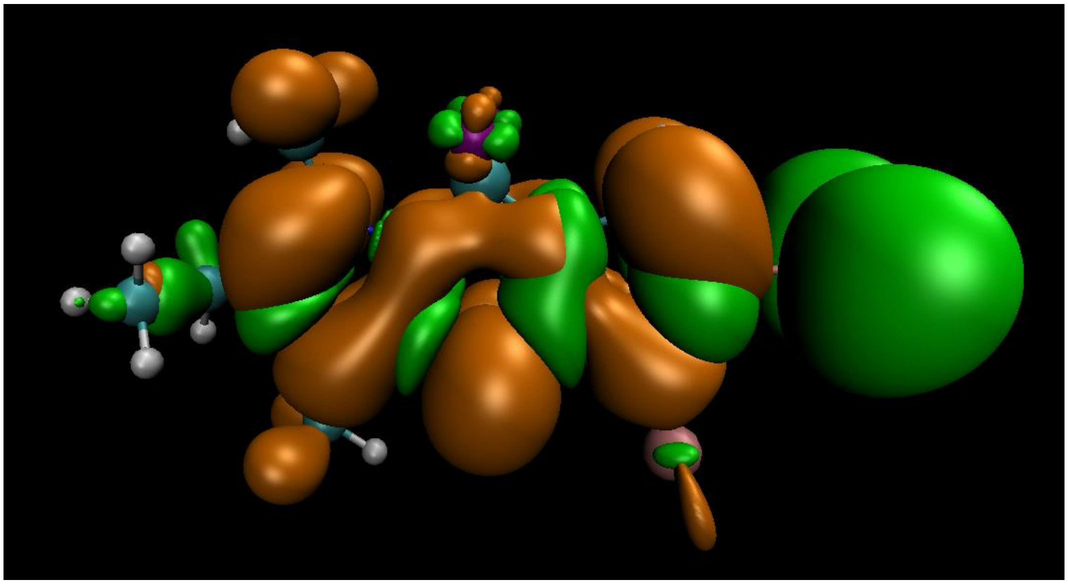

Figure S42. Differential Electron density between excited state S_1 and ground state S_0 computed at sf-X2C-S-TD-DFT/B3LYP/x2c-TZVPPall level for I-BODIPY

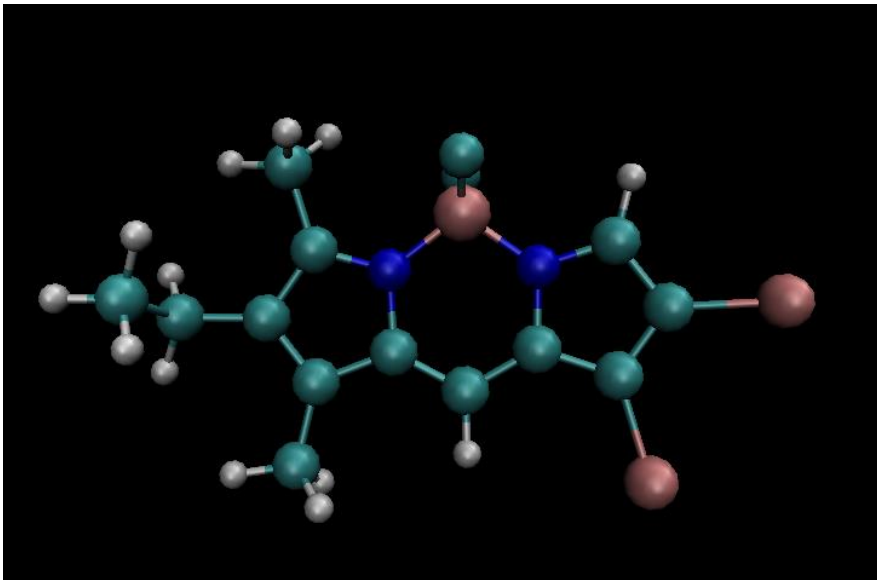

Figure S43. Optimized ground-state structure of Br-BODIPY

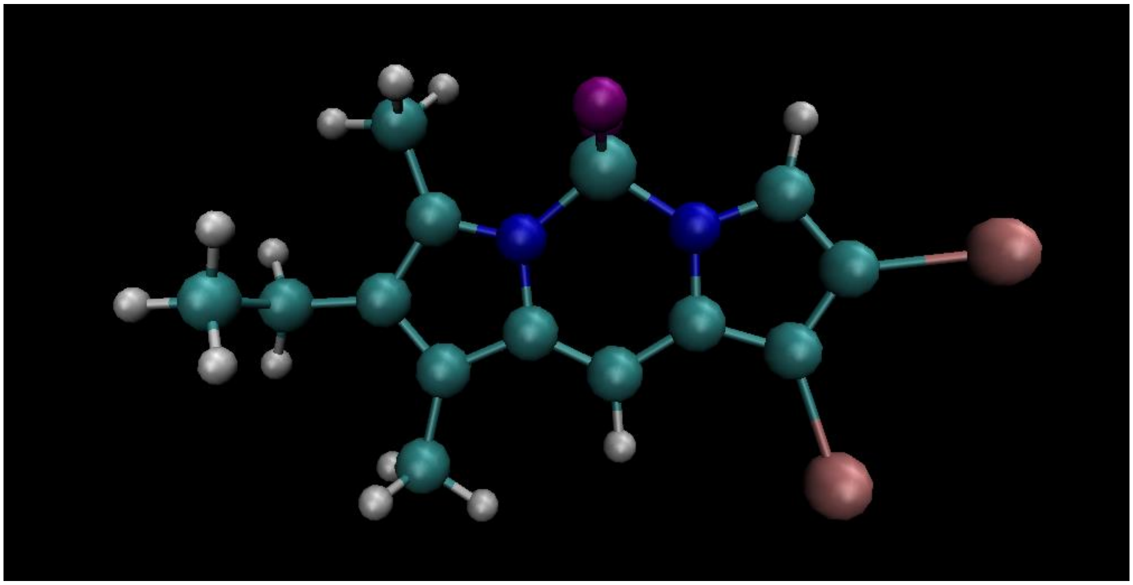

Figure S44. Optimized ground-state structure of I-BODIPY

I-BODIPY S_0 optimized at TD-DFT/B3LYP/dhf-TZVP (-1428.75003134877 a.u.)

C	1.0310686	0.0026563	-0.3128995
C	-0.3840716	0.0761644	-0.1380116
N	-0.6564173	0.0322659	1.2297158
C	0.5067556	-0.0593343	1.8839191
C	1.5853526	-0.0848172	0.9527241
C	-1.3993347	0.1794585	-1.0577615
C	-2.7396965	0.2512817	-0.6715018
C	-3.9295798	0.3421514	-1.4296782
C	-4.9890907	0.3706212	-0.5187069
C	-4.4220502	0.2981024	0.7610320
N	-3.0883147	0.2285600	0.6675505
I	-4.0148450	0.4121600	-3.5083505
B	-2.0832953	0.1339167	1.8687646
F	-2.1657192	1.2774847	2.6511173
I	-7.0385886	0.4930985	-0.8758732
C	0.5785106	-0.1004237	3.3680077
C	3.0398537	-0.1347135	1.3058784
C	3.6029783	1.2408319	1.6947444
F	-2.3644446	-0.9996351	2.6196631
H	1.5723261	-0.3893904	3.7047682
H	0.3425827	0.8846829	3.7798255
H	-4.9214599	0.2932487	1.7160176
H	-1.1667258	0.2066762	-2.1132111
H	3.6043541	-0.5311562	0.4581035
H	4.6628333	1.1743119	1.9488619
H	3.4920558	1.9504724	0.8723356
H	3.0721986	1.6499083	2.5567119
H	3.1989144	-0.8372532	2.1289228
H	-0.1597513	-0.7957956	3.7684885
C	1.7597498	0.0286175	-1.6154682
H	1.0787728	0.0082147	-2.4656910
H	2.3742966	0.9285231	-1.7036300
H	2.4314526	-0.8284302	-1.7063610

I-BODIPY S_0 optimized at TD-DFT/B3LYP/aug-cc-pVDZ/aug-cc-pVDZ-PP (-1429.165398 a.u.)

C	1.039148	0.003316	-0.327268
C	-0.382155	0.079785	-0.153380
N	-0.656011	0.052263	1.218436
C	0.510361	-0.034239	1.879320
C	1.594282	-0.068950	0.946567
C	-1.404139	0.170759	-1.077427
C	-2.748481	0.242533	-0.680002
C	-3.949257	0.342506	-1.431379
C	-5.006140	0.380901	-0.506250
C	-4.425579	0.303658	0.775117
N	-3.087790	0.222305	0.666338
I	-4.049485	0.415874	-3.523995
B	-2.078737	0.113512	1.854499
F	-2.190617	1.241793	2.681581
I	-7.071553	0.524322	-0.850323
C	0.574377	-0.070072	3.367532
C	3.051646	-0.123969	1.302982
C	3.610947	1.245328	1.730031
F	-2.335097	-1.055900	2.588523
H	1.590249	-0.287116	3.711018
H	0.256038	0.898341	3.778997
H	-4.917909	0.304217	1.741391
H	-1.175365	0.189847	-2.140670
H	3.620236	-0.497928	0.440118
H	4.678088	1.174396	1.981122
H	3.494091	1.981448	0.922820
H	3.075923	1.628844	2.609526
H	3.209983	-0.852113	2.112551
H	-0.118513	-0.824423	3.761190
C	1.772915	0.001819	-1.632276
H	1.090669	0.071828	-2.486921
H	2.474862	0.845980	-1.691190
H	2.363683	-0.918408	-1.748570

I-BODIPY S_1 optimized at TD-DFT/B3LYP/aug-cc-pVDZ/aug-cc-pVDZ-PP (-1429.155400 a.u.)

C	1.056989	0.008523	-0.313593
C	-0.371234	0.097882	-0.169584
N	-0.667287	0.050424	1.204206
C	0.501044	-0.063358	1.891642
C	1.589352	-0.095822	0.974373
C	-1.352472	0.212329	-1.127391
C	-2.742830	0.286532	-0.713833
C	-3.908913	0.401170	-1.433359
C	-5.012906	0.427567	-0.437251
C	-4.426811	0.326194	0.814437
N	-3.081759	0.243236	0.639484
I	-4.068919	0.514879	-3.505626
B	-2.062766	0.115504	1.829765
F	-2.213692	1.239765	2.666777
I	-7.028144	0.582307	-0.804697
C	0.528575	-0.128541	3.379201
C	3.039095	-0.173560	1.356506
C	3.645644	1.207059	1.667379
F	-2.372014	-1.052785	2.555402
H	1.553315	-0.237880	3.747390
H	0.087875	0.781738	3.811898
H	-4.877008	0.308219	1.798343
H	-1.105462	0.247893	-2.182472
H	3.607305	-0.647632	0.543832
H	4.706469	1.120956	1.941889
H	3.565932	1.869073	0.794129
H	3.111564	1.687379	2.498751
H	3.157511	-0.829509	2.231305
H	-0.078088	-0.971073	3.741621
C	1.768382	0.036275	-1.627827
H	1.438190	-0.786126	-2.282012
H	1.570176	0.974598	-2.170049
H	2.853558	-0.050758	-1.500627

I-BODIPY T_2 optimized at TD-DFT/B3LYP/aug-cc-pVDZ/aug-cc-pVDZ-PP (-1429.157633 a.u.)

C	1.064983	0.002717	-0.295870
C	-0.384143	0.092645	-0.117753
N	-0.664395	0.053808	1.201935
C	0.542982	-0.060385	1.907766
C	1.618507	-0.094838	0.972719
C	-1.401310	0.205936	-1.109975
C	-2.727130	0.278980	-0.684839
C	-3.960593	0.397832	-1.443155
C	-5.006215	0.430339	-0.518883
C	-4.430267	0.334120	0.765732
N	-3.068004	0.244265	0.652213
I	-4.039490	0.494725	-3.528495
B	-2.080263	0.122975	1.837390
F	-2.177728	1.246409	2.681657
I	-7.068809	0.588524	-0.854828
C	0.560030	-0.119818	3.389027
C	3.072356	-0.171086	1.340193
C	3.661486	1.204191	1.701740
F	-2.337385	-1.046512	2.579037
H	1.581456	-0.224164	3.766968
H	0.104585	0.789575	3.808506
H	-4.920191	0.325637	1.732566
H	-1.149739	0.234790	-2.163485
H	3.637792	-0.606266	0.505028
H	4.723190	1.118250	1.970095
H	3.573426	1.897984	0.854559
H	3.126025	1.647125	2.552599
H	3.201094	-0.860231	2.187409
H	-0.053506	-0.961028	3.743219
C	1.727807	0.027203	-1.632164
H	1.377657	-0.804401	-2.263715
H	1.489033	0.957013	-2.172231
H	2.817433	-0.043851	-1.544956

Br-BODIPY S_0 optimized at TD-DFT/B3LYP/aug-cc-pVDZ/aug-cc-pVDZ-PP (-1671.377451 a.u.)

C	-3.773282	0.181774	0.787968
N	-2.440657	0.136357	0.623756
C	-2.167586	0.143250	-0.748791
C	-3.415272	0.197951	-1.452914
C	-4.417784	0.222950	-0.488349
B	-1.370319	0.083988	1.758160
F	-1.553886	-1.064687	2.543277
C	-0.866824	0.097620	-1.210057
C	0.219533	0.039746	-0.324993
N	0.020452	0.027599	1.048844
C	1.217614	-0.031775	1.660141
C	2.240933	-0.061033	0.694337
C	1.616361	-0.016011	-0.559548
Br	4.087448	-0.145955	1.075948
Br	2.431739	-0.026589	-2.257508
C	-3.592726	0.224562	-2.939261
C	-5.901396	0.240462	-0.717717
C	-6.542223	-1.151302	-0.568074
C	-4.403524	0.174355	2.138129
F	-1.449929	1.236892	2.555875
H	-5.480701	0.352956	2.068936
H	-4.230989	-0.795419	2.626080
H	-2.633953	0.177810	-3.467866
H	-4.206688	-0.621909	-3.278645
H	-4.106373	1.143913	-3.256145
H	1.308018	-0.051137	2.740445
H	-0.663939	0.104097	-2.278619
H	-6.106102	0.630719	-1.724437
H	-7.625632	-1.107531	-0.744625
H	-6.103773	-1.859782	-1.284281
H	-6.374556	-1.552212	0.440893
H	-6.378716	0.940216	-0.015478
H	-3.942314	0.937047	2.778304

Calculation of matrix elements of the spin–orbit Hamiltonian

A new program has been developed for the evaluation of matrix elements of an effective one-electron spin–orbit Hamiltonian between singlet and triplet (or triplet and another triplet) CIS (auxiliary) wave functions. The program deals with excitations from a closed-shell Slater determinant, as well as excitations from a triplet Slater determinant with two unpaired electrons, both spin restricted and unrestricted.

The singlet n-electron basis is formed from the spin-orbital basis $\boldsymbol{\psi} = (\, \boldsymbol{\phi}\alpha \quad \boldsymbol{\phi}\beta \,)$, where $\boldsymbol{\phi}$ is the basis of the m singlet molecular orbitals, and comprises the reference closed-shell Slater determinant

$$|\mathbf{t}\rangle \leftrightarrow |\, 1 \quad \ldots \quad p \quad \bar{1} \quad \ldots \quad \bar{p}\, |,$$

$n = 2p$, together with all single excitations

$$\tfrac{1}{\sqrt{2}}(\mathrm{a}^{\dagger}_{j\alpha}\mathrm{a}_{i\alpha} + \mathrm{a}^{\dagger}_{j\beta}\mathrm{a}_{i\beta})|\mathbf{t}\rangle,$$

$i = 1, \ldots, p$, $j = p+1, \ldots, m$.

The triplet n-electron basis (with maximum spin projection) is built up from the spin-orbital basis $\boldsymbol{\xi} = (\, \boldsymbol{\omega}\alpha \quad \boldsymbol{\tau}\beta \,)$, where $\boldsymbol{\omega}$ and $\boldsymbol{\tau}$ are the bases of the m alpha and m beta molecular orbitals, respectively, and consists *either* of all single $\beta \to \alpha$ excitations from the closed-shell Slater determinant

$$|\mathbf{t}\rangle \leftrightarrow |\, 1 \quad \ldots \quad p \quad \bar{1} \quad \ldots \quad \bar{p}\, |,$$

$n = 2p$,

$$\mathrm{a}^{\dagger}_{j\alpha}\mathrm{a}_{i\beta}|\mathbf{t}\rangle,$$

$i = 1, \ldots, p$, $j = p+1, \ldots, m$, *or* of the reference Slater determinant

$$\mathrm{a}^{\dagger}_{p+1,\alpha}\mathrm{a}_{p\beta}|\mathbf{t}\rangle,$$

all single $\alpha \to \alpha$ excitations

$$\mathrm{a}^{\dagger}_{j\alpha}\mathrm{a}_{i\alpha}\mathrm{a}^{\dagger}_{p+1,\alpha}\mathrm{a}_{p\beta}|\mathbf{t}\rangle,$$

$i = 1, \ldots, p+1$, $j = p+2, \ldots, m$, and all single $\beta \to \beta$ excitations

$$\mathrm{a}^{\dagger}_{j\beta}\mathrm{a}_{i\beta}\mathrm{a}^{\dagger}_{p+1,\alpha}\mathrm{a}_{p\beta}|\mathbf{t}\rangle,$$

$i = 1, \ldots, p-1$, $j = p, \ldots, m$. These bases are generally only pure triplet bases if $\boldsymbol{\omega} = \boldsymbol{\tau}$. For $\boldsymbol{\omega} \neq \boldsymbol{\tau}$ the matrix of the total spin square operator $\hat{S}^2$ is evaluated in the basis of the approximate 'triplet' eigenfunctions of the nonrelativistic Hamiltonian to check if it is reasonable to proceed with the calculation of the matrix elements of the spin–orbit Hamiltonian.

The admissible CIS wave functions are therefore of the general formulae

$$|\mathrm{S}; c_0, \mathbf{C}\rangle = c_0|\mathbf{t}\rangle + \frac{1}{\sqrt{2}}\sum_{i=1}^{p}\sum_{j=p+1}^{m} c_{ji}(\mathrm{a}^{\dagger}_{j\alpha}\mathrm{a}_{i\alpha} + \mathrm{a}^{\dagger}_{j\beta}\mathrm{a}_{i\beta})|\mathbf{t}\rangle, \qquad (1)$$

where $\mathbf{C}$ is the $(m-p)$-by-p matrix of the singlet CI expansion coefficients, $\mathbf{C} = \{c_{p+j,i}\}$, $i = 1, \ldots, p$, $j = 1, \ldots, m-p$,

$$|\mathrm{T}; \mathbf{C}\rangle = \sum_{i=1}^{p} \sum_{j=p+1}^{m} c_{ji} \mathrm{a}_{j\alpha}^{\dagger} \mathrm{a}_{i\beta} |\mathbf{t}\rangle, \tag{2}$$

where $\mathbf{C}$ is the $(m-p)$-by-p matrix of the triplet CI expansion coefficients, $\mathbf{C} = \{c_{p+j,i}\}$, $i = 1, \ldots, p$, $j = 1, \ldots, m-p$, and

$$|\mathrm{T}; c_0, \mathbf{C}_\alpha, \mathbf{C}_\beta\rangle = \left(c_0 + \sum_{i=1}^{p+1} \sum_{j=p+2}^{m} c_{\alpha ji} \mathrm{a}_{j\alpha}^{\dagger} \mathrm{a}_{i\alpha} + \sum_{i=1}^{p-1} \sum_{j=p}^{m} c_{\beta ji} \mathrm{a}_{j\beta}^{\dagger} \mathrm{a}_{i\beta} \right) \mathrm{a}_{p+1,\alpha}^{\dagger} \mathrm{a}_{p\beta} |\mathbf{t}\rangle, \tag{3}$$

where $\mathbf{C}_\alpha$ is the $(m-p-1)$-by-$(p+1)$ matrix of the one set of the triplet CI expansion coefficients, $\mathbf{C}_\alpha = \{c_{\alpha,p+j+1,i}\}$, $i = 1, \ldots, p+1$, $j = 1, \ldots, m-p-1$, and $\mathbf{C}_\beta$ is the $(m-p+1)$-by-$(p-1)$ matrix of the other set of the triplet CI expansion coefficients, $\mathbf{C}_\beta = \{c_{\beta,p+j-1,i}\}$, $i = 1, \ldots, p-1$, $j = 1, \ldots, m-p+1$.

While the wave functions (1) and (2) are both true restricted active space (RAS) wave functions [1], namely RAS$(p : 1, 0, m-p : 1)$, the wave function (3) is actually a full class subset of the RAS$(p-1 : 1, 2, m-p-1 : 1)$ wave function. Although these wave functions may be built up from three different and independent sets of (singlet, alpha and beta) molecular orbitals, the related matrix elements are still calculated 'exactly', on the assumption that the triplet wave functions are eigenfunctions of the total spin square operator, through the biorthonormalization of the two pairs of sets of molecular orbitals (singlet/alpha and singlet/beta) followed by the counter-transformation of the corresponding singlet and triplet CIS expansion coefficients [2].

In a nutshell, if $\boldsymbol{\phi} \neq \boldsymbol{\omega}$ and/or $\boldsymbol{\phi} \neq \boldsymbol{\tau}$, the pair of bases of the molecular orbitals $\boldsymbol{\phi}$ and $\boldsymbol{\eta}$ (either $\boldsymbol{\omega}$ or $\boldsymbol{\tau}$) is, through the LU decomposition of the transition overlap matrix $\mathbf{S} = \langle \boldsymbol{\phi} | \boldsymbol{\eta} \rangle$,

$$\mathbf{PS} = \mathbf{LU}, \tag{4}$$

$l_{ii} = 1$, $i = 1, \ldots, m$, transformed to the new pair of bases, say,

$$\boldsymbol{\vartheta} = \boldsymbol{\phi} \mathbf{P}^{\mathrm{T}} \mathbf{L}^{-\dagger} \tag{5}$$

and

$$\boldsymbol{\varrho} = \boldsymbol{\eta} \mathbf{U}^{-1}, \tag{6}$$

which are mutually biorthonormal,

$$\langle \boldsymbol{\vartheta} | \boldsymbol{\varrho} \rangle = \mathbf{L}^{-1} \mathbf{P} \langle \boldsymbol{\phi} | \boldsymbol{\eta} \rangle \mathbf{U}^{-1} = \mathbf{L}^{-1} \mathbf{PSU}^{-1} = \mathbf{1}. \tag{7}$$

This is the necessary first step towards the efficient evaluation of the matrix elements between eigenfunctions of the nonrelativistic Hamiltonian of different spin multiplicities (unless the molecular orbitals are not allowed to relax in the course of the HF or KS SCF calculations). The second step is the counter-transformation of the CI expansion coefficients of the singlet and triplet, whose effect is to compensate for the orbital alterations so as to keep the multielectron wave functions unchanged [3, 4, 5].

The employed effective one-electron spin–orbit Hamiltonian is the sum of the Breit–Pauli (or Douglas–Kroll–Hess) one-electron spin–orbit Hamiltonian [6] (which itself is a sum of contributions of individual atoms of a molecule) and an approximation to the "two-electron" part of the 'exact' Breit–Pauli (or Douglas–Kroll–Hess) one-electron mean-field spin–orbit Hamiltonian [7]. The approximation is a gentle modification of the so-called flexible nuclear screening spin–orbit (FNSSO) approximation [8] showing mean relative error below a few percent for virtually all elements of the periodic table in most bonding situations. The accuracy of the approximation has proven [8, 9] to be comparable to the popular, but significantly more expensive, atomic mean-field integral (AMFI) approximation [10]. The calculation of the demanding two-electron spin–orbit integrals is nevertheless completely avoided. As far as speed is concerned, the approximation almost comes up to the simplest one-electron effective nuclear charge approaches [11] from which it differs in two important aspects: the effective nuclear charge is represented by a continuous multivariable basis set independent function rather than by a single real number per element, and both one- and two-center contributions are taken into account.

A more descriptive view at the approximation is based on the explicit form of e.g. the Breit–Pauli one-electron spin–orbit Hamiltonian [6] (in SI units)

$$\hat{h}(1) = \frac{e^2}{8\pi\varepsilon_0 m^2 c^2} \sum_C Z_C r_C^{-3} \left(\mathbf{r}_C \times \hat{\mathbf{p}}\right) \cdot \hat{\mathbf{s}}, \tag{8}$$

where ε_0 is the permittivity of vacuum, c is the velocity of light in vacuum, e is the charge and m is the mass of an electron, Z_C is the number of protons ($Z_C e$ is the charge) of the nucleus C, $\mathbf{r}_C$ is the position vector of an electron with respect to the nucleus C, $r_C = |\mathbf{r}_C|$, and $\hat{\mathbf{p}}$ or $\hat{\mathbf{s}}$ is the operator of the vector of linear momentum or spin angular momentum of an electron, respectively. Accordingly, the employed effective spin–orbit Hamiltonian is essentially the Breit–Pauli (or Douglas–Kroll–Hess) one-electron spin–orbit Hamiltonian in which, for each nucleus C, the true nuclear charge $Z_C e$ has been replaced with an effective nuclear charge $(1 - q_C)Z_C e$, where the so-called screening quotient $q_C \equiv q_C(l, \alpha, \beta) \in \langle 0, 1\rangle$ depends not only on the nucleus C, but also on the two one-electron primitive Gaussian functions bracketing the operator in a particular one-center matrix element—through their common azimuthal quantum number l (describing their angular parts) and individual exponents α and β (describing their radial parts). Clearly, an electron close enough to the atomic nucleus experiences only the electric field brought by the positive nuclear charge $Z_C e$ (as the contributions of all the electrons of the atom to the spherically symmetric electric field at the position of the nucleus mutually cancel) while an electron in the infinity experiences just the zero electric field of the neutral atom. Therefore, the quotient q_C should almost vanish for the tightest basis functions and should approach 1 for the sufficiently diffuse ones. Moreover, the quotient q_C should never be lower than 0 or greater than 1 as the fictitious electron can nowhere in space experience a spherically symmetric electric field of a positive charge greater than $Z_C e$ or of a negative charge.

The dependence of q_C on the variables l, α and β has been determined through nonrelativistic (or scalar relativistic) full-valence CASSCF calculations on the ground

states of neutral atoms (employing large *uncontracted* one-electron bases) followed by the evaluation of the nonzero matrix elements of the 'exact' Breit–Pauli (or Douglas–Kroll–Hess) one-electron mean-field spin–orbit Hamiltonian.

The contribution of a heavy atom on which a two-component (relativistic, j-dependent) pseudopotential [12] is defined (and a reduced number of basis functions are considered to represent just the pseudovalence orbitals instead of the full set of core and valence orbitals) to matrix elements of the effective one-electron spin–orbit Hamiltonian is best evaluated using an operator implicitly included in the two-component pseudopotential itself [13].

The central idea behind the pseudopotential approximation [14, 15, 16, 17, 18, 19, 20] is to select from the total number of n (actually indistinguishable!) electrons of a molecule the q valence electrons (responsible for all the chemistry) that are moving in the electric field of the remaining $n - q$ core electrons and the positively charged nuclei (to which the core electrons are "stuck" in exactly the same way as in the isolated atoms). Heavy nuclei are thus replaced with spherical cores, while $Q_C e$ is the charge of the core whose center is at the nucleus C with the charge $Z_C e$, $0 < Q_C < Z_C$. Consequently,

$$q = n - \sum_C (Z_C - Q_C).$$

Point-like cores can be regarded as the roughest, zero-order, approximation. Therefore, the valence-only effective Hamiltonian of a molecule (in SI units)

$$\hat{H}(1, \ldots, q) = -\frac{\hbar^2}{2m} \sum_{i=1}^{q} \nabla_i^2 + \frac{e^2}{4\pi\varepsilon_0} \left\{ \sum_{i=1}^{q} \sum_C \left[-\frac{Q_C}{r_{iC}} + \Delta \mathrm{v}_C(r_{iC}) \right] \right.$$
$$\left. + \sum_{i=1}^{q} \sum_{j>i}^{q} \frac{1}{r_{ij}} + \sum_C \sum_{D>C} \frac{Q_C Q_D}{r_{CD}} \right\} \tag{9}$$

may be introduced which, in its simplest practicable form, stands for kinetic energy of the valence electrons and potential energy of the Coulombic (i) attraction between the valence electrons and the cores (taking into account also the correction for the error of the zero-order approximation by means of pre-parameterized, through all-electron *relativistic* calculations on atoms or their ions, one-electron operators $\Delta \mathrm{v}_C$ called pseudopotentials), (ii) repulsion between the valence electrons, and (iii) repulsion between the (point-like) cores, where $\hbar$ is the reduced Planck constant, ε_0 is the permittivity of vacuum, e is the charge and m is the mass of an electron, r_{iC} is the distance between the electron i and the nucleus C while r_{ij} or r_{CD} is the distance between two electrons or two nuclei (point-like cores), respectively. The energy of the cores themselves is supposed to be a constant and has been subtracted.

Pseudopotentials are thus intended to model, in a mean-field manner, core-valence interactions of electrons as well as core-valence orbital orthogonality constraints in atoms and molecules. The main benefit of using pseudopotentials is the reduction of computational demands of electronic structure calculations (largely due to the decrease of the number of one-electron basis functions) while nearly maintaining the accuracy of the

calculations. The added value is the fact that, for atoms of heavy elements, the most important relativistic effects are included in the pre-computed parameters and, in the case of scalar relativistic effects, do not even have to be treated explicitly.

For properly designed pseudopotentials, the lowest-energy eigenfunctions of the related one-electron mean-field (HF or KS) Hamiltonian describe valence states and have nodeless radial parts. Accordingly, a correctly parameterized pseudopotential effectively "shifts" relativistic effects (including, with a 2-component pseudopotential, also spin–orbit coupling) from the core to the valence region of the molecule.

The pseudopotential $\Delta \mathrm{v}_C$ is a smooth function of the distance of an electron from the nucleus C and is generally different for basis functions of different angular symmetries. It is repulsive in the short range (so as to keep the valence electrons out of the core) and attractive in the long range. Typically, Q_C differs from Z_C only for the atoms of the heaviest elements, often just one or two atoms in the molecule. For atoms with $Q_C = Z_C$ the pseudopotential $\Delta \mathrm{v}_C$ clearly vanishes and full one-electron basis is required. The contribution of such an atom to matrix elements of the effective one-electron spin–orbit Hamiltonian is then calculated using the (Breit–Pauli variant of the) *one-center* FNSSO approximation described above. For atoms with $Q_C < Z_C$ the related part of the *one-center* effective spin–orbit Hamiltonian is extracted from the two-component pseudopotential $\Delta \mathrm{v}_C$ by spin separation [13].

It should be mentioned that in the aforesaid typical case, where pseudopotentials are only defined for a subset of the atoms of a molecule, the one-center approximation to spin–orbit coupling seems to be the natural choice. Clearly, if the multicenter contributions were evaluated using an all-electron spin–orbit Hamiltonian on lighter atoms, they would be incomplete and thus incorrect due to the missing tight basis functions describing core orbitals on the heavy atoms with pseudopotentials. Then it is perhaps better not to calcualate them at all. However, if the multicenter contributions are not calculated on the lighter atoms, they should probably not be calculated on the heavy atoms either to avoid unbalanced treatment.

one-component (nonrelativistic or scalar relativistic, l-dependent) pseudopotential

$$\Delta \mathrm{v}(r) = \begin{pmatrix} \Delta v(r) & 0 \\ 0 & \Delta v(r) \end{pmatrix} \equiv \Delta v(r) = \sum_{l=0}^{\infty} v_l(r) \hat{P}_l$$

azimuthal quantum number $l \geq 0$

$$\hat{P}_l = \sum_{m=-l}^{l} |l, m\rangle\langle l, m| = \sum_{m=-l}^{l} |l; m\rangle\langle l; m|$$

magnetic quantum number $m \leq |l|$

$$|l, m\rangle \equiv Y_{lm}(\vartheta, \varphi)$$

common eigenfunctions of the operators $\hat{l}^2$ and $\hat{l}_z$, the so-called spherical harmonics

$$|l; m\rangle \equiv S_{lm}(\vartheta, \varphi)$$

real linear combinations of (complex) spherical harmonics

$$S_{l0} = Y_{l0}$$

for $m > 0$

$$S_{lm} = \frac{1}{\sqrt{2}}[(-1)^m Y_{lm} + Y_{l,-m}]$$

$$S_{l,-m} = -\frac{i}{\sqrt{2}}[(-1)^m Y_{lm} - Y_{l,-m}]$$

The projection operators $\hat{P}_l$, $l \geq 0$, ensure that each of the operators $\hat{v}_l(r)$ acts only on the components of the one-electron basis functions with the proper angular symmetry. Since spherical harmonics form a complete set of functions, the projection operators $\hat{P}_l$ fulfill the completeness relation

$$\sum_{l=0}^{\infty} \hat{P}_l = 1.$$

for $l > u \gg 0$, where $u - 1$ is the highest l found in the core

$$v_l(r) \doteq v_u(r)$$

avoiding infinite summation by exploiting the completeness relation for the projection operators $\hat{P}_l$

$$\Delta v(r) \doteq \sum_{l=0}^{u-1} v_l(r)\hat{P}_l + v_u(r)\sum_{l=u}^{\infty}\hat{P}_l$$

$$= \sum_{l=0}^{u-1} v_l(r)\hat{P}_l + v_u(r)\sum_{l=u}^{\infty}\hat{P}_l + v_u(r)\left[1 - \sum_{l=0}^{\infty}\hat{P}_l\right]$$

$$= \sum_{l=0}^{u-1} v_l(r)\hat{P}_l + v_u(r) - v_u(r)\sum_{l=0}^{u-1}\hat{P}_l$$

$$= v_u(r) + \sum_{l=0}^{u-1}\left[v_l(r) - v_u(r)\right]\hat{P}_l$$

<u>two-component (relativistic, j-dependent) pseudopotential</u>

$$\Delta \mathrm{v}(r) = \sum_{l=0}^{\infty}\sum_{j=\left|l-\frac{1}{2}\right|}^{l+\frac{1}{2}} v_{lj}(r)\mathrm{P}_{lj}$$

$$= v_{0,1/2}(r)\mathrm{P}_{0,1/2} + \sum_{l=1}^{\infty}\left[v_{l,l-1/2}(r)\mathrm{P}_{l,l-1/2} + v_{l,l+1/2}(r)\mathrm{P}_{l,l+1/2}\right]$$

spin quantum number $s = \frac{1}{2}$, $j = l \mp s = l \mp \frac{1}{2}$

$$\mathrm{P}_{lj} = \sum_{m=-j}^{j} |l,j,m\rangle\langle l,j,m|$$

coupling of angular momenta

$$\hat{\mathbf{j}} = \hat{\mathbf{l}} + \hat{\mathbf{s}}$$

common eigenfunctions of the operators $\hat{j}^2$ and $\hat{j}_z$

$$|l,j,m\rangle \equiv \left|l, l\mp\tfrac{1}{2}, m\right\rangle = \mp\sqrt{\frac{l\mp m+\frac{1}{2}}{2l+1}}Y_{l,m-1/2}\alpha + \sqrt{\frac{l\pm m+\frac{1}{2}}{2l+1}}Y_{l,m+1/2}\beta$$

$$\leftrightarrow \frac{1}{\sqrt{2l+1}}\begin{pmatrix} \mp\sqrt{l\mp m+\frac{1}{2}}\,Y_{l,m-1/2} \\ \sqrt{l\pm m+\frac{1}{2}}\,Y_{l,m+1/2}\end{pmatrix}$$

the elementary spin functions α and β are common eigenfunctions of the operators $\hat{s}^2$ and $\hat{s}_z$

$$\left|l, l\mp\tfrac{1}{2}, m\right\rangle\left\langle l, l\mp\tfrac{1}{2}, m\right| = \frac{1}{2l+1}\left\{\begin{matrix} (l\mp m+\frac{1}{2})\left|l,m-\frac{1}{2}\right\rangle\left\langle l,m-\frac{1}{2}\right| \\ \mp\sqrt{(l\mp m+\frac{1}{2})(l\pm m+\frac{1}{2})}\left|l,m+\frac{1}{2}\right\rangle\left\langle l,m-\frac{1}{2}\right| \end{matrix}\right.$$

$$\left.\begin{matrix} \mp\sqrt{(l\mp m+\frac{1}{2})(l\pm m+\frac{1}{2})}\left|l,m-\frac{1}{2}\right\rangle\left\langle l,m+\frac{1}{2}\right| \\ (l\pm m+\frac{1}{2})\left|l,m+\frac{1}{2}\right\rangle\left\langle l,m+\frac{1}{2}\right| \end{matrix}\right\}$$

P_{lj} is a 2-by-2 matrix

$$\sum_{j=\left|l-\frac{1}{2}\right|}^{l+\frac{1}{2}} \mathrm{P}_{lj} = \begin{pmatrix} \hat{P}_l & 0 \\ 0 & \hat{P}_l\end{pmatrix} \equiv \hat{P}_l$$

$$\mathrm{P}_{0,1/2} = \hat{P}_0$$

for $l > 0$

$$\mathrm{P}_{l,l-1/2} + \mathrm{P}_{l,l+1/2} = \hat{P}_l$$

$$\mathrm{P}_{l,l-1/2} = \sum_{m=-l+1/2}^{l-1/2}\left|l,l-\tfrac{1}{2},m\right\rangle\left\langle l,l-\tfrac{1}{2},m\right| = \frac{1}{2l+1}(l\hat{P}_l - \mathrm{R}_l)$$

$$\mathrm{P}_{l,l+1/2} = \sum_{m=-l-1/2}^{l+1/2}\left|l,l+\tfrac{1}{2},m\right\rangle\left\langle l,l+\tfrac{1}{2},m\right| = \frac{1}{2l+1}[(l+1)\hat{P}_l + \mathrm{R}_l]$$

$$\mathrm{R}_l = l\mathrm{P}_{l,l+1/2} - (l+1)\mathrm{P}_{l,l-1/2} = \frac{1}{\hbar}(\hat{P}_l\hat{l}_x\hat{P}_l\sigma_x + \hat{P}_l\hat{l}_y\hat{P}_l\sigma_y + \hat{P}_l\hat{l}_y\hat{P}_l\sigma_z)$$

$$= \frac{1}{\hbar}\hat{P}_l\hat{\mathbf{l}}\hat{P}_l\cdot\boldsymbol{\sigma} \leftrightarrow \frac{2}{\hbar^2}\hat{P}_l\hat{\mathbf{l}}\hat{P}_l\cdot\hat{\mathbf{s}}$$

$$\hat{P}_l\hat{l}_u\hat{P}_l = \sum_{q=-l}^{l}\sum_{m=-l}^{l}|l,q\rangle\langle l,q|\hat{l}_u|l,m\rangle\langle l,m| = \sum_{q=-l}^{l}\sum_{m=-l}^{l}|l;q\rangle\langle l;q|\hat{l}_u|l;m\rangle\langle l;m|$$

$u = x, y, z$

$$\sigma_x = \begin{pmatrix} 0 & 1 \\ 1 & 0\end{pmatrix}\ ,\quad \sigma_y = \begin{pmatrix} 0 & -i \\ i & 0\end{pmatrix}\ ,\quad \sigma_z = \begin{pmatrix} 1 & 0 \\ 0 & -1\end{pmatrix}$$

$\boldsymbol{\sigma}$ is the vector of 2-by-2 Pauli spin matrices σ_x, σ_y and σ_z

$$\frac{\hbar}{2}\boldsymbol{\sigma} \leftrightarrow \hat{\mathbf{s}}$$

a relation derived by comparing $\boldsymbol{\sigma}$ with the 2-by-2 matrix of the operator $\hat{\mathbf{s}}$ in the basis of the elementary spin functions α and β

$$\begin{pmatrix}\langle\alpha| \\ \langle\beta|\end{pmatrix}\hat{\mathbf{s}}\,(\,|\alpha\rangle \quad |\beta\rangle\,) = \begin{pmatrix}\langle\alpha|\hat{\mathbf{s}}|\alpha\rangle & \langle\alpha|\hat{\mathbf{s}}|\beta\rangle \\ \langle\beta|\hat{\mathbf{s}}|\alpha\rangle & \langle\beta|\hat{\mathbf{s}}|\beta\rangle\end{pmatrix} = \frac{\hbar}{2}\boldsymbol{\sigma}$$

for $l > u \gg 0$, where u is typically one more than the highest l found in the core

$$v_{l,l-1/2}(r) \doteq v_{l,l+1/2}(r) \doteq v_l(r) \doteq v_u(r)$$

avoiding infinite summation by exploiting the completeness relation for the projection operators $\hat{P}_l$

$$\Delta \mathrm{v}(r) \doteq \sum_{l=0}^{u-1} \sum_{j=\left|l-\frac{1}{2}\right|}^{l+\frac{1}{2}} v_{lj}(r)\mathrm{P}_{lj} + v_u(r) \sum_{l=u}^{\infty} \hat{P}_l$$

$$= \sum_{l=0}^{u-1} \sum_{j=\left|l-\frac{1}{2}\right|}^{l+\frac{1}{2}} v_{lj}(r)\mathrm{P}_{lj} + v_u(r) \sum_{l=u}^{\infty} \hat{P}_l + v_u(r) \left[1 - \sum_{l=0}^{\infty} \hat{P}_l\right]$$

$$= \sum_{l=0}^{u-1} \sum_{j=\left|l-\frac{1}{2}\right|}^{l+\frac{1}{2}} v_{lj}(r)\mathrm{P}_{lj} + v_u(r) - v_u(r) \sum_{l=0}^{u-1} \hat{P}_l$$

$$= \sum_{l=0}^{u-1} \sum_{j=\left|l-\frac{1}{2}\right|}^{l+\frac{1}{2}} v_{lj}(r)\mathrm{P}_{lj} + v_u(r) - v_u(r) \sum_{l=0}^{u-1} \sum_{j=\left|l-\frac{1}{2}\right|}^{l+\frac{1}{2}} \mathrm{P}_{lj}$$

$$= v_u(r) + \sum_{l=0}^{u-1} \sum_{j=\left|l-\frac{1}{2}\right|}^{l+\frac{1}{2}} \left[v_{lj}(r) - v_u(r)\right] \mathrm{P}_{lj}$$

$$= v_u(r) + [v_{0,1/2}(r) - v_u(r)]\mathrm{P}_{0,1/2}$$

$$+ \sum_{l=1}^{u-1} \left\{ \left[v_{l,l-1/2}(r) - v_u(r)\right] \mathrm{P}_{l,l-1/2} + \left[v_{l,l+1/2}(r) - v_u(r)\right] \mathrm{P}_{l,l+1/2} \right\}$$

$$= v_u(r) + [v_{0,1/2}(r) - v_u(r)]\hat{P}_0$$

$$+ \sum_{l=1}^{u-1} \left\{ \frac{l}{2l+1} \left[v_{l,l-1/2}(r) - v_u(r)\right] + \frac{l+1}{2l+1} \left[v_{l,l+1/2}(r) - v_u(r)\right] \right\} \hat{P}_l$$

$$+ \sum_{l=1}^{u-1} \left\{ \frac{1}{2l+1} \left[v_{l,l+1/2}(r) - v_u(r)\right] - \frac{1}{2l+1} \left[v_{l,l-1/2}(r) - v_u(r)\right] \right\} \mathrm{R}_l$$

$$= v_u(r) + [v_{0,1/2}(r) - v_u(r)]\hat{P}_0$$

$$+ \sum_{l=1}^{u-1} \left\{ \frac{1}{2l+1} \left[l v_{l,l-1/2}(r) + (l+1) v_{l,l+1/2}(r)\right] - v_u(r) \right\} \hat{P}_l$$

$$+ \sum_{l=1}^{u-1} \frac{1}{2l+1} \left[v_{l,l+1/2}(r) - v_{l,l-1/2}(r)\right] \mathrm{R}_l$$

$$= v_u(r) + \sum_{l=0}^{u-1} \left[v_l(r) - v_u(r)\right] \hat{P}_l + \sum_{l=1}^{u-1} \frac{1}{2l+1} \Delta v_l(r) \mathrm{R}_l$$

spin averaged

$$v_0(r) = v_{0,1/2}(r)$$

for $l \in \langle 1, u)$

$$v_l(r) = \frac{1}{2l+1} \left[l v_{l,l-1/2}(r) + (l+1) v_{l,l+1/2}(r)\right]$$

$$\Delta v_l(r) = v_{l,l+1/2}(r) - v_{l,l-1/2}(r)$$

convenient analytical form of both $v_0(r)$ and $v_{l,l\mp 1/2}(r)$, and thus also of $v_l(r)$ and $\Delta v_l(r)$, $l > 0$, is

$$\frac{1}{r^2}\sum_{i=1}^{k} c_i r^{n_i} e^{-\alpha_i r^2}$$

$$n_i \in N$$

$$c_i, \alpha_i \in R$$

$$k \approx 4$$

$$n_i \in \{0, 1, \mathbf{2}, \mathit{3}, 4, \ldots\}$$

$$\alpha_i > 0$$

Cartesian Gaussian functions

$$\xi(m, n, q, \alpha; \mathbf{r}) = x^m y^n z^q e^{-\alpha r^2} = x^m e^{-\alpha x^2} y^n e^{-\alpha y^2} z^q e^{-\alpha z^2}$$

nonredundant Cartesian Gaussian functions

$$\chi(l, m, \alpha; \mathbf{r}) = r^l e^{-\alpha r^2} S_{lm}(\vartheta, \varphi)$$

complex solid harmonics

$$r^l Y_{lm}(\vartheta, \varphi)$$

real solid harmonics

$$r^l S_{lm}(\vartheta, \varphi)$$

normalized atomic orbitals

$$2\left[\frac{(2\alpha)^3}{\pi}\right]^{\frac{1}{4}} \sqrt{\frac{2^l}{(2l+1)!!}} \left(\sqrt{2\alpha} r\right)^l e^{-\alpha r^2} S_{lm}(\vartheta, \varphi) = \frac{2^{l+1}}{\sqrt{(2l+1)!!}} \left(\frac{8}{\pi}\right)^{\frac{1}{4}} \alpha^{\frac{2l+3}{4}} \chi(l, m, \alpha; \mathbf{r})$$

the contribution of the atom to matrix elements of the effective spin–orbit Hamiltonian is calculated as a linear combination of one-center integrals

$$\int_{-\infty}^{\infty}\int_{-\infty}^{\infty}\int_{-\infty}^{\infty} \chi(k, p, \alpha; \mathbf{r})|l; q\rangle\langle l; q|\hat{l}_u|l; m\rangle\langle l; m|\chi(o, t, \beta; \mathbf{r}) r^{n-2} e^{-\gamma r^2}\, dx\, dy\, dz$$

$$= \langle l; q|\hat{l}_u|l; m\rangle \int_0^{\infty} r^{k+n+o} e^{-(\alpha+\beta+\gamma)r^2}\, dr \int_0^{\pi}\left[\int_0^{2\pi} S_{kp}(\vartheta, \varphi) S_{lq}(\vartheta, \varphi)\, d\varphi\right] \sin\vartheta\, d\vartheta$$

$$\times \int_0^{\pi}\left[\int_0^{2\pi} S_{lm}(\vartheta, \varphi) S_{ot}(\vartheta, \varphi)\, d\varphi\right] \sin\vartheta\, d\vartheta$$

$$= \delta_{kl}\delta_{lo}\delta_{pq}\delta_{mt}\langle l; q|\hat{l}_u|l; m\rangle \int_0^{\infty} r^{2l+n} e^{-(\alpha+\beta+\gamma)r^2}\, dr$$

$u = x, y, z$

for $n \geq 0$ and $a > 0$

$$\int_0^{\infty} x^{2n} e^{-ax^2} dx = \frac{1}{2}\left(\frac{\pi}{a}\right)^{\frac{1}{2}} \frac{(2n-1)!!}{(2a)^n}$$

$$\int_0^\infty x^{2n+1} e^{-ax^2} dx = \frac{n!}{2a^{n+1}}$$

solution in a closed form

$$\frac{2^{2l+2}}{(2l+1)!!} \left(\frac{8}{\pi}\right)^{\frac{1}{2}} (\alpha\beta)^{\frac{2l+3}{4}} \int_0^\infty r^{2l+n} e^{-(\alpha+\beta+\gamma)r^2}\, dr = \dots$$

for even n

$$\dots = \frac{2^{2l+2}}{(2l+1)!!} \left(\frac{8}{\pi}\right)^{\frac{1}{2}} (\alpha\beta)^{\frac{2l+3}{4}} \frac{1}{2} \left(\frac{\pi}{\alpha+\beta+\gamma}\right)^{\frac{1}{2}} \frac{(2l+n-1)!!}{[2(\alpha+\beta+\gamma)]^{l+\frac{n}{2}}}$$

$$= 2^{\frac{2l-n+5}{2}} \frac{(2l+n-1)!!}{(2l+1)!!} \frac{(\alpha\beta)^{\frac{2l+3}{4}}}{(\alpha+\beta+\gamma)^{\frac{2l+n+1}{2}}}$$

special case for $n = 2$

$$\dots = 2^{\frac{2l+3}{2}} \frac{(\alpha\beta)^{\frac{2l+3}{4}}}{(\alpha+\beta+\gamma)^{\frac{2l+3}{2}}} = \left[\frac{2(\alpha\beta)^{\frac{1}{2}}}{\alpha+\beta+\gamma}\right]^{\frac{2l+3}{2}}$$

for odd n

$$\dots = \frac{2^{2l+2}}{(2l+1)!!} \left(\frac{8}{\pi}\right)^{\frac{1}{2}} (\alpha\beta)^{\frac{2l+3}{4}} \frac{\left(\frac{2l+n-1}{2}\right)!}{2(\alpha+\beta+\gamma)^{\frac{2l+n+1}{2}}}$$

$$= 2^{\frac{4l+5}{2}} \frac{\left(\frac{2l+n-1}{2}\right)!}{(2l+1)!!} \frac{(\alpha\beta)^{\frac{2l+3}{4}}}{(\alpha+\beta+\gamma)^{\frac{2l+n+1}{2}}}$$

In spite of all the uncommon features of our program, it turns out that the time spent by the calculation of spin–orbit couplings between multielectron wave functions is still a negligible fraction of the time needed for carrying out spin forbidden nonadiabatic molecular dynamics simulation as a whole.

References

[1] J. Olsen and B. O. Roos and P. Jørgensen and H. J. Aa. Jensen J. Chem. Phys. 89 1988 2185–2192

[2] M. Kývala to be published

[3] P. -Å. Malmqvist Int. J. Quantum Chem. 30 1986 479–494

[4] T. Helgaker and P. Jørgensen and J. Olsen *Molecular Electronic-Structure Theory* Wiley Chichester 2000 569–573

[5] M. Kývala Int. J. Quantum Chem. 109 2009 1200–1207

[6] B. A. Hess and C. M. Marian and S. D. Peyerimhoff in *Modern Electronic Structure Theory Part I* D. R. Yarkony editor World Scientific Singapore 1995 152–278

[7] B. A. Hess and C. M. Marian and U. Wahlgren and O. Gropen Chem. Phys. Lett. 251 1996 365–371

[8] J. Chalupský and T. Yanai J. Chem. Phys. 139 2013 204106

[9] M. Kývala unpublished

[10] B. Schimmelpfennig PhD Thesis Stockholm University 1996

[11] N. Matsunaga and S. Koseki in *Reviews in Computational Chemistry Volume 20* K. B. Lipkowitz and R. Larter and T. R. Cundari editors John Wiley & Sons New Jersey 2004 101–152

[12] Y. S. Lee and W. C. Ermler and R. M. Pitzer J. Chem. Phys. 67 1977 5861–5876

[13] R. M. Pitzer and N. W. Winter J. Phys. Chem. 92 1988 3061–3063

[14] T. R. Cundari and M. T. Benson and M. L. Lutz and S. O. Sommerer in *Reviews in Computational Chemistry Volume 8* K. B. Lipkowitz and D. B. Boyd editors WCH Publishers New York 1996 145–202

[15] M. Dolg in *Relativistic Electronic Structure Theory Part 1. Fundamentals* P. Schwerdtfeger editor Elsevier Amsterdam 2002 793–862

[16] P. Schwerdtfeger in *Theoretical Chemistry and Physics of Heavy and Superheavy Elements* U. Kaldor and S. Wilson editors Kluwer Academic Publishers Dordrecht 2003 399–438

[17] K. G. Dyall and K. Fægri, Jr. *Introduction to Relativistic Quantum Chemistry* Oxford University Press New York 2007 396–426

[18] X. Cao and M. Dolg in *Relativistic Methods for Chemists* M. Barysz and Y. Ishikawa editors Springer Dordrecht 2010 215–277

[19] P. Schwerdtfeger ChemPhysChem 12 2011 3143–3155

[20] M. Dolg and X. Cao Chem. Rev. 112 2012 403–480
